# Analysis of the fermentation kinetics and gut microbiota modulatory effect of dried chicory root reveals the impact of the plant-cell matrix rationalizing its conversion in the distal colon

**DOI:** 10.20517/mrr.2024.04

**Published:** 2024-04-26

**Authors:** Marie-Luise Puhlmann, Ember van de Rakt, Evangelia N. Kerezoudi, Ignacio Rangel, Robert J. Brummer, Hauke Smidt, Frederik S. Kaper, Willem M. de Vos

**Affiliations:** ^1^Laboratory of Microbiology, Wageningen University & Research, Wageningen 6708 WE, the Netherlands.; ^2^Division of Human Nutrition and Health, Wageningen University & Research, Wageningen 6708 WE, the Netherlands.; ^3^Nutrition-Gut-Brain Interactions Research Centre, School of Medical Sciences, Faculty of Medicine and Health, Örebro University, Örebro 70182, Sweden.; ^4^Department of Nutrition and Dietetics, Harokopio University, Athens 17671, Greece.; ^5^WholeFiber BV, Emmeloord 8300 BA, the Netherlands.; ^6^Human Microbiome Research Program, Faculty of Medicine, University of Helsinki, Helsinki 00014, Finland.

**Keywords:** Plant cell wall, chicory root, intrinsic fiber, gut health, gut microbiota, colonic fermentation, butyrate production

## Abstract

**Aim:** The cell matrix of plant foods has received little attention in prebiotic fiber research. We aimed to understand the impact of the plant cell matrix in dried chicory root on its breakdown in the human gut to explain its reported beneficial effects on gut and metabolic health.

**Methods:** We applied *in vitro* digestion and fermentation models together with an *ex vivo* gut barrier integrity model. Plant cell matrix intactness in the upper gastrointestinal tract was investigated by scanning electron microscopy. Colonic breakdown of inulin, and chicory root cubes and powder was assessed by gut microbiota analysis using 16S rRNA gene amplicon sequencing and determining the kinetics of changes in pH, gas, and short-chain fatty acid (SCFA) production. Finally, effects on gut barrier integrity were explored by exposing colonic biopsies to fermentation supernatants in an Ussing chamber model.

**Results:** The plant cell matrix of dried chicory root cubes remained intact throughout upper gastrointestinal transit. Dried chicory root fermentation resulted in higher final relative abundances of pectin-degrading *Monoglobus* and butyrate-producing *Roseburia* spp. compared to inulin and a seven-fold increase in *Bifidobacterium* spp. in donors where these species were present. Dried chicory root cubes yielded similar total SCFAs but higher final butyrate levels than chicory root powder or isolated inulin with less gas produced. No uniform but donor-specific effects of fermentation supernatants on the maintenance of gut barrier integrity were detected.

**Conclusion:** The intact plant cell matrix of dried chicory root affected its colonic breakdown kinetics and microbiota, underpinning its beneficial effect *in vivo*.

## INTRODUCTION

Dietary fibers are omnipresent in human food products and play a fundamental role in maintaining human health. The health benefit of fibers was originally attributed to their physicochemical properties, which improve satiety by water-binding and regulate lipid homeostasis by binding cholesterol and bile acids^[1]^. However, research of the past two decades revealed that a large part of the beneficial effect of fibers is mediated by the human gastrointestinal tract microbiota. As dietary fibers are, by definition, compounds that cannot be broken down by human endogenous enzymes, they reach the lower gut undigested^[2,3]^. There, they are used as substrates by the human gut microbiota, a collective of fungi, protozoa, archaea, and predominantly bacteria that have the enzymatic machinery to break down dietary fibers and thereby produce a range of microbial metabolites^[4]^. The interaction between the gut microbiota, its metabolites, and the human body mediates the beneficial effect of fibers on human health beyond their physical interaction with the upper gastrointestinal tract. A major group of fiber-derived bacterial metabolites are short-chain fatty acids (SCFAs), including acetate, propionate, and butyrate, that signal to GPR receptors and may have local or systemic effects^[5]^. Notably, butyrate has been recognized to be essential for maintaining human gut health as it serves as fuel for colonocytes, strengthens the gut lining, and improves gut barrier function^[6]^. That has led to increasing efforts to understand how butyrate production in the human colon can be stimulated using dietary fibers.

Approaches to studying fiber-microbiota relationships have progressively become reductionist with a focus on single fibers’ molecular breakdown^[7]^. One particularly studied fiber is inulin, known for its prebiotic effect, which means it is selectively used by human gut bacteria - notably bifidobacteria - thereby conferring health benefits^[3]^, contributing to a health claim for stool frequency maintenance^[8]^. Another well-studied group of fibers includes pectins that have been recognized to regulate postprandial glucose response, blood cholesterol levels, and satiety^[9]^, while some of their fragments have immune-modulatory benefits (RG-I^[10]^). For the purpose of establishing such structure-function relationships and health outcomes, fibers are often studied in isolation, which means they have been extracted and purified from the original plant food matrix. It is hypothesized that these isolated fibers are mainly fermented in the proximal colon with subsequently low levels of SCFAs in the distal colon, which favors fermentation of residual proteins^[11,12]^. While these reductionist approaches allow us to understand how specific fibers elicit specific microbial responses, they ignore how fibers are commonly consumed: intrinsically present in plants foods with each their unique plant cell matrix^[13,14]^. Fibers like pectin and inulin in plant foods make up an intrinsic, complex plant cell network in which pectin is intertwined with hemicellulose and cellulose, forming the structure of the plant cells encapsulating other non-structural fibers, such as inulin^[13,14]^. This complex network does not dissolve into its isolated compounds in the human gastrointestinal tract but rather functions as a vehicle transporting these fiber structures to the lower gut^[15,16]^. For these reasons, fibers originally present in the plant matrix have been termed intrinsic fibers to distinguish them from isolated fibers extracted from the plant cells^[14]^.

Chicory root is a root vegetable with a plant cell matrix encapsulating particularly high amounts of inulin inside the plant cell vacuoles. While chicory roots are nowadays mainly used for the production of isolated inulin, they have long been used as both a medicinal and a culinary root vegetable^[17]^. In its dried form, chicory root consists of up to 85% fiber, 70% of which is inulin, making it an excellent source of intrinsic dietary fiber^[17]^. We hypothesize that the presence of the plant cell wall can potentially function as a physical barrier, shielding inulin from immediate contact with the gut microbiota, thereby impacting intrinsic fiber’s breakdown kinetics and location in the human gut. Food processing steps such as particle size reduction^[18-22]^ or thermal treatment^[19,23]^ have the potential to affect gut bacteria accessibility and related breakdown kinetics due to the induced damage of the plant cell matrix. Until now, *in vitro* assessments of plant food breakdown kinetics have focused on pectin-, starch- and lipid-containing whole foods^[20,24-26]^ but never inulin-rich vegetables like chicory root. Previously, we have shown in a placebo-controlled human trial that the intake of dried chicory root particles dramatically modulated gut microbiota composition by stimulating a trophic chain involving members of *Bifidobacterium* spp. and *Anaerostipes* spp. toward butyrate production and improved both gut and metabolic health^[27]^. We attributed these changes to a slow release of fibers from dried chicory root particles. This would prolong the fermentation of the dried chicory root particles rationalizing a rather distal location of their breakdown. In humans, slow and gradual fiber fermentation has been hypothesized to benefit gut health by distributing fiber fermentation from the proximal throughout the distal colon^[12]^. Moreover, SCFAs delivered to the distal colon have been shown to confer more pronounced systemic health benefits compared to a proximal delivery^[28,29]^.

**Figure 1:**
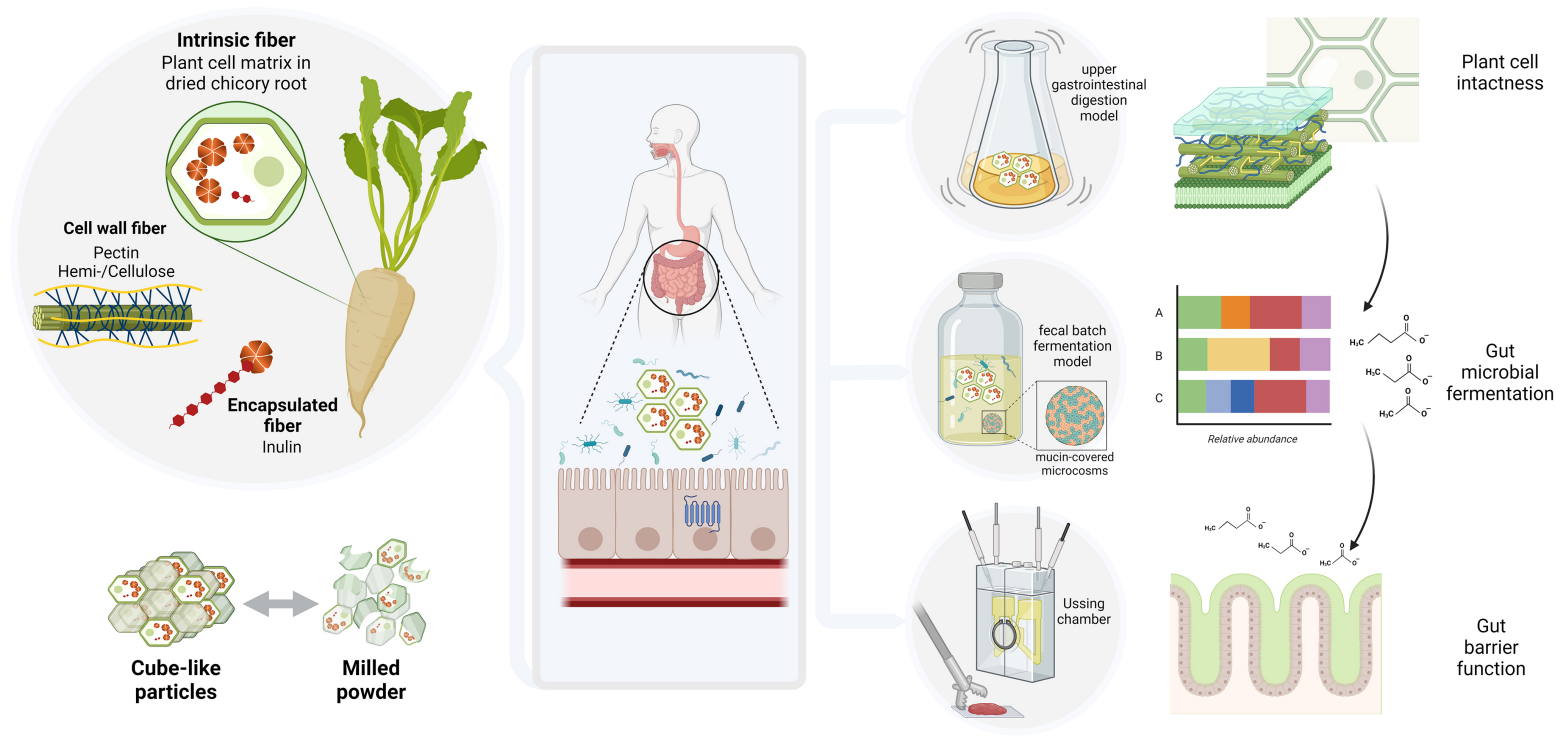
Schematic depiction of the study approach to understand how the plant cell matrix in dried chicory root cubes and powder affects its upper digestion and lower gut microbial fermentation kinetics and subsequent interaction with the gut barrier. An upper *in vitro* digestion model was used to assess the intactness of the plant cell matrix upon entering the lower intestine (colon), where, using an *in vitro* fecal batch fermentation model, we studied gut microbial breakdown. Finally, we investigated how the fermentation metabolites potentially interacted with the human colonic epithelium using an Ussing chamber. Created with BioRender.com.

Our aim was to assess whether the plant cell matrix of dried chicory root remains intact in the upper gastrointestinal tract and how its presence affects lower gut microbial composition and fermentation kinetics, as well as the potential effect of dried chicory root fermentation supernatants on gut barrier integrity. For this purpose, we executed a series of experiments using *in vitro* and *ex vivo* models for the upper and lower gastrointestinal tract that were primed with dried chicory root particles with two different degrees of cell wall intactness in the form of cubes and milled into powder [Figure 1].

## METHODS

### Dried chicory root

Dried chicory root was provided by WholeFiber BV (Emmeloord, the Netherlands). The product is made from chicory roots that have been washed, cut, and dried, producing cube-like pieces of approximately 3 mm rib. The final product has a dry weight of 93%w/w, of which 70%w/w is native inulin, 10%w/w pectin, 5%w/w hemicellulose and cellulose, and 4%w/w mono- and disaccharides, 5%w/w proteins, and remaining minerals, polyphenols, and vitamins. To assess whether dried chicory root in its structure was similar to fresh, unprocessed chicory root, fresh chicory root was also provided by WholeFiber BV. To study the effect of particle size, dried chicory root cubes were ground to a mean particle size of < 0.5 mm.

### Upper gastrointestinal digestion model

To assess potential physical changes during upper gastrointestinal digestion, we mimicked digestive processes during the oral, gastric, and small intestinal phases using an adapted version of the INFOGEST protocol^[30,31]^. Experiments were executed in triplicate and samples were taken at the end of the oral phase (2 min) and after 30, 60, and 120 min of both the gastric and small intestinal digestion by separating the liquid from the solid digesta using a sieve. Liquid digesta were heat-treated and snap frozen in liquid nitrogen and solids were fixated in 70% ethanol for immediate image processing. We visualized the plant cell matrix of fresh and dried undigested and digested chicory root cubes using scanning electron microscopy (SEM) and light microscopy to assess structural changes. Potential leakage of pectin from the plant cell matrix was estimated by measuring uronic acid concentration in the gastric and small intestinal phase liquid digesta using an automated colorimetric *m*-hydroxydiphenyl assay^[32]^ and expressing measured levels as a percentage of an expected total uronic acid content (5%w/w) in chicory root as determined according to Ramasamy *et al.*^[33]^. Potential differences between inulin from dried chicory root powder versus cubes leaking into digestive fluids were estimated by measuring mono-/disaccharides and fructan-oligomers and polymers of different chain lengths [degree of polymerization (DP)] using High Performance Anion Exchange Chromatography (HPAEC) according to established methods^[34]^. Details on the adapted INFOGEST method and the pectin and inulin measurements can be found in the Supplementary Materials.

### Lower gastrointestinal fermentation model

To investigate the effect of the presence and intactness of the plant cell wall matrix on the lower gut microbial fermentation of the dried chicory root, a fecal *in vitro* batch fermentation experiment was performed at ProDigest (Gent, Belgium) using dried chicory root cubes, powder, and isolated chicory inulin as a comparison. For this purpose, the feces of a healthy human donor with low bifidobacteria count (assessed by qPCR to be < 10^8^ 16S rRNA gene copies/g corresponding to a relative abundance of < 0.01%) was chosen. This was done to exemplify how such a gut microbiota would respond to an intrinsic fiber product since high baseline bifidobacteria levels reportedly affect related fiber responses^[35-38]^. Experiments were executed in triplicate and samples were taken at baseline (t = 0 h) after 6, 24, and 48 h. Details on the execution of the experiment including mucin-covered beads^[39]^ and the subsequent analysis of gut microbiota composition in fermentation pellets, pH and gut microbial metabolites SCFAs, lactate, ammonium, and branched-chain fatty acids (BCFAs) in fermentation supernatants, and gas production are given in the Supplementary Materials.

### Human gut barrier function model - Ussing chamber

To explore the possible effects of fermentation products on gut barrier function, we performed an *ex vivo* Ussing chamber experiment using human colonic biopsies according to previously described methods^[40]^. Biopsies from four healthy donors (mean age 42 years) were obtained by endoscopy without prior bowel cleansing and exposed to fermentation supernatants from *in vitro* fermentation of dried chicory root cubes. The same donor’s feces were used to obtain fermentation supernatants for the respective colonic biopsy to study the individual interaction between the donor’s gut epithelium and their gut microbiota-induced fiber fermentation. Changes in transepithelial resistance (TER) (measure of overall gut integrity^[41]^) and paracellular permeability (assessed by Fluorescein isothiocyanate-dextran concentration; assessing passage between cells) in control biopsies over time were compared at 60 and 90 min to biopsies previously exposed (20 min) to the fermentation supernatant and biopsies stressed with sodium deoxycholate (SDC) as well as biopsies both exposed to the fermentation supernatant and stressed with SDC. Details on the human donors, the fecal *in vitro* batch fermentation model, and analysis of the gut microbiota composition in fermentation pellets, pH and gut microbial metabolites in fermentation supernatants, and gas production are given in the Supplementary Materials.

### Statistical analysis

Data were analyzed using R version 4.2.3^[42]^. Normality was checked by inspecting QQ plots. Descriptive statistics were calculated using the rstatix package^[43]^ and data were expressed as mean with standard error of the mean. While the use of statistical hypothesis testing for small sample sizes, as commonly used in *in vitro* triplicate experiments, is debatable, statistical inference was made for the purpose of understandability. Differences in metabolites (SCFA, BCFA, lactate, and ammonium) and other outcomes (pH, gas production, α-diversity) between products at each time point were tested using robust ANOVA with corresponding post hoc and Benjamini-Hochberg correction from the WRS2 package^[44]^ and implemented in the ggstatsplot package^[45]^. Robust ANOVA was chosen as it can handle violations against normality and homoscedasticity and sample sizes were too small (triplicates) for reliable non-parametric testing implementing Chi-square distributions. Differences in gut integrity and permeability at each time point between conditions were tested using ggstatsplot within-subject ANOVA, taking into account the biopsies’ paired nature. Graphs were made using ggplot2^[46]^ or Microsoft Office 365 Excel. Gut microbiota outcomes were analyzed as described previously^[27]^ using the mare^[47]^ and vegan package^[48]^. Multivariate community analysis was done on the genus level by using Principal Coordinate Analysis (PCoA) and constructing a principal response curve based on Bray-Curtis dissimilarity (β-diversity), as well as calculating gut bacterial richness (α-diversity) based on the number of detected taxa. For univariate analysis at the genus level, taxa counts were converted into relative abundances (%) and differential abundance testing with false discovery rate (fdr) correction was performed as implemented in mare^[47]^.

## RESULTS

We employed a series of complementary *in vitro* and *ex vivo* gastrointestinal models primed with dried chicory root cube-like particles or milled into powder as to determine the intactness of the plant cell walls, its impact on gut microbial fermentation kinetics and composition, and finally the effect on gut barrier integrity in human colonic biopsies.

### Intactness of the plant cell wall in dried chicory root as assessed in an upper gastrointestinal *in vitro* digestion model

**Figure 2:**
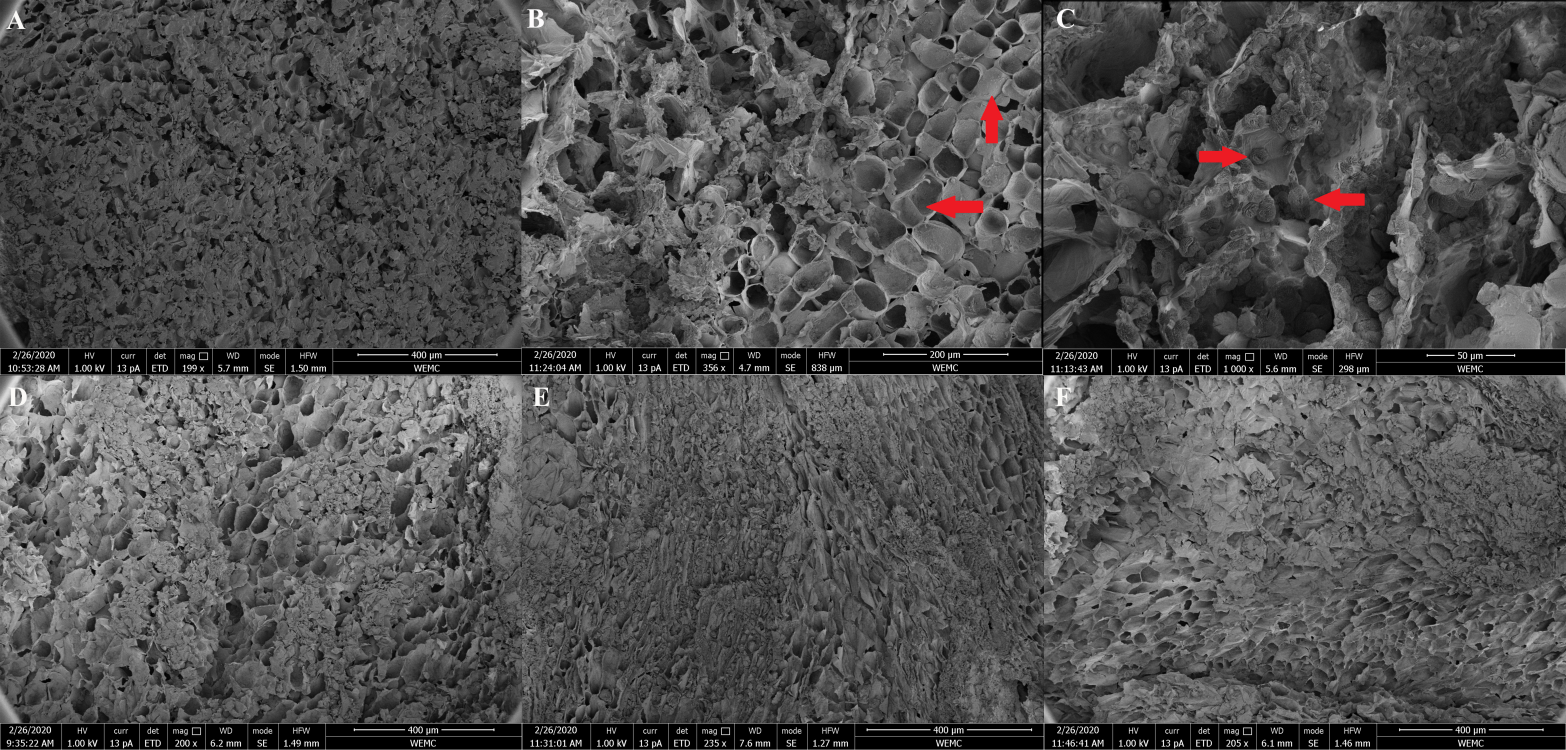
Scanning electron microscopy micrographs of fresh and digested dried chicory root. (A-C) plant cell matrix of (A) fresh chicory root with (B) intact plant cells and (C) intracellular inulin as indicated by red arrows; (D) intact plant cell matrix of dried chicory root cubes after rehydration prior to upper gastrointestinal digestion; (E) plant cell matrix after gastric digestion of dried chicory root cubes; (F) plant cell matrix after gastric and small intestinal digestion of dried chicory roots. While the macrostructure appeared to weaken over time [Supplementary Figure 2], the overall structure remained visually unchanged throughout the gastric and small intestinal phases, with no observed holes or cracks in the plant cell walls. Note the different magnifications in panels (B) and (C).

First, we set out to assess whether the plant cell matrix was still intact after drying the chicory root particles. Using SEM, we indeed observed that the overall plant cell structure was intact in both freshly cut chicory root pieces [Figure 2A-C] and rehydrated chicory root cubes [Figure 2D]. Plant cells were open [Figure 2B] with a small level of damage most likely resulting from cutting the plant particles for image preparation. Within the plant cells, inulin was clearly visible as a crystalline structure due to precipitation in ethanol [Figure 2C]. Next, we hydrated the chicory root products to mimic their consumption and conducted an upper *in vitro* digestion with an oral, gastric, and small intestinal phase using the well-established INFOGEST procedure^[30,31]^. After oral and gastric digestion with a pH lowered from 3 to 2 (to mimic the fasted state/end of gastric digestion), the overall plant structure remained largely intact, with densely packed plant cells filled with inulin [Figure 2E]. Similarly, at the end of the small intestinal phase, no obvious damage to plant cells in the form of holes or cracks in the cell wall was observed [Figure 2F]. The same was confirmed by light microscopy [Supplementary Figure 1]. However, throughout the gastric and small intestinal phase, the macrostructure [Supplementary Figure 2] weakened overall, with plant cells appearing less round and robust [Figure 2E and F]. To estimate how much pectin was potentially leaking from the plant cell structure of the dried chicory cubes compared to the powder, we measured uronic acid content as a proxy for pectin in the liquid part of gastric and small intestinal digesta. Estimated based on the total uronic acid (UA) content in dried chicory [Supplementary Figure 3], we found that on average (mean ± SD), 16.71% ± 2.91% of pectin (UA: 8.36 ± 1.46 mg/g product) leaked from dried chicory root powder, which was slightly higher than for chicory root cubes with 10.20% ± 0.70% of pectin (UA: 5.10 ± 0.35 mg/g product). Throughout the small intestinal phase, the leaked pectin increased for powder to 27.56% ± 0.73% (UA: 13.78 ± 0.37 mg/g product), which was nearly twice as high as for chicory root cubes with 14.48% ± 1.25% (UA: 7.24 ± 0.63 mg/g product). Besides pectin, we also investigated differences in inulin of various chain lengths (DP) detected in the digesta of dried chicory root powder and cubes by HPAEC chromatograms [Supplementary Figure 4]. We observed a consistently higher total area under the curve of the HPAEC chromatograms for dried chicory root powder compared to the cubes, representing all detected mono-/disaccharides and fructan oligomers and polymers. No changes in chain length distribution throughout the digestion phases were observed, but dried chicory root powder had the highest amounts of fructose mono- and oligomers (DP2-5) and longer-chain inulin (> DP16) compared to dried chicory cubes.

### Differences in gut microbiota response to different particle sizes of dried chicory root compared to inulin

**Figure 3:**
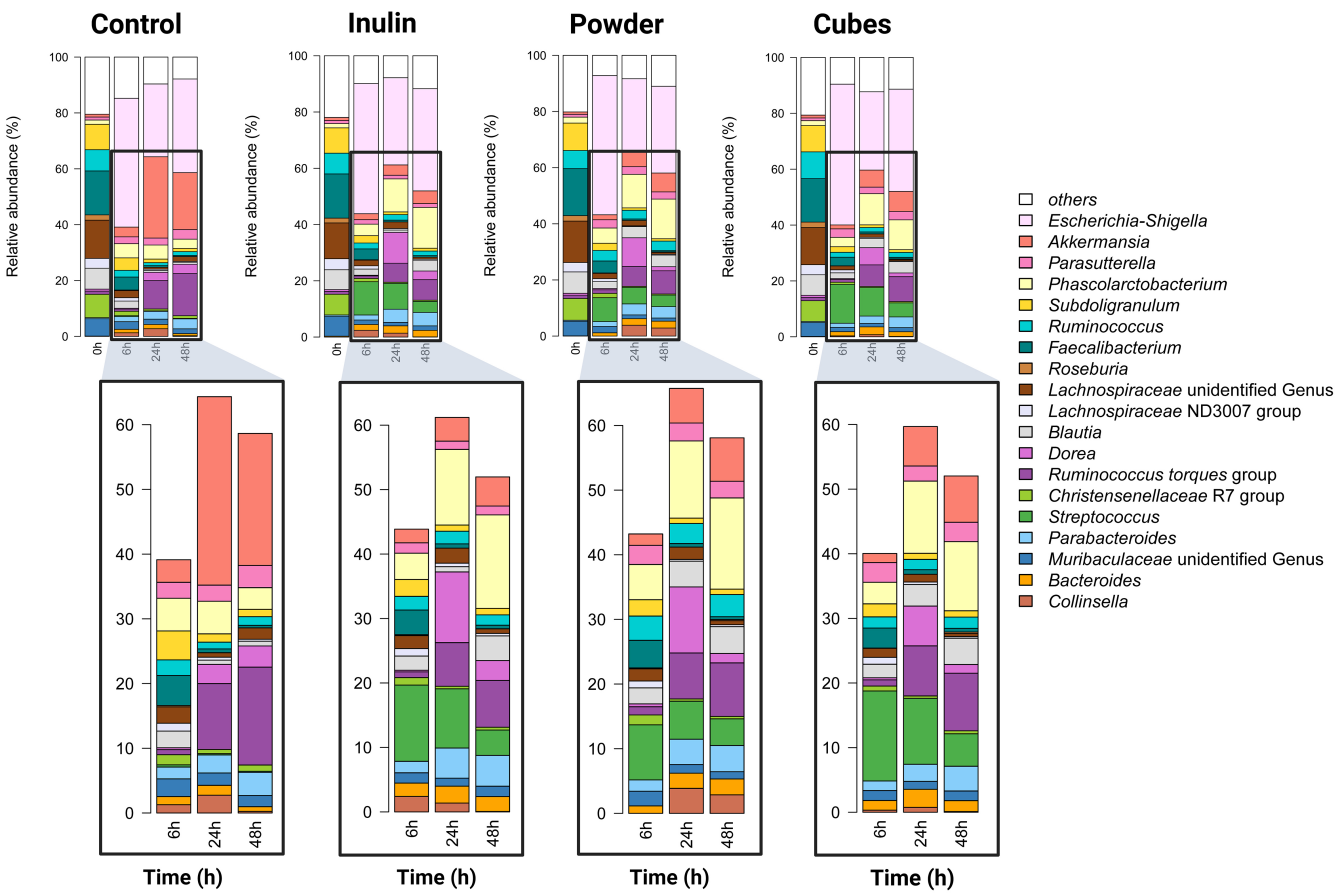
Microbiota composition observed during fecal batch *in vitro* fermentations using a donor low in *Bifidobacterium* spp.: Mean relative abundance (%) of common genera (at least 1% relative abundance and 50% prevalence in all samples) detected in *in vitro* fermentations over time (t = 0, 6, 24 and 48 h in the control (Control; only inoculum) and for inulin (Inulin) *vs.* dried chicory root powder (Powder) or cubes (Cubes). The lower panel zooms in on the common taxa, omitting the contribution of *Escherichia-Shigella* spp., which dominated the community between 0 and 6 h of fermentation.

Following the assessment of changes in the upper gastrointestinal tract, dried chicory root cubes and powder were subjected to breakdown by the gut microbiota and compared to inulin using a fecal batch fermentation experiment. At baseline, all samples had a similar microbiota composition as determined by 16S rRNA gene amplicon sequence analysis [Supplementary Figure 5]. In line with the selection of a donor with a low bifidobacteria count, no *Bifidobacterium* spp. were detected at baseline [Figure 3]. Following inoculation, we observed a rapid increase in *Escherichia-Shigella* spp. in all conditions (control, dried chicory root cubes and powder and inulin), which was highest at 6 h, making up approximately half the microbial community, and decreasing to about 30% at 48 h [Supplementary Table 1]. These changes were not statistically significantly different between conditions [Supplementary Table 2] and likely represent a model-induced outgrowth of this facultative aerobic bacteria taxon.

When addressing changes in the overall microbiota composition, we observed a rapid modulation in all conditions within 6 h, which peaked at 24 h before leveling off at 48 h (between-sample β-diversity analysis based on Bray-Curtis dissimilarity, Supplementary Figure 5 and within-sample α-diversity analysis based on gut bacteria richness, Supplementary Figure 6). These time-dependent changes were reflected in the changes in relative abundance levels of individual gut microbiota members over time [Figure 3]. At the genus level, numerous taxa in all conditions decreased rapidly within 6 h and leveled off at 24 h of fermentation, resulting in the largest observed fold changes in this time period [Supplementary Table 1]. Particularly, *Streptococcus* spp. peaked at 6 h with significantly higher levels for dried chicory root cubes (14.9%) compared to inulin (11.8%). In addition, *Parasutterella* spp. increased to their highest levels at 6 h in dried chicory root cubes (3.3%) and powder (3.0%; Supplementary Table 1) and had still statistically significantly higher levels at 24 h compared to inulin [Supplementary Table 2]. A number of genera first decreased between 0 to 6 h but then re-increased between 6 and 24 and 48 h. Notably, bacteria from the *Eubacterium hallii* group (now also known as *Anaerobutyricum* spp.^[49]^) decreased in all conditions and were not detected at 6 or 24 h, but then re-increased above baseline levels at 48 h in inulin (4.5-fold) and dried chicory root powder (1.8-fold) and cubes (2.2-fold). *Monoglobus* spp. also decreased in all conditions, but re-increased from 6 to 48 h only for dried chicory root powder and cubes, despite reaching (slightly) lower levels than at baseline. The same was true for bacteria from the *Eubacterium eligens* group and *Roseburia* spp. [Supplementary Table 1], which were, after 48 h, significantly higher in powder and cubes due to their virtual absence in inulin and control fermentations [Supplementary Table 2]. Taking all increases in all taxa together, we observed the highest number of significantly increasing genera at 48 h for dried chicory root cubes, with 15 genera increasing in relative abundance compared to inulin with 12 genera increasing and powder and control with 11 genera [Supplementary Table 3].

The used system with mucin-covered beads also allowed for the analysis of the mucus-adhering microbiota, of which notably *Roseburia* spp. had significantly higher levels in dried chicory root powder and cubes after 48 h of fermentation [Supplementary Figure 7 and Supplementary Table 2]. The use of mucin-covered beads also explains the increase in relative abundance of mucin-degrading *Akkermansia* spp. in control incubations lacking additional fermentable substrates.

### Differences in pH, gas production and fermentation metabolites of different particle sizes of dried chicory root compared to inulin

**Figure 4:**
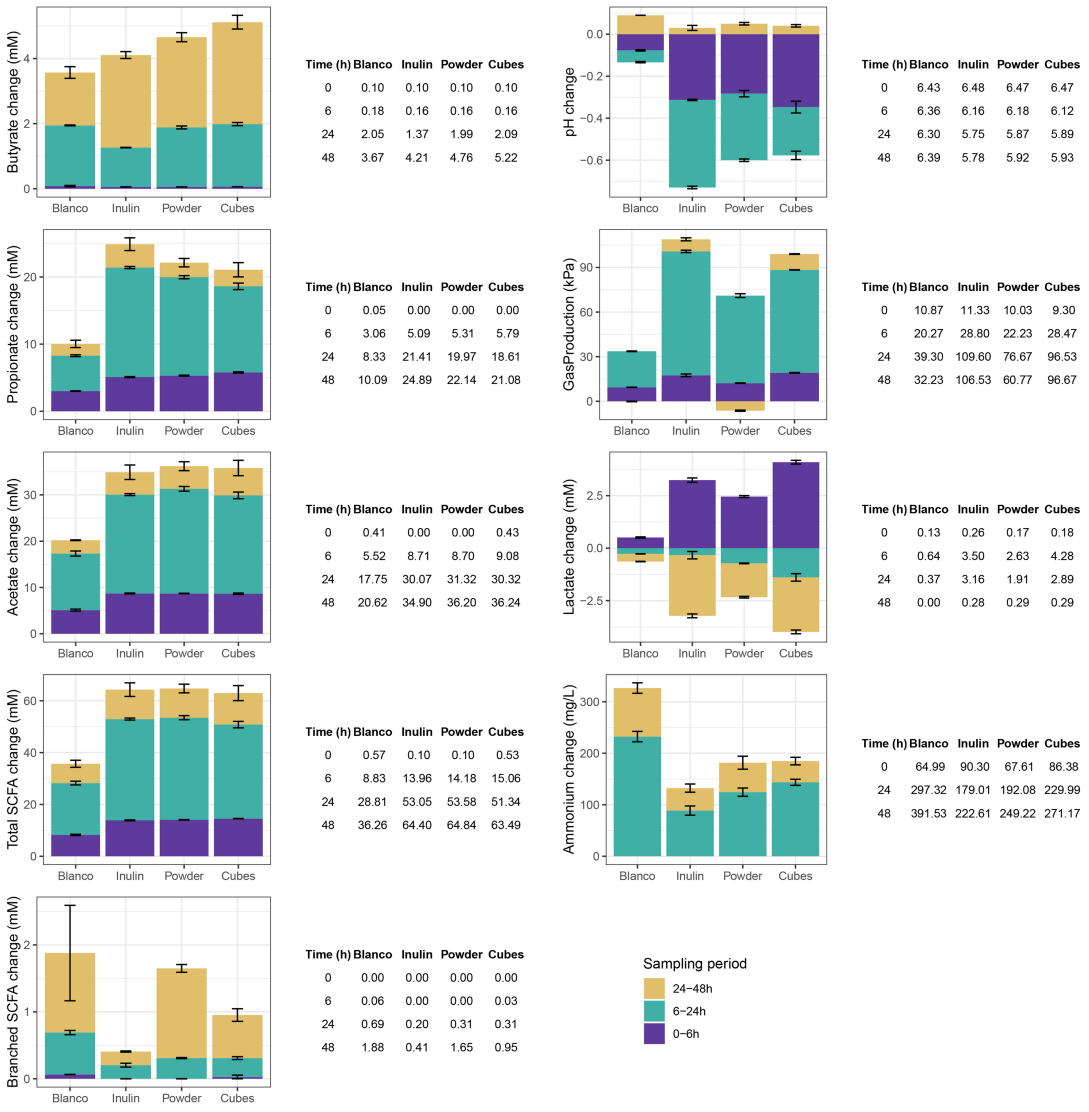
Fermentation metabolites, pH, and gas production measured during *in vitro* fermentation of control (Control; only inoculum), inulin (Inulin), and dried chicory root powder (Powder) and cubes (Cubes) at baseline (t = 0 h), 6 h, 24 h, and 48 h and the respective consecutive changes between each sampling period. Details on statistical testing are provided in Supplementary Table 4.

Concomitantly, with the time-dependent development in the microbiota composition, we also observed changes in pH, gas production, and metabolites such as SCFAs and other organic acids produced [Figure 4]. The pH decreased in all conditions from 0 to 6 and 24 h, but then slightly re-increased at 48 h. Fermentation of inulin induced the largest pH decrease, especially at 6 and 24 h [Figure 4]. Consequently, inulin pH levels at 24 and 48 h were statistically significantly lower than those of the control, the dried chicory root powder and cubes [Supplementary Figure 8 and Supplementary Table 4]. Gas production increased rapidly and peaked at 24 h with inulin, resulting in the highest gas production between 0 and 48 h, which was statistically significantly higher than control and dried chicory root products [Figure 4 and Supplementary Table 4]. Along with pH and gas, we measured the SCFAs acetate, propionate, and butyrate. Butyrate started to increase between 6 and 24 h, but most butyrate was produced between 24 and 48 h for inulin and dried chicory root products. The highest butyrate levels were recorded at 48 h for dried chicory root cubes with (mean ± SE) 5.22 ± 0.21 mM, which differed statistically significantly from control (3.67 ± 0.18 mM, *P* = 0.031) and inulin (4.21 ± 0.11 mM, *P* = 0.049). Additionally, the increase in butyrate between 24 to 48 h was the highest for dried chicory root cubes (+3.13 ± 0.16 mM; Supplementary Table 4). Compared to inulin, the butyrate increase between 6 to 24 h was also statistically significantly higher for dried chicory root cubes (*P* = 0.009) and powder (*P* = 0.009; Supplementary Table 4). In contrast to the dynamics observed for butyrate, propionate increased substantially already between 0 to 6 h. At 6 h, propionate levels were statistically significantly highest for dried chicory root cubes but then at 24 h for inulin. At 48 h, propionate levels remained higher for inulin despite not differing anymore from dried chicory root products [Figure 4 and Supplementary Table 4]. Similar to propionate, acetate also increased rapidly from 6 h onwards, reaching the highest levels at 48 h, but not differing between inulin and dried chicory root products [Supplementary Table 4]. As acetate is the most abundant SCFA, its changes also dominated the observed increases in total SCFAs, which were similar for inulin and dried chicory root products [Figure 4]. Lactate also increased rapidly between 0 and 6 h, with the highest levels at 6 h for dried chicory root cubes that differed statistically significantly from control, inulin, and dried chicory root powder. Between 6 h and 24 h, lactate was partially consumed in all conditions with the highest decrease in dried chicory root cubes (-1.39 ± 0.18 mM), which was statistically significantly different from inulin (-0.34 ± 0.18 mM, *P* = 0.003) but not dried chicory root powder (-0.72 ± 0.02 mM). At 48 h, lactate had largely disappeared in all conditions [Figure 4]. The production of BCFAs started from 6 h onwards and was the highest in the control, followed by dried chicory root powder, cubes, and inulin [Figure 4]. Yet, their levels only differed statistically significantly for dried chicory root powder and not between cubes and inulin at any time point [Supplementary Table 4]. Finally, ammonium increased mainly between 0 and 24 h and was highest for control and least for inulin, concomitant with the largest decrease in pH in inulin, while dried chicory root cubes and powder did not differ. In summary, dried chicory root cube fermentation yielded similar total SCFAs but higher final butyrate levels compared to chicory root powder or isolated inulin with less gas produced.

### Effect of fermentation supernatant produced from *in vitro* fermentation of dried chicory root on gut permeability

We continued to assess the effect of fermentation supernatants produced during the fermentation of dried chicory root on gut permeability in an *ex vivo* model using human colonic biopsies from four donors. Dried chicory root cubes were chosen as they were found to yield the highest butyrate levels *in vitro*.

#### Gut microbiota changes underlying the fermentation supernatants

**Figure 5:**
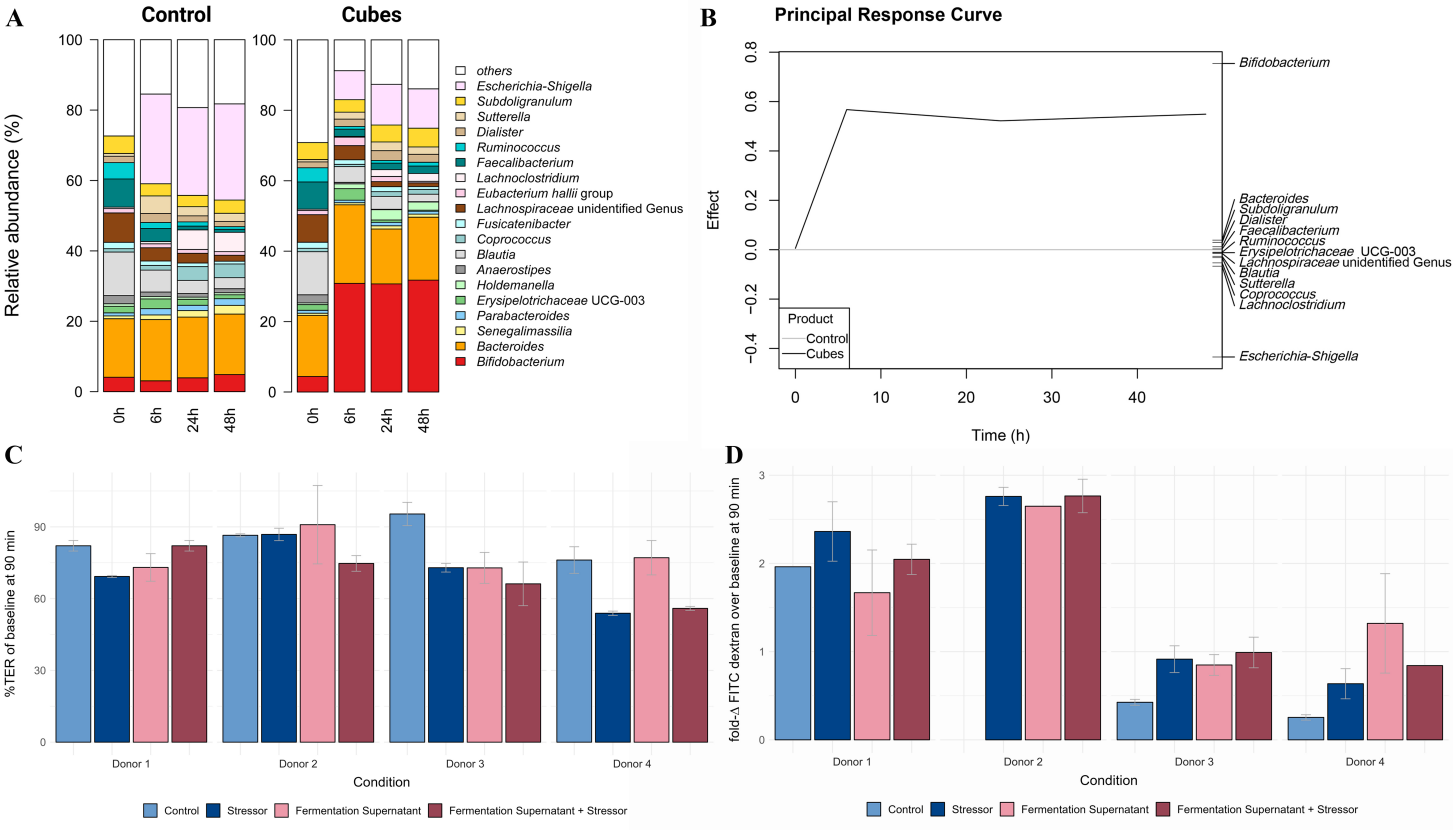
Gut microbiota composition of *in vitro* fermentations (A) and (B) to produce fermentation supernatants and their effect on colonic biopsies during the Ussing chamber experiment (C) and (D). (A) Common genera (mean relative abundance of at least 1% and mean prevalence of 50% in all samples) detected in the control (Control; only inoculum) and dried chicory root cube (Cubes) *in vitro* fermentation at baseline (T = 0), 6 h, 24 h, and 48 h; (B) Principal response curve assessing the combined response of all genera over time [relative abundance (%)] and thereby revealing genera differentiating between dried chicory root cube and control fermentation. Genera driving the difference are depicted on the right scale with taxa equally present in both dried chicory root cubes and control having small weights and taxa deviating most having larger weights; (C) Change in transepithelial resistance after 90 min as a percentage of baseline (%TER) in control or stimulated colonic biopsies using either the stressor SDC, the fermentation supernatant, or their combination (fermentation supernatant + stressor); (D) Change in FITC-dextran concentration between 0 and 90 min expressed as factor of the baseline concentration (fold-Δ). Information on all time points of all four donors can be found in Supplementary Figure 12. TER: Transepithelial resistance; %TER: TER at 90 min expressed as a percentage of each biopsies respective baseline; SDC: sodium deoxycholate; FITC: fluorescein isothiocyanate-dextran; fold-Δ FITC-dextran: relative change in FITC-dextran concentration between 0 to 90 min expressed as factor of each biopsies respective baseline (respective level expressed as factor of its baseline).

To produce the fermentation supernatant, dried chicory root cubes were again fermented in a fecal *in vitro* batch fermentation model and compared to a negative control (control; only inoculum), but at higher inoculum concentration. Again, we observed an increase in *Escherichia-Shigella* spp., which especially dominated the control fermentation [Figure 5A]. We observed a similar time-dependent modulation of the overall microbiota composition with changes in relative abundance levels of individual taxa mostly peaking at 6 h and leveling off at 24 h [Supplementary Table 5, Supplementary Figures 9 and 10]. In contrast to the single-donor *in vitro* fermentation, donors for this experiment were not chosen based on a low bifidobacteria count, and all four donors had considerable levels of *Bifidobacterium* spp. present in their feces (mean relative abundance across all conditions of 4.3% ± 1.5%; Figure 5A and Supplementary Figure 11). Following dried chicory root cube fermentation, we observed the highest changes at the genus level in *Bifidobacterium* spp. (6 h: *P* = 0.018, q = 0.216; 24 h: *P* = 0.081, q = 0.452; 48 h: *P* = 0.029, q = 0.152). *Bifidobacterium* spp. levels increased rapidly up to a third of the whole microbiota community and were statistically significantly higher (up to 10-fold) from control levels at 6 and 48 h (Supplementary Table 6 and for individual microbiota profiles, Supplementary Figure 11). Constructing a principal response curve [Figure 5B], which assessed the combined response over time of gut bacterial genera to the product using redundancy analysis on the first principal component, confirmed that relative levels in *Bifidobacterium* spp. clearly differentiated the microbial community of dried chicory root cubes from the control. Besides the changes in *Bifidobacterium* spp. levels, changes in *Coprococcus* spp. levels differed, too, but reached the highest levels in the control. We also observed several taxa decreasing in relative abundance in both conditions, but notably, butyrate-producing genera decreased only in the control to statistically significantly lower levels [Supplementary Table 5]. *Butyricicoccus* spp. levels fluctuated but were statistically significantly higher at 24 h in the dried chicory root cube fermentations and virtually absent in the control at 48 h [Supplementary Table 6]. Again, we observed that *Roseburia* spp. re-increased at 48 h for dried chicory root cubes but not control, while bacteria from the *Eubacterium hallii/Anaerobutyricum*^[49]^ group increased only for cubes at 6 and 24 h but then decreased at 48 h, reaching lower levels than the control [Supplementary Tables 5 and 6]. The dynamics of these gut microbiota changes were in line with those observed for the single donor low in bifidobacteria while demonstrating the difference in gut microbiota modulation when *Bifidobacterium* spp. are present.

#### Changes in pH, gas production and fermentation metabolites underlying the fermentation supernatants

We determined pH, gas, and production of SCFAs to understand the changes underlying the metabolites present in the fermentation supernatants [Supplementary Table 7]. The pH dropped again rapidly within 6 h, while gas production leveled off already between 6 and 24 h. Dried chicory root cubes yielded, on average, 12.56 ± 1.50 mM butyrate, 12.12 ± 1.97 mM propionate, and 38.60 ± 2.03 mM acetate, which were up to three times higher than for the control fermentation [Supplementary Table 7]. Butyrate production was largest between 6 to 24 h with 8.04 ± 1.71 mM. We observed lactate to be produced at 6 h (5.83 ± 1.70 mM) during fermentation of dried chicory root cubes, but this metabolite was completely consumed at 24 h. Formate followed the same dynamics as lactate, while the BCFA iso-butyrate levels peaked in dried chicory root cubes at 24 h before decreasing again at 48 h [Supplementary Table 7]. The fermentation outcomes based on four different donors were similar to the previous experiment with a single donor but followed a faster kinetic related to the higher concentration of fecal inoculum used.

#### Changes in gut barrier integrity of colonic biopsies following the stimulation with fermentation supernatants

Fermentation supernatants at 48 h were used to test their effect on gut barrier integrity of human colonic biopsies in an *ex vivo* Ussing chamber model. Human colonic biopsies were stressed with SDC, a secondary bile acid known to increase paracellular gut permeability and thereby affect gut barrier integrity, simulating low-grade inflammation^[50-53]^. Based on the measured SCFA concentrations in the fermentation supernatant at 48 h, biopsies stimulated with the fermentation supernatant (dilution to 2% v/v) were on average exposed to 0.20 to 0.34 mM butyrate (mean ± SE: 0.2 ± 0.03 mM), 0.17 to 0.34 mM propionate (0.24 ± 0.04 mM), and 0.74 to 0.86 mM acetate (0.77 ± 0.04 mM). In all biopsies, TER, as a measure of overall gut integrity, decreased over time, with the smallest decrease after 90 min in the control biopsies (ΔTER = -2.09 ± 0.64), followed by the biopsies that were previously exposed to the fermentation supernatant (unstressed: ΔTER = -3.32 ± 0.50; stressed: ΔTER = -4.11 ± 0.55). Notably, in these exposed biopsies, the decrease in TER was smaller and less rapid (from 60 to 90 min) compared to the stressed biopsies without previous exposure to the fermentation supernatant (t = 90 min, ΔTER = -5.58 ± 1.79; Supplementary Table 8). As the baseline TER differed considerably between donors, we calculated the relative decrease in TER at 90 min (expressed as a percentage of baseline, %TER; Figure 5C). When biopsies were stressed with SDC, no uniform difference in %TER decrease was found between exposed and unexposed biopsies (stressor: 70.72% ± 6.77% *vs.* fermentation supernatant + stressor: 69.72% ± 5.63%). However, we observed considerable heterogeneity between donors in the response of unstressed biopsies exposed to the fermentation supernatant. Of these biopsies, for donors 2 and 4, the %TER decrease was similar or smaller when exposed to the dried chicory root fermentation supernatant compared to no exposure (control condition; Figure 5C). Notably, these two donors showed the highest increase in relative abundance of bifidobacteria.

Concomitantly, with the decrease in overall gut integrity, we also observed an increase in gut permeability [Supplementary Figure 12 and Supplementary Table 8] and heterogenous responses [Figure 5D]. Paracellular permeability assessed by serosal FITC-dextran concentration increased in all biopsies over time with the least increase again in the control biopsies [Supplementary Figure 12B]. FITC-dextran concentration and absolute change at 90 min over baseline were highest for biopsies previously exposed to the fermentation supernatant (stressed and unstressed; Supplementary Table 8). However, their relative change in FITC-dextran concentration from 0 to 90 min (expressed as factor over baseline) was similar to the biopsies stimulated only with the stressor SDC [Figure 5D and Supplementary Table 8].

None of these changes in TER and FITC-dextran concentrations were found to be statistically significantly different between stimulations [Supplementary Table 8]. In summary, in the Ussing chamber model, exposure to the fermentation supernatants in the presence of the stressor SDC had no uniform effect on the gut barrier integrity of human colonic biopsies, as assessed by TER and paracellular permeability. However, TER decreased less rapidly over time when exposed to the fermentation supernatant. Considering the observed heterogeneity between donors, we also addressed the individual responses, and in two donors, we found smaller relative TER decreases when biopsies were exposed to their fermentation supernatant, indicating donor-specific effects on maintaining overall gut integrity [Supplementary Figure 12].

## DISCUSSION

Here, we investigated how the plant cell matrix in dried chicory root impacts its breakdown in the human gut by assessing its intactness in the upper gastrointestinal tract and determining its microbial breakdown kinetics and effect on gut barrier integrity by a series of *in vitro* and *ex vivo* models. We observed that the plant cell matrix of cubes and powder of dried chicory root remained intact during upper gastrointestinal transit in the INFOGEST model. Dried chicory root rapidly modulated the microbial community, resulting in the highest butyrate levels for cubes, while also co-occurring with higher levels of *Roseburia* spp. and the pectin-degrader *Monoglobus* spp. at 48 h of fermentation. For donors with *Bifidobacterium* spp. present at baseline, we observed a seven-fold increase following dried chicory root cube fermentation compared to control. Using the fermentation supernatant of the dried chicory root cubes to stimulate human colonic biopsies in an Ussing chamber model did not uniformly prevent stressor-induced impairment. Instead, donor-specific differences in unstressed biopsies emerged, notably from two donors with the highest final bifidobacteria levels.

The cell matrix of plant foods is a complex structure made of cellulose strengthened by hemicelluloses and pectin intrinsically intertwined into plant cell walls that encapsulate other non-structural carbohydrates, macro- and micronutrients. Dried chicory root is a food product that is particularly high in fiber due to its intra-cellular inulin content being part of the intrinsic plant cell matrix^[13,17]^. While inulin is a fiber known to be easily fermentable by the human gut microbiota, the presence of the plant cell wall in dried chicory root forms a physical barrier that gut bacteria need to open to access intracellular inulin and other cellular components. Consequently, the breakdown of dried chicory root differs from that of isolated inulin. Dietary fibers are, by definition, not digested in the upper gastrointestinal tract, but they can still be affected by the prolonged incubation in digestive juices and gastric and small intestinal pH changes leading to, for instance, the dissolution of pectin^[54,55]^. We observed that an estimated 15% of the total pectin leaked from the dried chicory root matrix during the *in vitro* gastric and small intestinal digestion. This was nearly twice as high for chicory root powder than cubes, likely due to the larger damage of the plant cell matrix induced by milling, breaking more plant cells open. Pectin that leaks out from plant foods is believed to be mainly soluble pectin from the intercellular space, which glues the plant cells together, enforcing the overall plant cell matrix^[16,56]^. Indeed, concomitant with the leakage of pectin over time, we observed an overall weakening of the macrostructure. Nonetheless, no obvious damage to the plant cell matrix in the form of cracks or holes in plant cell walls was visible, which indicates that dried chicory root cubes are likely to arrive in the lower gastrointestinal tract as intact particles. A weakened plant cell matrix may favor the release of intracellular inulin, and we observed higher amounts of fructose-monomers and fructo-oligosaccharides, together with longer-chain fructan-polymers, for dried chicory root powder, which we hypothesize to represent and relate to the higher plant cell damage. Thus, it is likely that dried chicory root cubes function as a delivery system of inulin and pectin that remain primarily encapsulated inside the intact plant cells to reach the distal parts of the colon.

This plant matrix intactness challenges the breakdown by the gut microbiota as the opening of the plant cell wall requires the degradation of the chemically more complex pectins and hemicelluloses. Chemical complexity selects for the action of specialist bacteria that have the functional machinery to access and metabolize diverse sugar constituents^[7]^, and the presence of different dietary fibers slows their gut microbial breakdown^[57-59]^. Therefore, we hypothesized that the kinetics of microbiota-mediated fiber breakdown may differ in dried chicory roots compared to inulin.

After an initial rapid modulation in overall gut microbiota composition and a decrease in gut bacterial richness within six hours, distinct differences between fiber products started to emerge between 6 to 24 h when most of the gas and total SCFAs were produced. Inulin resulted in the largest pH decrease and highest gas production, with propionate production surpassing that from dried chicory root cubes and powder at 24 h. While acetate and total SCFA production did not differ, both dried chicory root products produced significantly more butyrate than inulin. This was paralleled by significantly larger lactate production (up to 6 h) and consumption (from 6 h onwards) for dried chicory root cubes compared to hardly any consumption between 6 to 24 h for inulin, and little changes for dried chicory root powder. Comparing dried chicory root particle sizes, gut bacterial richness decreased less rapidly for powder than cubes. This may be due to the presence of more readily available substrate as the plant cell matrix in powder is more damaged. Consequently, more fibers are exposed (intracellular inulin alongside pectin and hemi-/cellulose cell wall fibers) and more surface area is created for bacterial adherence compared to the cubes, where bacteria have to diffuse between the plant cells to break them down from the outside.

Between 24 and 48 h, the overall gut microbiota community composition hardly changed, but we observed remarkable distinctions in butyrate production and relative abundances of individual taxa. Gut bacteria richness increased again at 48 h for dried chicory root cubes and powder but not inulin. This coincided with higher levels of the pectin-degraders *Monoglobus* spp. and bacteria from the *Eubacterium eligens* group, as well as the butyrate-producing *Roseburia* spp. in both the fermentation liquid and mucin-covered beads. The re-increase in gut bacteria richness and appearance of specialist bacteria suggests that the more intact plant cell matrix of dried chicory root results in prolonged fermentation, preventing substrate depletion at a later time point. Interestingly, lactate consumption and butyrate production were also highest between 24 and 48 h, resulting in butyrate being the only SCFA to notably increase during this time period and to reach the highest levels for dried chicory root cubes. Increased butyrate production levels after prolonged fermentation have been previously reported for large wheat bran particles ^[18,21]^. It appears that the three-dimensional organization plays a role herein, as exemplified by alginate-entrapped starch^[60]^ and wheat plant cell walls^[58]^, leading to higher butyrate production than their extracted single fiber alone. Especially larger particle sizes (> 2 mm) often result in higher butyrate and lower acetate proportions^[20]^, which is attributed to reaching a fermentation plateau and allowing more time for lactate/acetate-to-butyrate conversion through cross-feeding^[20]^. Lactate conversion into propionate or butyrate is limited *in vitro* at a more acidic pH (5.5 *vs.* 6.5)^[61]^, but it is debatable whether the slightly lower pH in inulin (5.75) compared to dried chicory root (5.89) fermentations, was physiologically relevant to be the main driver of higher lactate consumption and butyrate production. However, we observed for dried chicory root cubes, the largest lactate production and consumption coincided with the largest butyrate production. This suggests that lactate-to-butyrate conversion could play an essential role in the breakdown of the dried chicory root cubes.

Finally, we observed a lower increase in BCFAs and ammonium for inulin compared to the dried chicory root products and control. This may be attributed to the significantly greater pH decrease for inulin, as the formation of these protein fermentation compounds is known to be less favored at lower pH^[11]^. In the *in vivo* situation, the production of BCFAs is linked to the availability of fiber substrate in the proximal versus distal colon, and inulin, due to its simple structure, is believed to be fermented more proximally^[11,12]^. Consequently, BCFA formation may still occur *in vivo* in the distal colon due to substrate depletion from inulin but not from dried chicory root as a consequence of its slower breakdown.

Butyrate produced by gut bacterial fiber fermentation can be used by colonocytes as an energy source and thereby potentially strengthen the colonic epithelium. Studying this interaction relies on the use of cultivated cell-line models (possibly combined with mimicking mucus secretion) or the removal of *in vivo* colonic tissues (yet losing the protective mucus layer). Previous studies using colonic biopsies revealed acute distinct effects of fiber on permeability in biopsies of healthy and/or diseased donors^[62,63]^, but also considerable heterogeneity in the individual responses^[63]^. Here, we applied a similar acute model to study the interaction between the individuals’ gut microbial fiber breakdown products and their human colonic epithelium. To model this, we investigated whether previous exposure to dried chicory root fermentation supernatants could acutely counteract or diminish stressor-induced impairments in the gut barrier function similar to a low-grade inflammatory state. We did not observe an acute, uniform difference between human biopsies stressed with SDC alone and those that were previously exposed to the fermentation supernatant, despite a less rapid decrease in TER over time in the latter. Based on the measured SCFA concentration in fermentation supernatants and dilution in the Ussing chamber, we estimated butyrate concentration to be 0.25 mM. Previous studies using sodium butyrate at a concentration of 5 and 25 mM (factor 20 to 100 higher, representing physiological concentrations) also failed to demonstrate a protective acute effect against stressor-induced impairments using the mast cell degranulator Compound 48/80 (C48/80) in healthy donors’ biopsies^[40]^. It is possible that the acute exposure in this model was too short to positively modulate gut barrier integrity. Butyrate has a considerable history of well-reported improvements on human colonic function *in vivo*^[64]^ and two-week *in vivo* exposure to butyrate using direct delivery by enemas has been shown to reduce oxidative stress in the colonic mucosa^[64]^.

In line with an expected heterogeneity in the individual responses, we observed donor-specific differences, which became apparent in unstressed biopsies. Notably, for two donors (donors 2 and 4), exposure to the dried chicory root fermentation supernatant did not compromise, but maintained overall gut integrity measured by TER compared to the changes observed in control biopsies (%TER change). These donors also had the highest final relative levels of *Bifidobacterium* spp. at 48 h of fermentation. Bifidobacteria can positively impact gut epithelium proliferation via direct interaction of their tight adherence pili with colonic cells in mice^[65]^. Moreover, bifidobacteria are known to strengthen the gut mucosa (despite being absent in this acute model) via the proposed interaction of the neurotransmitter GABA (γ-aminobutyric acid) and the SCFA acetate with goblet cells^[66]^. Therefore, it is possible that a beneficial effect of dried chicory root fermentation on gut barrier integrity in such an acute model may become more apparent by using donors who previously consumed the dried chicory root product. Furthermore, studying a larger number of donors would enhance our understanding of the overall significance of the present observation.

Understanding time-dependent digestive changes in food products relies on *in vitro* systems, with static (batch) incubations that are commonly used due to cost-effectiveness. However, these systems only model and do not represent human digestive processes^[67]^. Here, we used the standardized upper gastrointestinal *in vitro* INFOGEST model^[30,31]^ tailored (as recommended) to our high dietary fiber product indigestible to human endogenous enzymes. The observed minimal structural changes supported the simplified incubations under anoxic, sterile conditions before *in vitro* fermentations. Additionally, we used simple fecal batch *in vitro* fermentations, which are common for individual and pooled microbiota response assessments. However, these batch fermentations are prone to known model-induced shifts in bacterial taxa^[68]^, limiting *in vivo* generalizability. Particularly, we noticed an increase in the common fast-growing, aerotolerant sugar-fermenter *Escherichia-Shigella* spp. in the control fermentations. It remains to be assessed how these higher relative levels in the control would reflect in absolute numbers as we did not measure bacterial load. Various other studies have observed this Enterobacteriaceae overgrowth^[69-74]^, which reflects the model’s limitations including the nature of the inoculum (low bacterial cell numbers favoring *Escherichia-Shigella* spp.)^[69]^ and potential residual or re-entering oxygen. It is unlikely that the 4% mono-/disaccharides naturally present in the dried chicory root products substantially contributed to fermentation, although they might be absorbed *in vivo* in the small intestine. Moreover, we used two *in vitro* fermentation systems that differed in their set-up. The Ussing chamber fermentation was used to produce fermentation supernatants and contained a higher amount of fecal inoculum (1:5 *vs.* 1:13) than the single donor experiment. This exposed more bacterial cells to the same amount of fiber substrate, reaching the fermentation plateau faster, as observed from a higher amount and rate of butyrate production. However, in spite of these limitations and different model configurations, remarkably similar responses were obtained across donors, resulting in increased levels of microbial butyrate producers and butyrate levels, which is in line with previous *in vivo* results^[27]^. This demonstrates the donors’ individuality in gut microbiota composition and, at the same time, the potential of dried chicory root to increase butyrate production across gut bacterial communities from different donors.

In all donors, lactate increased at 6 h and was subsequently consumed throughout the fermentation coinciding with butyrate production. For the low bifidobacteria donor, lactate originated possibly by the action of *Streptococcus* spp.^[75]^, while for the donors, in the Ussing chamber fermentations, *Bifidobacterium* spp. increased by seven-fold and coincided with lactic acid production. Previously, we showed that dried chicory root had a strong bifidogenic effect, increasing *Bifidobacterium* spp. by four-fold *in vivo*^[27]^. For all donors, we also observed an increase in butyrate and propionate production with significant changes in the relative levels of *Roseburia* spp. (single donor) or *Butyricicoccus* spp., both known butyrate producers^[75,76]^. However, neither *Roseburia* spp. nor *Butyricicoccus* spp. reportedly use lactate for butyrate production^[75]^. We hypothesize that the ongoing activity of known lactate-utilizing bacteria from the *Eubacterium hallii* group (renamed to *Anaerobutyricum* spp.)^[49,77-79]^ contributed to butyrate formation from lactate despite their relative levels not continuously increasing. The same has been observed in synthetic communities where the gene expression of the lactate-to-butyrate pathway was highly increased rather than their cell numbers^[78]^. Our previous *in vivo* results demonstrated that the increase in *Bifidobacterium* spp. was concomitant with a three-fold increase in the well-known butyrate-producing *Anaerostipes* spp. having the same lactate-to-butyrate pathway as *Anaerobutyricum* spp.^[75,79]^. Then, we demonstrated using a synthetic community that representative members with the canonical functionality of these genera formed a trophic chain yielding butyrate from dried chicory root^[27]^. Hence, it is possible that similar trophic chains involving lactate/acetate producers and butyrate producers were formed here, too, but represent donor and model-specific cross-feeding networks.

In this study, we demonstrate how the presence and intactness of the plant cell matrix in dried chicory root affect the bacterial breakdown of its dietary fiber, contrasting it with isolated inulin. Dried chicory root, particularly in the form of cubes, resulted in lower gas and more butyrate production compared to isolated inulin, although cumulating into similar final total levels of SCFAs. Lower gas production has been postulated to be a beneficial outcome *in vivo* especially in fiber-related therapies for irritable bowel syndrome^[12]^. The observed butyrate production throughout the later stage of fermentation, together with the detection of a re-increase in pectin-degraders, may translate *in vivo* into a prolonged fermentation, during which chicory root fibers are transported into the distal colon where butyrate production could benefit gut health. This would also explain the high levels of butyrate and other SCFAs in the fecal samples of our previous dried chicory root randomized-controlled *in vivo* trial. We were not able to demonstrate a uniformly strengthening effect of butyrate containing fermentation supernatants in the Ussing chamber using biopsies stressed with SDC, but observed donor-specific effects for overall gut integrity. However, high production of butyrate from the dried chicory root cubes could still benefit gut barrier integrity *in vivo*. In conclusion, dried chicory root is an intrinsic fiber product containing high amounts of inulin that remain encapsulated within its plant cell matrix in the upper gastrointestinal tract - this affects its breakdown kinetics by the human gut microbiota rationalizing a more distal fermentation and production of butyrate benefitting human health *in vivo.*

## DECLARATIONS

### Availability of data and materials

Data are available in the Supplementary Materials, and 16S rRNA gene amplicon sequences are submitted to the European Nucleotide Archive (ENA) under accession numbers PRJEB74436 and PRJEB74437.

### Copyright

© The Author(s) 2024.
