# Supplementary material for: Analysis of the fermentation kinetics and gut microbiota modulatory effect of dried chicory root reveals the impact of the plant-cell matrix rationalizing its conversion in the distal colon
Source: Microbiome Res Rep. 2024 Apr 26;3(3):28. doi: 10.20517/mrr.2024.04 (PMC11485554; doi:10.20517/mrr.2024.04)
Supplement: Supplementary file 1 [file mrr-3-3-28-SupplementaryMaterials.pdf]

Puhlmann *et al. Microbiome Res Rep* 2024;3:28
DOI: 10.20517/mrr.2024.04

Microbiome Research Reports

Supplementary Materials

Analysis of the fermentation kinetics and gut microbiota modulatory effect of dried chicory root reveals the impact of the plant-cell matrix rationalizing its conversion in the distal colon

Marie-Luise Puhlmann[1,2], Ember van de Rakt[2], Evangelia N. Kerezoudi[3,4], Ignacio Rangel[3], Robert J. Brummer[3], Hauke Smidt[1], Frederik S. Kaper[5], Willem M. de Vos[1,6]

[1]Laboratory of Microbiology, Wageningen University & Research, Wageningen 6708 WE, the Netherlands.

[2]Division of Human Nutrition and Health, Wageningen University & Research, Wageningen 6708 WE, the Netherlands.

[3]Nutrition-Gut-Brain Interactions Research Centre, School of Medical Sciences, Faculty of Medicine and Health, Örebro University, Örebro 70182, Sweden.

[4]Department of Nutrition and Dietetics, Harokopio University, Athens 17671, Greece.

[5]WholeFiber BV, Emmeloord 8300 BA, the Netherlands.

[6]Human Microbiome Research Program, Faculty of Medicine, University of Helsinki, Helsinki 00014, Finland.

Correspondence to: Marie-Luise Puhlmann, Laboratory of Microbiology, Wageningen University & Research, Stippeneng 4, Wageningen 6708 WE, the Netherlands. E-mail: marie-luise.puhlmann@wur.nl

The aim of the study was to assess the effect of the plant cell matrix in dried chicory root on its upper gastrointestinal (GI), lower gut fermentation and the potential effect thereof on gut barrier integrity by means of *in vitro* and *ex vivo* models. Here we present first a more detailed description of materials and methods. This is followed by additional data on micro- and macroscopic changes as well as pectin and inulin release during upper gastro-intestinal *in vitro* digestion. Subsequently we show the additional analyses of the gut microbiota composition during lower gastro-intestinal fermentation of dried chicory root powder and cubes versus inulin. Here we include visualizations of β-diversity and the details on the statistical analysis of changes in α-diversity, common genera and fermentation outcomes (pH, gas, short-chain fatty acids (SCFAs), branched-chain fatty acids (BCFAs), lactate and ammonium). Finally, for the *ex vivo* assessment of the effect of fermentation supernatants on human colonic biopsies in an Ussing chamber experiment, we summarize additional details. These include gut microbiota composition changes during the fermentation to produced fermentation supernatants as well as changes in fermentation outcomes. Furthermore, we provide more information on electrophysiological and gut permeability markers assessed during the Ussing experiment.

Supplementary Materials and Methods

Dried chicory root powder

To study the effect of particle size, dried chicory root cubes were ground for upper gastrointestinal tract digestion using a cryogenic grinder (60 seconds, SPEX SamplePrep, 6875D Freezer/Mill®) and a 40 mesh sieve (Retsch, Waalwijk, The Netherlands) to obtain particles of <0.5 mm and for lower GI tract fermentation dried chicory root powder (mean particle size <0.5 mm) was provided by WholeFiber BV.

Adapted Infogest model

To study upper gastrointestinal changes we used the INFOGEST model [1,2]. The INFOGEST protocol is an international consensus providing standardized guidelines for the use of electrolyte and enzyme concentrations as well as duration and pH to simulate the upper gastrointestinal tract. In short, the food of interest is incubated for 2 min at pH 7 to mimic the oral phase in simulated salivary fluid, followed by an incubation for 2 hours at pH 3 of the oral bolus in 1:1 simulated gastric fluids and a small intestinal phase for 2 hours at pH 7 at a ratio of 1:1 in small intestinal fluids. All chemicals were obtained from Sigma-Aldrich (Zwijndrecht, The Netherlands), except for HCl (37%) and ethanol (96%) from VWR (Amsterdam, The Netherlands). Digestive fluids were prepared according to the INFOGEST protocol, as well as pepsin stocks and a pancreatin stock based on trypsin activity [1,2]. The protocol was further adapted to the research question as recommended by the INFOGEST protocol. 1) For the oral phase an additional 1:1 (w/v) dilution of the product was done in water prior to the addition of simulated salivary fluid, to mimic the intake of the product with a liquid/semi-liquid food and achieve a consistency as recommended by INFOGEST. To this mix simulated salivary fluid was added at a ratio of 1:1 based on the dry weight of the product. Salivary amylase was omitted due to the absence of glucose-polymers in the product. 2) For the gastric phase the pH was lowered in two steps to mimic potentially harsher conditions of gastric pH close to the fasting state or end of the gastric digestion (pH 2). The product was first incubated for 1 hour at pH 3 followed by lowering to pH 2 and further incubation for another hour. No phospholipids were used due to the absence of fat in the product. 3) For the small intestinal phase pancreatin was chosen over individual enzymes to assess the overall effect of the small intestinal phase

rather than focusing solely on the specific enzymatic activity targeting a single compound in the plant cell matrix. Additionally, bile acids were omitted due to the absence of fat in the product. For each particle size (powder and cubes) 4 g were used as starting material. The experiment was executed in 50 mL Falcon tubes attached to a rotary wheel set to 20 rpm (Stuart, Tube Rotator SB3) and incubated in an oven (Thermo Scientific Heraeus®, UB 12) set to keep the temperature of the digestive liquid at 37 °C. Separate experiments were executed in triplicate to sample from each phase as the physical structure of the product did not allow for continuous sampling. Samples were taken at the end of the oral phase (2 min) and after 30, 60 and 120 min of both the gastric and the small intestinal phases. After each respective phase the experiment was stopped by separating the liquid digesta from the solid part using a sieve. Liquid digesta were heat treated (5 min at 100 °C) to inactivate remaining enzymatic activity and snap frozen in liquid nitrogen and solid parts were fixated in 70% ethanol for immediate image processing.

Scanning Electron and Light Microscopy

To assess possible changes in the plant cellular matrix of the chicory root, samples of the dried chicory root (rehydrated cubes before start of the oral phase and samples at the end of the gastric and small intestinal phase) and for comparison fresh chicory root were visualized using scanning electron microscopy (SEM) and light microscopy. For SEM fresh chicory root was cut into piece of similar size as the dried chicory root. Digested dried chicory samples were prepared on the morning of the analysis. Samples were first dehydrated in 70% ethanol and then cut into smaller pieces to visualize the inner structure of the plant cell matrix. Subsequently samples were dried in 100% ethanol with CO_2 using a Leica EM CPD300 critical point dryer (Leica Camera, Germany), mounted onto aluminum stubs and coated with a layer of 12 nm tungsten. Samples were visualized using a FEI Magellan 400 Scanning Electron Microscope (Field Electron and Ion Company, USA). For light microscopy, fresh and rehydrated as well as digested chicory root were first cut into thin slices using a razor blade and treated with a drop of 100% ethanol or methanol to precipitate inulin dissolved inside the vacuoles. Ruthium red (0.05% w/v) in distilled water) was added to increase the contrast between plant cell wall fibers and intracellular inulin. Then, the plant cell structure was visualized using a Olympus BX41 light microscope equipped with a

Olympus SC30 Color Camera and cellSens software (Olympus, Hoofddorp, The Netherlands).

Pectin analysis as a measure of pectin leakage

To estimate possible pectin leakage from the plant cell matrix, uronic acid concentration as a measure of pectin content was determined in the liquid digesta obtained from the gastric and small intestinal phase. Samples were first centrifuged for 5 min at 16100 x *g* and the respective supernatants were diluted 12.5 x for the gastric phase and 7.5 x for the small intestinal phase. Uronic acid analysis was performed with an automated colorimetric *m*-hydroxydiphenyl assay [3] using a Skalar San++ continuous flow analyzer (Skalar Holding B.V., Bread, The Netherlands). Neglecting other pectic sugars, the amount of pectin was then estimated based on the detected uronic acid concentration and expressed as percentage of an expected total uronic acid content (5 % w/w) in chicory root as determined according to Ramasamy et al. [4].

Inulin analysis as a measure of inulin leakage

To assess potential differences between inulin from dried chicory root powder versus cubes leaking into digestive fluids in the upper GI tract we used High Performance Anion Exchange Chromatography (HPAEC). Liquid digesta were first centrifuged for 5 min at 16100 x *g* and the respective supernatants were diluted 100 x for samples from the gastric phase and 50x for the samples from the small intestinal phase. Samples were then injected into a Dionex ICS-5000+ Ion Chromatography System (Dionex, Sunnyvale, CA, USA) equipped with a Dionex CarboPac PA-1 column (250 mm × 2 mm ID), a Carbopac PA-1 guard column (25 mm × 2 mm ID) and a ISC5000 ED detector (Dionex) in the pulsed amperometric detector (PAD) mode and measured according to established methods [5]. Chromatograms revealing fructan-molecules of different chain lengths (degree of polymerization, DP) of inulin including the mono/disaccharides and fructan-oligomers (DP2 – 10) as well as longer-chain fructan-polymers (>DP10) present in the digesta were analyzed using Chromeleon™ Chromatography Data System software version 7 (ThermoFischer Scientific).

Batch incubation and fermentation model for assessing particle size effects

A fecal *in vitro* batch fermentation experiment was performed at ProDigest (Gent, Belgium). A 26-year-old female donor was selected, meeting the criteria of a normal BMI (18.5 – 24.9 kg/m²), with no history of diseases related to disturbances in the gut microbiome (e.g., irritable bowel syndrome, Parkinson's, or diabetes mellitus), and no antibiotic usage within four months prior to fecal donation.All chemicals were purchased from Sigma Aldrich (Overijse, Belgium). A simplified upper GI tract incubation was performed, by incubating 350 mg of dried chicory root cubes, powder or inulin versus control (no product) in 20 mL sterile water for 2 h under shaking (90 rpm) at 37°C in 100mL Schott bottles. To this 43 mL of buffered minimal medium was added containing 7.6 g/L K_2HPO_4, 23.9 g/L KH_2PO_4, 2.9 g/L $NaHCO_3$, 2.9 g/L yeast extract, 2.9 g/L peptone, 0.7 g/L cysteine and 2.9 mL/L Tween80 as well as five mucin-covered beads prepared as specified elsewhere [6]. The bottles were sealed and flushed with nitrogen. For the inoculum, a fecal slurry was prepared by mixing fresh feces at 1:13 (m/v) in an anaerobic phosphate buffer containing 8.8 g/L K_2HPO_4, 6.8 g/L KH_2PO_4, 0.1 g/L sodium thioglycolate, 0.015 g/L sodium dithionite, homogenizing the mix for 10 min in a lab blender (Bag Mixer 400, Interscience, Louvain-La-Neuve, Belgium) and removing big particle by centrifugation at 500 x *g* for 2 min. All bottles, were inoculated with 7 mL fecal slurry and incubated at 37 °C at 90 rpm for 48 h. Experiments were performed in triplicate and samples were taken at baseline and after 6, 24 and 48 h and frozen immediately for bacterial metabolite analysis. For DNA extraction 1 mL of sample was centrifuged at 5000 x g for 10 min and separated into pellet and supernatant before storing at -80 °C. Metabolic microbial activity was assessed by measuring in the fermentation samples pH using a Senseline F410 pH meter (ProSense, Oosterhout, The Netherlands), and fecal (un-/branched) short-chain fatty acids (SCFAs) as described elsewhere [7] as well as lactate and ammonia [8]. Gas pressure in the fermentation bottles was measured using a hand-held pressure indicator (CPH620, Wika, Echt, The Netherlands) and recorded before and after sampling at each timepoint. The change in gas pressure between timepoints was calculated based on the respective timepoint's gas pressure levels before sampling minus the gas pressure levels of the previous timepoint after sampling.

Human donors for Ussing chamber

Four healthy adult subjects with a mean age of 42 years (range 27-62 years) were recruited to donate fecal material and colonic biopsies and all subjects had signed an informed consent. The study was approved by the Research Ethics Committee of Örebro University (Dnr 2022-01814-02) and was registered at ClinicalTrials.gov (NCT05421793) and conducted in accordance with the Helsinki Declaration. Subjects did not consume any pre- or probiotics during the previous month, no antibiotics, laxatives, anti-diarrheal/-cholinergic drugs during the previous three months, and no Non Steroidal Anti Inflammatory Drugs during the past two months. Furthermore, they had no gastrointestinal disorder, previous surgery, psychiatric disorder expected to affect study outcomes or obesity. Subjects provided a fecal sample in the same or preceding week of biopsy collection.

Batch fermentation model for Ussing chamber

All chemicals were purchased from Sigma Aldrich (Stockholm Sweden) unless stated otherwise. A batch fermentation experiment was performed for each donor to produce fermentation supernatants from dried chicory root cubes. Fecal slurries were prepared from fresh feces (within 2 h after feces collection) by mixing 20 g of feces in 100 mL anaerobic phosphate buffer (Phosphate Buffered Saline containing 0.14 M NaCl, 0.0027 KCl and 0.010 M phosphate buffer pH 7.4, Medicago, Uppsala Sweden) with 0.5 g/L L-cysteine, homogenizing for 2 min by hand in a Stomacher® Circulator Strainer Bag with a pore size of 0.5 mm (Seward, Worthing, UK). Of this fecal slurry 10 mL were transferred into sterile 500 mL Schott bottles containing 90 mL of buffered colonic background medium containing 2.8 g/L K_2HPO_4, 15.4 g/L KH_2PO_4, 2.0 g/L $NaHCO_3$, 2.0 g/L yeast extract (Oxoid, Malmö, Sweden), 2.0 g/L peptone water (Oxoid, Malmö, Sweden), 0.1 g/L NaCl, 0.01 g/L $MgSO_4.7H_2O$, 0.01 g/L $CaCl_2.6H_2O$, 0.05 g/L hemin, 10 µL/L vitamin K1, 0.5 g/L bile salts, 1.0 g/L cysteine and 2.0 mL/L Tween80. Prior to inoculation, 5 g/L of dried chicory root cubes were incubated for approximately 2 h in the medium in the bottles, which were flushed with nitrogen and placed in a water bath for incubation at 37 °C. Per donor a single fermentation of dried chicory root cubes and a single negative control (only inoculum) fermentation were executed. Samples were taken at baseline, 6, 24 and 48h (except for donor 1 for which samples

were taken at 42h) and stored at -80 °C. Fermentation supernatants were produced by centrifuging 1 mL of sample at 31,514 x *g* for 10 min at 4 °C. Supernatants were aliquoted for SCFA measurements and Ussing chamber experiments and the pellets were stored for DNA extraction at -80 °C. Changes in pH were assessed in fermentation supernatant using a FiveGo pH meter F2 equipped with a Sensor InLab® micro Pro pH probe (Mettler Toledo, Stockholm, Sweden) and gas pressure was recorded using the Automatic Gas Flow Measuring System Gas Endeavour (BPC Instruments, Lund, Sweden). Short-chain fatty acids were analyzed at Wageningen University using high-performance liquid chromatography (HPLC). First a Carrez clarification was performed to remove proteins and carbohydrates in the fermentation supernatant that could interfere with the HPLC measurement. For this first two parts of fermentation supernatant were mixed with one part of Carrez reagent I (0.1 M $K_4Fe(CN)_6.3H_20$) followed by adding one part of Carrez reagent II (0.2 M $ZnSO_4.7H_2O$) and centrifugation for 5 min at 21,000 x *g*. The clear supernatant was then acidified by mixing 1:1 with 0.2 N H_2SO_4 containing 10 mM DMSO as internal standard. A standard of acetic, propionic, butyric, lactic and formic acid was prepared at 1 mM, 5 mM, 10 mM, 20 mM and 30 mM. SCFA were quantified on a Shimadzu LC_2030C equipped with a refractive index detector and a Hi-Plex H column (Shimadzu Europe Company, ‘s-Hertogenbosch, The Netherlands). Ten µL of the sample was injected at an oven temperature of 45 °C with a flow rate of 1.00 mL/min using 0.01 N H_2SO_4 as eluent. Obtained chromatograms were further processed using Chromeleon™ Chromatography Data System software version 7 (ThermoFischer Scientific).

Ussing chamber experiment and gut permeability analysis

Four Ussing chamber experiments were conducted using colonic biopsies, with each colonic biopsy paired to the fermentation supernatant derived from its respective donor’s fecal material. Collection of colonic biopsies as well as set-up and execution of the Ussing chamber experiment were performed according to previously described methods [9]. In short, colonic biopsies were collected by endoscopic procedure from unprepared sigmoid colon after an overnight fast, immediately put in ice-cold oxygenated Krebs-Ringer bicarbonate transport buffer hereafter called Krebs buffer) containing 116 mM NaCl, 1.27 mM $CaCl_2$, 3.65 mM KCl, 1.37 mM KH_2PO_4, 23 mM $NaHCO_3$, 1.20 mM $MgCl_2$ at pH of 7.2 (Merck, Solna, Sweden) and brought to the

facility within 10 min. Biopsies were mounted into an Ussing chamber of 1.5 mL and each condition was run in duplicate. Throughout the whole experiment the Ussing chambers were held at 37 °C, continuously oxygenated and electrophysiological measurements were taken every 30 s (transepithelial electrical resistance (TER), potential difference (PD), short-circuit current (Isc)). Biopsies with low chance of viability (among which PD > 0.5) were excluded from the experiments. Krebs experiment buffer (containing additional 5.7 mM sodium pyruvate and 5.7 mM sodium glutamate) with 0.01 M glucose was used for the serosal side and 0.01 M mannitol was used for the mucosal side of the biopsies. After a 25 min adaption period cold Krebs buffer was replaced with warm Krebs buffer, which was again replaced after another 10 min. Then baseline samples were taken from the serosal side for permeability assessment. After this 150 µL of diluted fermentation supernatant (2% (v/v) in mannitol Krebs buffer) was added to the mucosal side of the treatment biopsies, while the control biopsies were kept in only mannitol Krebs buffer. After 20 min incubation, the experiment was started by first adding 300 µL of the stressor (1 mM sodium deoxycholate (SDC) in mannitol-Krebs buffer) followed by the addition 150 µL of a permeability marker solution prepared in mannitol Krebs, which included 2.5 nM Fluorescein isothiocyanate–dextran FD4 (FITC-dextran) as paracellular permeability marker. Samples were taken after 60 and 90 min and paracellular permeability was assessed by fluorometrically measuring passage of FITC-dextran 3000–5000 [9].

Gut microbiota analysis

Gut microbiota composition was determined in both the fermentation pellets of the single donor *in vitro* fermentation experiment as well as fermentations with multiple donors for the Ussing chamber experiment. Bacterial DNA was extracted by resuspending the pellets in 300 µL Stool Transport and Recovery (STAR) buffer (Roche Diagnostics, Almere, The Netherlands), then transferring into sterile screw-cap tubes filled with 0.25 g of 0.1 mm zirconia bead and 3 glass beads of 2.5 mm diameter. Samples were subjected to repeated (3 x 1 min at 5.5 ms) bead-beating in a FastPrep-24™ 5G Instrument (MP Biomedicals, The Netherlands) followed by heating for 15 min at 95 °C at 300 rpm and centrifuging for 5 min at 4 °C at 16100 x *g*. The supernatant was transferred into a sterile Eppendorf tubes, the pellet was resuspended in 200 µL STAR and the cycle of bead-beating, heating and centrifuging was repeated.

Supernatants were pooled and DNA was purified using a customized Maxwell® 16 Tissue LEV Total RNA purification Kit (XAS1220) on the Maxwell® 16 LEV Instrument (Promega, The Netherlands) and eluted in 50 µL nuclease free water (Qiagen, Hilden, Germany). DNA concentration was measured using a Qubit™ dsDNA Quantification BR Assay Kit on a Qubit Fluorometer (ThermoFisher Scientific, The Netherlands) and adjusted to 20 ng/µL with nuclease-free water. The V4 region of the 16S rRNA gene was amplified in duplicate using the barcoded primers 515F (5'-GTGYCAGCMGCCGCGGTAA-3') [10] and 806R (5'-GGACTACNVGGGTWTCTAAT-3') [11]. Each 50 µL PCR reaction contained 10 µL 5x Phusion Green HF buffer (ThermoFisher Scientific), 1 µL 10mM dNT's (Promega, Madison, WI, United States), 0.5 µL Phusion Hot start II DNA 2 U/µL polymerase (ThermoFisher Scientific, The Netherlands), 1 µL of each barcoded forward and reverse 10 µM primer, 1 µL of 20 ng/µL DNA template and 36.5 µL nuclease-free water. The PCR program consisted of an initial 30 s denaturation at 98 °C for 10 min, followed by 25 cycles of 10 s denaturation at 98 °C, 10 s annealing at 50 °C, 10 s elongation at 72 °C, and final extension for 420 s at 72 °C. To verify the presence and size of each PCR product 2 µL were loaded onto a 2.2% agarose gel (Lonza Benelux B.V., Breda, The Netherlands) and run for 5 min at 200 V. The PCR products were pooled, further purified using the CleanPCR kit (CleanNA, Waddinxveen, The Netherlands) and the DNA concentration was again measured using Qubit. A library with an equimolar mix of purified PCR product, negative PCR and DNA extraction controls as well as positive mock communities was prepared and sent for Illumina Hiseq sequencing to Novogene (Novogene, The Netherlands). Raw amplicon sequences were processed using NG-Tax 2.0 with default settings [12] and resulting amplicon sequence variants (ASV) were taxonomically annotated using the SILVA 138.1 database [13].

Upper gastro-intestinal *in vitro* digestion: changes in micro- and macrostructure of dried chicory root and pectin and inulin leakage.

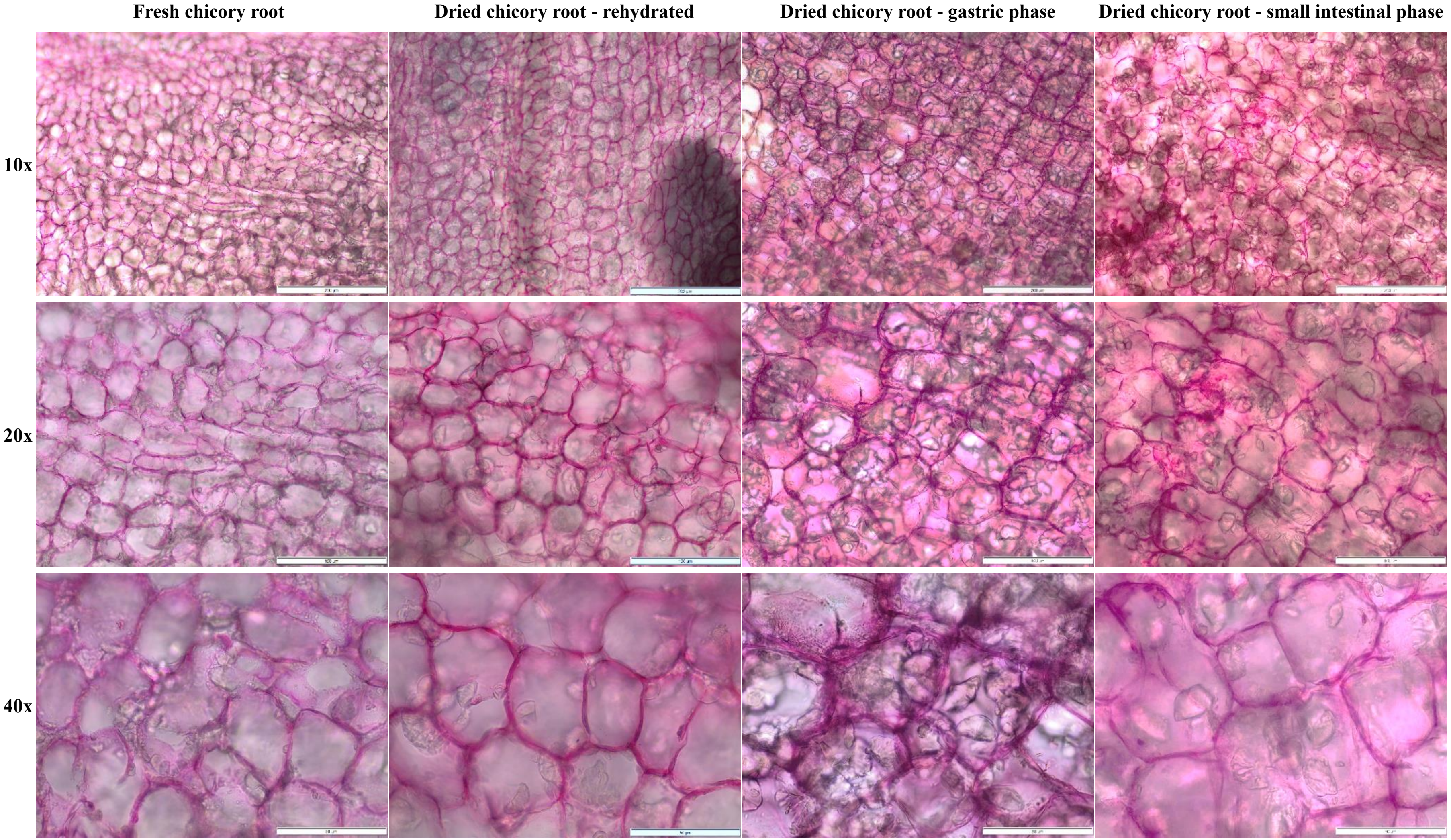

Supplementary Figure 1. Light Microscopy micrographs of the plant cell matrix of fresh and *in vitro* digested dried chicory root cubes before the oral phase (rehydrated), at the end of the gastric (120 min) and small intestinal phase (240 min). Ruthium red (0.05 %w/v) was added to increase the contrast between plant cell and intracellular components. Each picture is shown at three magnification with the scale bar representing 200 µm for 10x magnification, 100 µm for 20x and 50 µm for 40x magnification.

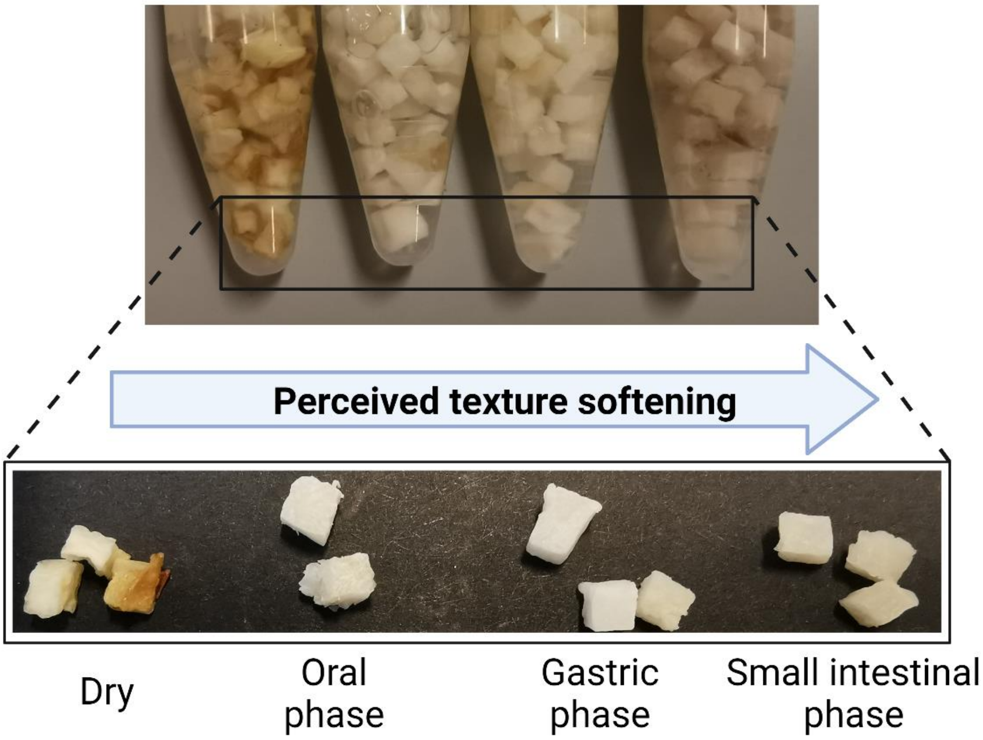

Supplementary Figure 2. Macrostructure of dried chicory root cubes before and during upper gastrointestinal *in vitro* digestion. *Created with Biorender.com*

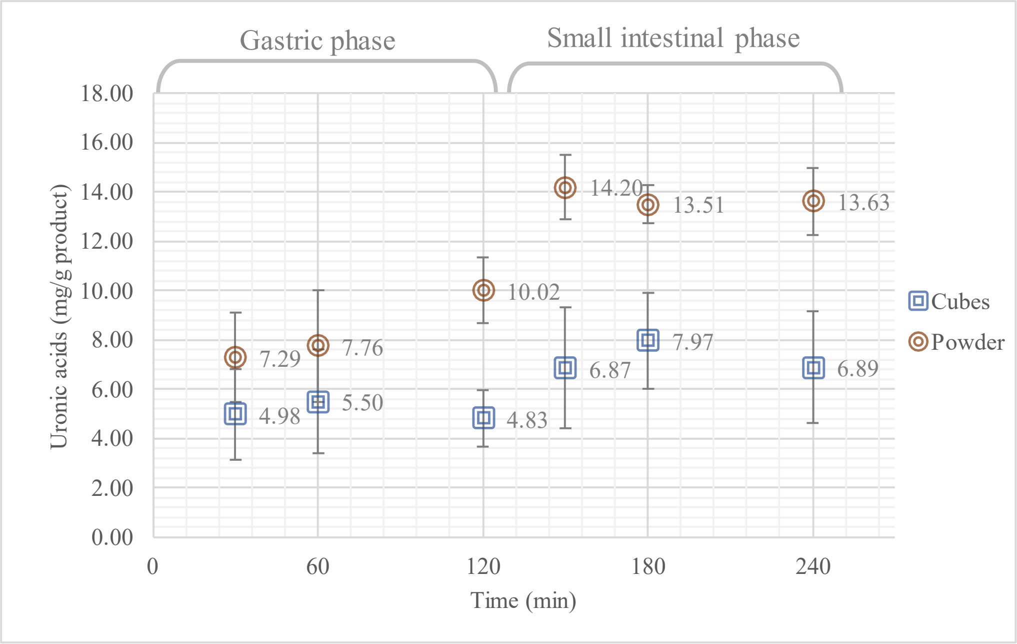

Supplementary Figure 3. Release of uronic acids as a measure of pectin leakage from dried chicory roots cubes (□) and powder (○) during *in vitro* digestion in the gastric phase (at t = 30 min, 60 min and 120 min (end)) and small intestinal phase (30 min at t = 150 min, 60 min at t = 180 min and 60 min at t = 240 min (end)). For each data point the corresponding value is given on its respective right side.

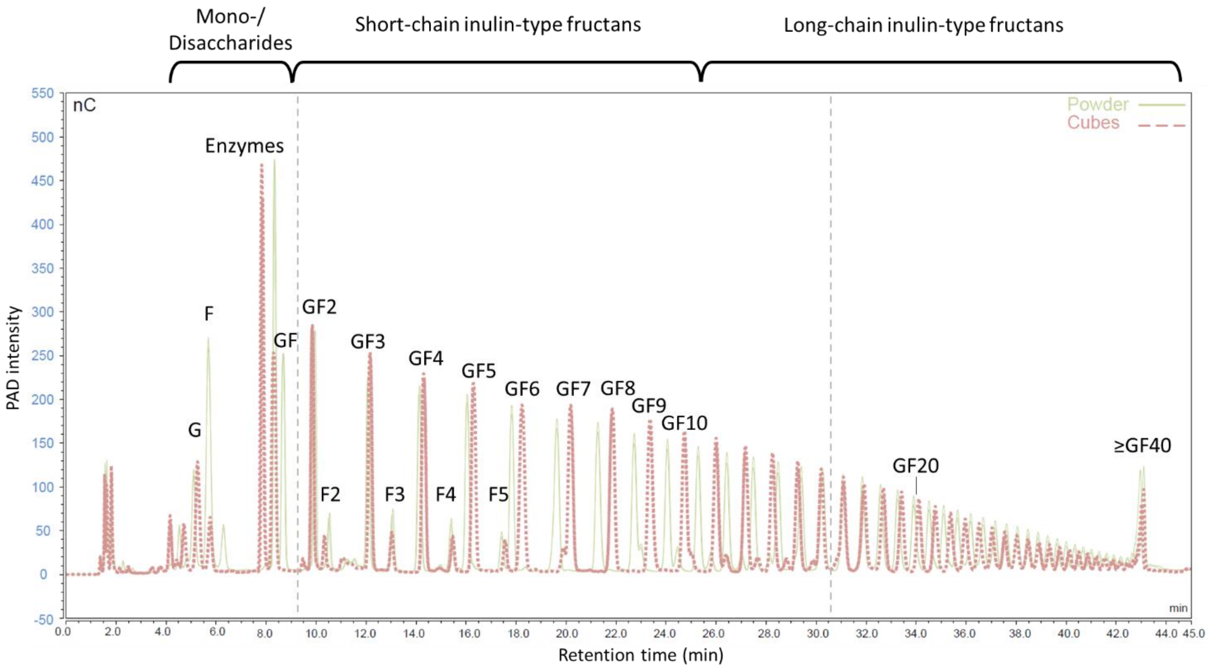

Supplementary Figure 4. High Performance Anion Exchange Chromatography (HPAEC) chromatograms of the liquid digesta retrieved at the end of the upper gastrointestinal *in vitro* digestion (end of the small intestinal phase) for dried chicory root powder (green) compared to cubes (red). Native inulin contains so-called inulin-type fructans, which are fructose-polymers linked by β(2—1)-bonds and an have varying chain lengths up to 60, which are described as degree of polymerization. Native inulin contains oligomers with and without glucose as end unit as well as glucose and fructose monomers and sucrose (di-saccharide of glucose and fructose). Different chain lengths are represented by individual peaks in the chromatogram obtained from the signal of each detected mono-, oligo- and polymer. G: glucose; F: fructose; Enzymes: signal obtained from the added pancreatin (containing digestive enzymes) in the digesta; GF: Sucrose (containing a glucose and a fructose monomer); GF2-GF40: fructose-chains of increasing chain lengths with glucose as end unit (DP is equal to the amount of fructose monomers); F2-5: fructose-chains of increasing chain lengths without glucose as end unit (DP is equal to the amount of fructose monomers).

Native inulin as present in the chicory root plant cells contains fructans with a wide range of molecular size (DP 2 – 60; average of DP 12), that decrease in solubility with increasing chain length [14]. Consequently, we expected that longer fructan-polymers (DP>10) leak less easily from the intact plant cells compared to smaller fructo-oligosaccharides (DP 2-10), but that damage might advance their release. We observed indeed higher amounts of fructan-oligomers as well as some longer-chain fructose polymers for dried chicory root powder, which we hypothesize to represent and relate to the higher content of plant cell damage. Thus, it is likely that dried chicory

root cubes transport inulin mainly encapsulated inside the intact plant cells into the lower (distal) gut.

Lower gastro-intestinal *in vitro* fermentation: changes in overall gut microbiota composition and individual genera over time as well as between genera per timepoint

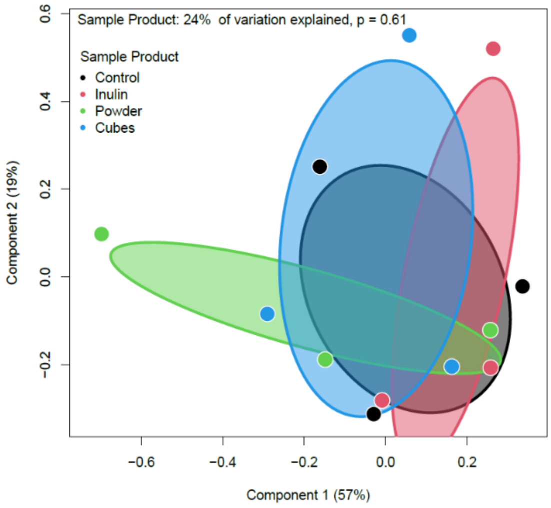

A

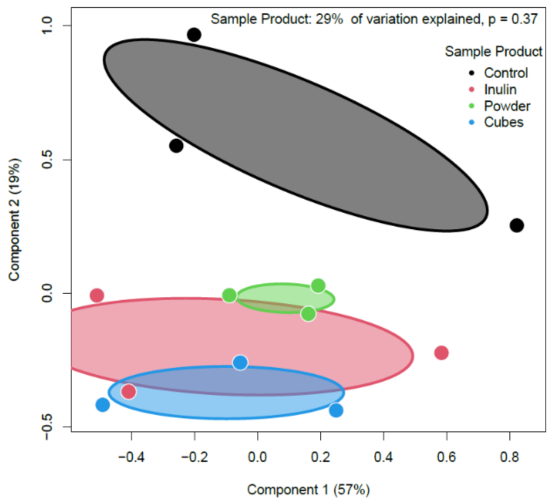

B

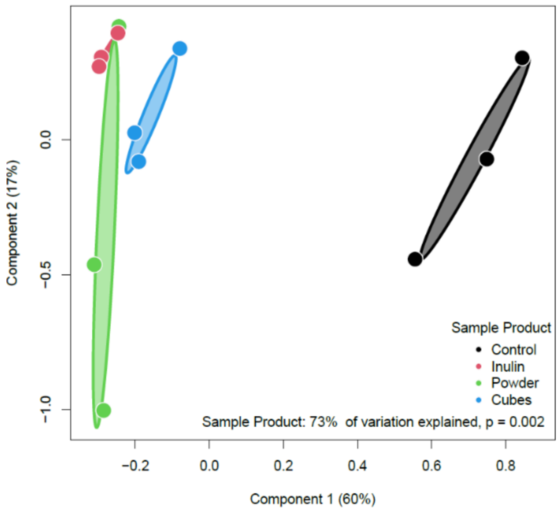

C

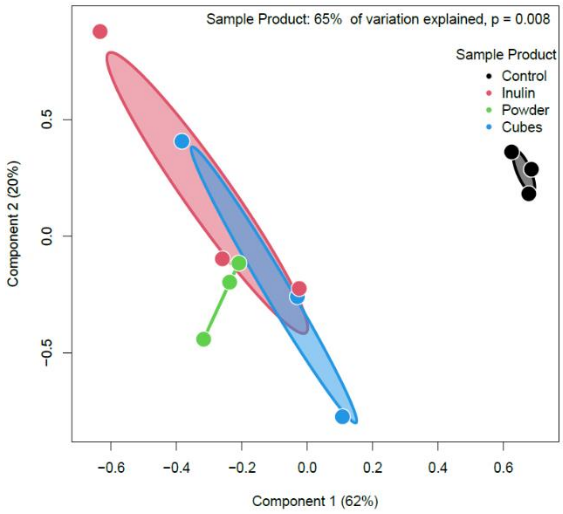

D

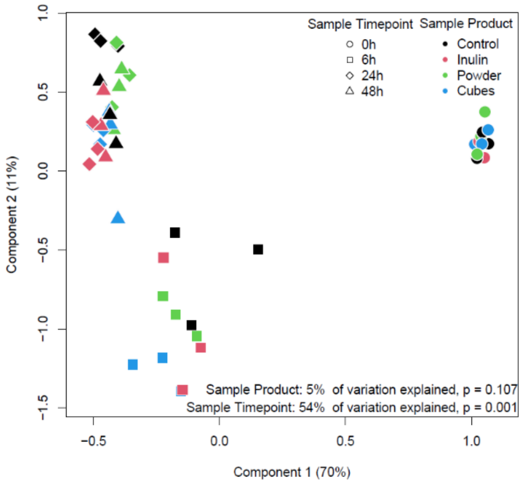

E

Supplementary Figure 5. Principal Coordinate Analysis of gut microbiota composition. A-C: Principal coordinate analysis based on Bray-Curtis dissimilarity to assess differences in overall gut microbiota composition at genus level (β-diversity representing between sample differences) between control fermentation (Control, only inoculum) or *in vitro* fermentation of inulin (Inulin), or dried chicory root powder (Powder) or cubes (Cubes). A: baseline (t = 0 h); B: after 6 h of *in vitro* fermentation; C: after 24 h of in vitro fermentation; and D: after 48 h of *in vitro* fermentation. E: at all time points (0h, 6 h, 24 and 48 h)

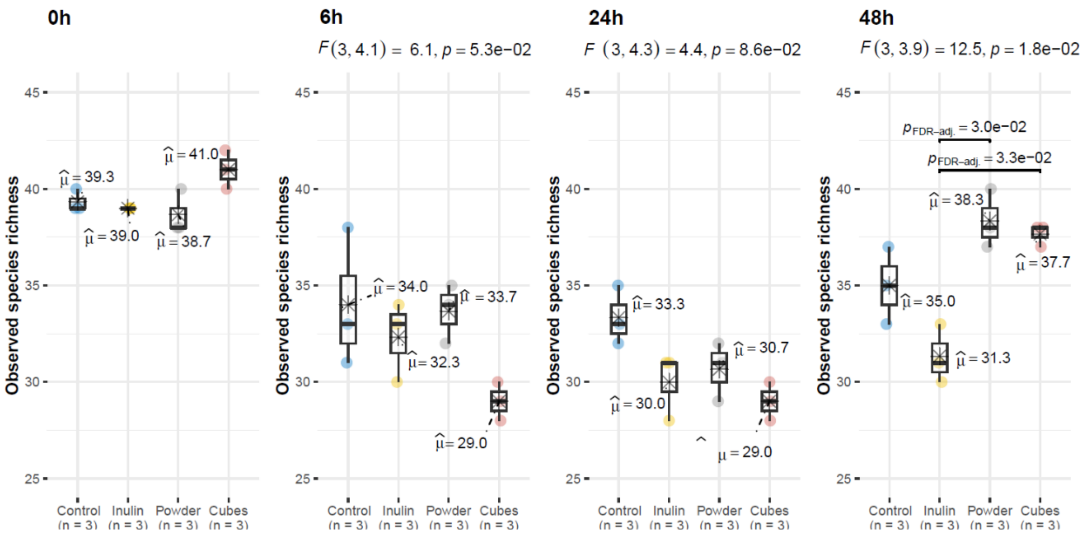

Supplementary figure 6. Alpha-diversity assessed by observed gut bacteria richness (mean richness within each sample) at baseline (t = 0 h), 6 h, 24 h and 48 h *in vitro* fermentation of the control (Control; only inoculum), inulin (Inulin) and dried chicory root powder (Powder) and cubes (Cubes). Statistical details for robust ANOVA (F-statistics and corresponding p-value) are given above the plot. No statistically significantly differences were observed at 0 h, 6 h and 24 h of fermentation between the conditions (pairwise comparison).

Concomitantly with the changes in β-diversity we also observed a rapid change in α-diversity (within sample diversity; Supplementary Figure 6). Overall gut bacteria richness as a measure of α-diversity decreased in all conditions within 6 h and this decrease was largest for dried chicory root cubes. Gut microbiota richness of dried chicory root cubes remained at the lowest level between 6 h and 24 h while inulin and dried chicory root powder further decreased from 6 h to 24 h. However, after 24 h of fermentation gut bacteria richness increased again in dried chicory root powder and cubes reaching statistically significantly higher levels compared to inulin whose gut bacteria richness remained low. In contrast, the richness of the control samples remained decreased throughout the rest of the fermentation.

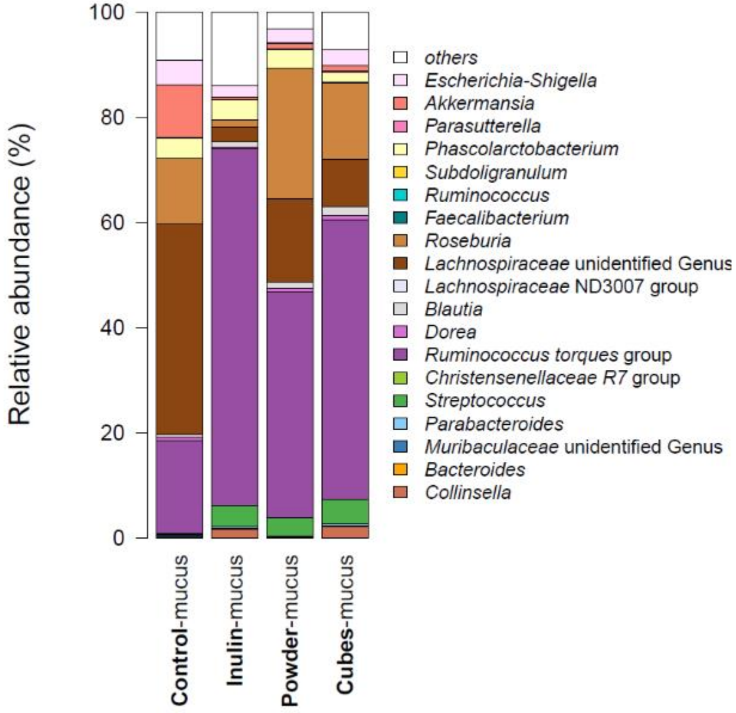

A

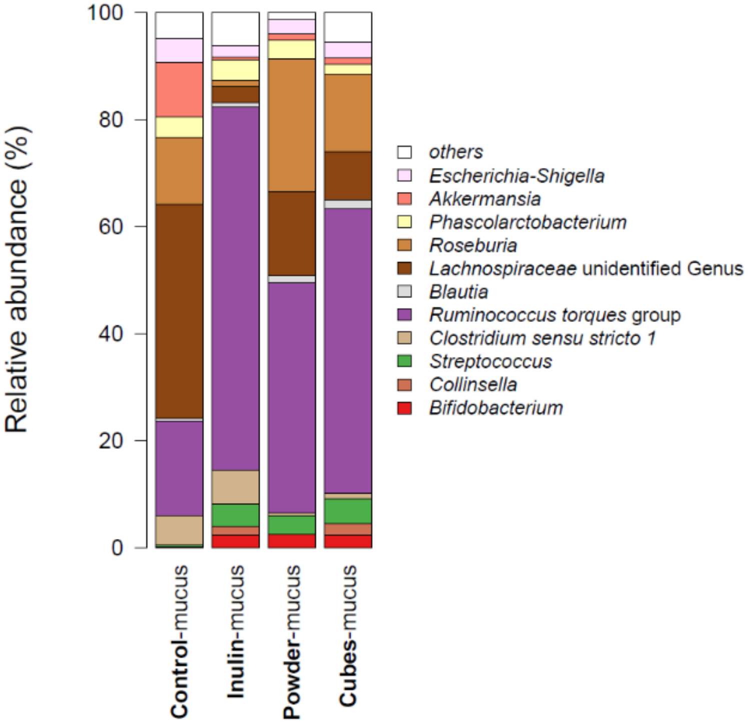

B

Supplementary Figure 7. Mean relative abundances of genera present in mucus recovered from mucin-covered beads for each *in vitro* fermentation (Control, Inulin, dried chicory root Powder or Cubes) at t = 48 h. A: relative abundance of genera present in the mucus samples based on the mean relative abundance of 1% and prevalence of 50% of these taxa in both the fermentation medium and mucin-covered beads. Note that no *Bifidobacterium* spp. is present here. B: relative abundance of genera present in the mucus samples based on the mean relative abundance of 1% and prevalence of 50% of these taxa on only the mucin-covered beads. Note that *Bifidobacterium* spp. is present here.

Supplementary Table 1. Changes in mean relative abundances of common genera (mean relative abundance of at least 1% and mean prevalence of 50% in the whole dataset) during the fermentation of dried chicory root cubes (Cubes) or powder (Powder) vs inulin (Inulin) or negative control (Control; only inoculum) from baseline (t = 0 h) to 6 h, 24 h and 48 h. Respective fold-changes (fold-Δ) over time within each group and the p-values and fdr-corrected q-values are given per taxon for each fermentation.

**Taxon**	**Product**	**relative abundance (%)**				**fold-Δ**			***p-value***			***q-value***		
		**0h**	**6h**	**24h**	**48h**	**0-6 h**	**0-24 h**	**0-48 h**	**0-6 h**	**0-24 h**	**0-48 h**	**0-6 h**	**0-24 h**	**0-48 h**
Adlercreutzia	Control	0.38%	0.76%	0.56%	0.28%	2.02	1.49	0.73	**<0.001**	**0.001**	**0.009**	**<0.001**	**0.001**	**0.012**
	Inulin	0.30%	0.58%	0.47%	0.29%	1.977	1.604	0.975	0.311	0.482	0.971	0.324	0.503	1.000
	Powder	0.35%	0.51%	0.38%	0.44%	1.443	1.081	1.236	0.**025**	0.287	0.075	0.**030**	0.342	0.097
	**Cubes**	0.34%	0.40%	0.52%	0.47%	1.172	1.528	1.361	0.406	0.**026**	0.108	0.448	0.**029**	0.119
Akkermansia	Control	1.01%	3.49%	29.12%	20.40%	3.46	28.83	20.20	**<0.001**	**<0.001**	**<0.001**	**<0.001**	**<0.001**	**<0.001**
	Inulin	1.05%	2.12%	3.66%	4.51%	2.029	3.503	4.312	<0.001	**<0.001**	**<0.001**	**<0.001**	**<0.001**	**<0.001**
	Powder	0.93%	1.83%	5.61%	7.16%	1.972	6.038	7.702	0.011	**<0.001**	**<0.001**	**0.013**	**<0.001**	**<0.001**
	**Cubes**	1.13%	1.51%	6.69%	7.72%	1.338	5.938	6.855	0.219	**<0.001**	**<0.001**	0.251	**<0.001**	**<0.001**
Bacteroides	Control	0.08%	1.25%	1.51%	0.73%	15.53	18.75	8.99	**<0.001**	**<0.001**	**<0.001**	**<0.001**	**<0.001**	**0.001**
	Inulin	0.31%	2.06%	2.62%	2.32%	6.600	8.362	7.408	**0.001**	**<0.001**	**0.001**	**0.001**	**<0.001**	**0.002**
	Powder	0.08%	1.20%	2.47%	2.62%	14.203	29.325	30.993	**<0.001**	**<0.001**	**<0.001**	**<0.001**	**<0.001**	**<0.001**
	**Cubes**	0.13%	1.61%	3.19%	1.82%	12.438	24.587	13.997	**<0.001**	**<0.001**	**<0.001**	**0.001**	**<0.001**	**<0.001**
Blautia	Control	7.47%	2.58%	0.66%	0.73%	0.34	0.09	0.10	**<0.001**	**<0.001**	**<0.001**	**<0.001**	**<0.001**	**<0.001**
	Inulin	7.05%	2.19%	0.82%	3.83%	0.310	0.116	0.544	**<0.001**	**<0.001**	**<0.001**	**<0.001**	**<0.001**	**<0.001**
	Powder	8.50%	2.57%	4.18%	4.42%	0.303	0.491	0.520	**<0.001**	**<0.001**	**<0.001**	**<0.001**	**<0.001**	**<0.001**
	**Cubes**	8.18%	2.22%	3.63%	4.35%	0.272	0.444	0.532	**<0.001**	**<0.001**	**<0.001**	**<0.001**	**<0.001**	**<0.001**
Christensenellaceae R7 group	Control	8.35%	1.57%	0.66%	0.97%	0.19	0.08	0.12	**<0.001**	**<0.001**	**<0.001**	**<0.001**	**<0.001**	**<0.001**
	Inulin	7.32%	1.17%	0.40%	0.43%	0.159	0.054	0.059	**<0.001**	**<0.001**	**<0.001**	**<0.001**	**<0.001**	**<0.001**
	Powder	8.61%	1.59%	0.37%	0.41%	0.185	0.043	0.047	**<0.001**	**<0.001**	**<0.001**	**<0.001**	**<0.001**	**<0.001**
	**Cubes**	8.24%	0.82%	0.39%	0.49%	0.100	0.048	0.060	**<0.001**	**<0.001**	**<0.001**	**<0.001**	**<0.001**	**<0.001**

Taxon	Product	relative abundance (%)				fold-Δ			*p-value*			*q-value*		
		0h	6h	24h	48h	0-6 h	0-24 h	0-48 h	0-6 h	0-24 h	0-48 h	0-6 h	0-24 h	0-48 h
Dorea	Control	0.72%	0.28%	2.96%	3.28%	0.39	4.08	4.52	0.450	**<0.001**	**<0.001**	0.519	**<0.001**	**<0.001**
	Inulin	0.65%	0.25%	10.97%	3.07%	0.388	16.960	4.754	**<0.001**	**<0.001**	**<0.001**	**<0.001**	**<0.001**	**<0.001**
	Powder	0.75%	0.46%	10.72%	1.54%	0.610	14.215	2.047	**<0.001**	**<0.001**	**<0.001**	**<0.001**	**<0.001**	**<0.001**
	**Cubes**	0.82%	0.35%	6.65%	1.43%	0.424	8.120	1.745	0.162	**0.002**	**0.006**	0.192	**0.002**	**0.007**
Enterobacteriaceae unidentified Genus	Control	0.00%	1.04%	0.36%	0.18%	NA	NA	NA	**1.000**	**1.000**	**1.000**	**1.000**	**1.000**	**1.000**
	Inulin	0.00%	0.62%	0.29%	0.49%	NA	NA	NA	**<0.001**	**<0.001**	**<0.001**	**<0.001**	**<0.001**	**<0.001**
	Powder	0.00%	0.28%	0.22%	0.26%	NA	NA	NA	NA	NA	NA	NA	NA	NA
	**Cubes**	0.00%	0.97%	0.54%	0.35%	NA	NA	NA	1.000	1.000	1.000	1.000	1.000	1.000
Erysipelotrichaceae UCG-003	Control	3.50%	1.22%	0.33%	0.21%	0.35	0.10	0.06	**<0.001**	**<0.001**	**<0.001**	**<0.001**	**<0.001**	**<0.001**
	Inulin	3.04%	0.57%	0.25%	0.17%	0.188	0.083	0.054	**<0.001**	**<0.001**	**<0.001**	**<0.001**	**<0.001**	**<0.001**
	Powder	2.88%	0.50%	0.23%	0.19%	0.172	0.079	0.067	**<0.001**	**<0.001**	**<0.001**	**<0.001**	**<0.001**	**<0.001**
	**Cubes**	3.19%	0.44%	0.25%	0.17%	0.139	0.079	0.053	**<0.001**	**<0.001**	**<0.001**	**<0.001**	**<0.001**	**<0.001**
Escherichia-Shigella	Control	0.00%	46.09%	26.06%	33.52%	NA	NA	NA	0.999	0.999	0.999	1.000	1.000	1.000
	Inulin	0.00%	46.23%	31.01%	36.35%	NA	NA	NA	NA	NA	NA	NA	NA	NA
	Powder	0.00%	51.40%	27.27%	32.84%	NA	NA	NA	NA	NA	NA	NA	NA	NA
	**Cubes**	0.00%	53.84%	30.62%	39.08%	NA	NA	NA	NA	NA	NA	NA	NA	NA
Faecalibacterium	Control	15.73%	4.65%	0.60%	0.39%	0.30	0.04	0.02	**<0.001**	**<0.001**	**<0.001**	**<0.001**	**<0.001**	**<0.001**
	Inulin	15.70%	3.83%	0.65%	0.55%	0.244	0.042	0.035	**<0.001**	**<0.001**	**<0.001**	**<0.001**	**<0.001**	**<0.001**
	Powder	18.66%	4.42%	0.56%	0.47%	0.237	0.030	0.025	**<0.001**	**<0.001**	**<0.001**	**<0.001**	**<0.001**	**<0.001**
	**Cubes**	17.21%	3.30%	0.77%	0.61%	0.192	0.045	0.035	**<0.001**	**<0.001**	**<0.001**	**<0.001**	**<0.001**	**<0.001**
Fusicatenibacter	Control	2.24%	0.46%	0.00%	0.00%	0.21	0.00	0.00	**<0.001**	**<0.001**	**<0.001**	**<0.001**	**<0.001**	**<0.001**
	Inulin	1.94%	0.46%	0.05%	0.00%	0.237	0.024	<0.01	**<0.001**	**<0.001**	**<0.001**	**<0.001**	**<0.001**	**<0.001**
	Powder	2.51%	0.38%	0.00%	0.00%	0.153	<0.01	<0.01	**<0.001**	1.000	1.000	<0.001	1.000	1.000
	**Cubes**	2.39%	0.35%	0.00%	0.00%	0.145	<0.01	<0.01	**<0.001**	**<0.001**	**<0.001**	**<0.001**	**<0.001**	**<0.001**

Taxon	Product	relative abundance (%)				fold-Δ			*p-value*			*q-value*		
		0h	6h	24h	48h	0-6 h	0-24 h	0-48 h	0-6 h	0-24 h	0-48 h	0-6 h	0-24 h	0-48 h
Haemophilus	Control	0.00%	0.31%	0.23%	0.08%	NA	NA	NA	**<0.001**	**<0.001**	**<0.001**	**<0.001**	**<0.001**	**<0.001**
	Inulin	0.08%	0.31%	0.43%	0.31%	4.053	5.652	4.011	**0.011**	**0.002**	**0.012**	**0.014**	**0.002**	**0.014**
	Powder	0.00%	0.37%	0.74%	0.48%	NA	NA	NA	1.000	1.000	1.000	1.000	1.000	1.000
	**Cubes**	0.09%	0.28%	0.60%	0.39%	3.124	6.579	4.324	**0.043**	**0.001**	**0.009**	0.053	**0.001**	**0.011**
Lachnoclostridium	Control	0.10%	0.00%	0.94%	0.92%	0.00	9.12	8.89	**<0.001**	**<0.001**	**<0.001**	**<0.001**	**<0.001**	**<0.001**
	Inulin	0.00%	0.00%	0.92%	1.15%	NA	NA	NA	NA	NA	NA	NA	NA	NA
	Powder	0.08%	0.00%	1.26%	0.92%	<0.01	15.726	11.453	**<0.001**	**<0.001**	**<0.001**	**<0.001**	**<0.001**	**<0.001**
	**Cubes**	0.08%	0.00%	1.18%	0.81%	<0.01	14.282	9.801	**<0.001**	**<0.001**	**<0.001**	**<0.001**	**<0.001**	**<0.001**
Lachnospira	Control	0.92%	0.36%	0.09%	0.00%	0.39	0.10	0.00	0.133	**<0.001**	**<0.001**	0.174	**<0.001**	**<0.001**
	Inulin	0.85%	0.36%	0.07%	0.00%	0.425	0.085	<0.01	0.231	0.001	1.000	0.252	0.001	1.000
	Powder	1.02%	0.29%	0.00%	0.00%	0.282	<0.01	<0.01	**<0.001**	**<0.001**	**<0.001**	**<0.001**	**<0.001**	**<0.001**
	**Cubes**	1.09%	0.25%	0.00%	0.30%	0.232	<0.01	0.275	**0.003**	**0.001**	**0.003**	**0.004**	**0.001**	**0.004**
Lachnospiraceae ND3007 group	Control	3.54%	1.21%	0.46%	0.30%	0.34	0.13	0.08	**0.001**	**<0.001**	**<0.001**	**0.002**	**<0.001**	**<0.001**
	Inulin	3.92%	1.16%	0.54%	0.38%	0.296	0.137	0.098	**<0.001**	**<0.001**	**<0.001**	**<0.001**	**<0.001**	**<0.001**
	Powder	3.74%	1.10%	0.29%	0.32%	0.295	0.078	0.085	**<0.001**	**<0.001**	**<0.001**	**<0.001**	**<0.001**	**<0.001**
	**Cubes**	4.00%	1.15%	0.43%	0.28%	0.287	0.107	0.069	**<0.001**	**<0.001**	**<0.001**	**<0.001**	**<0.001**	**<0.001**
Lachnospiraceae UCG-004	Control	0.00%	0.00%	0.38%	0.39%	NA	NA	NA	0.765	**<0.001**	**<0.001**	0.881	**<0.001**	**<0.001**
	Inulin	0.00%	0.00%	0.50%	0.56%	NA	NA	NA	0.618	**<0.001**	**<0.001**	0.660	**<0.001**	**<0.001**
	Powder	0.00%	0.00%	0.26%	0.30%	NA	NA	NA	1.000	**<0.001**	**<0.001**	1.000	**<0.001**	**<0.001**
	**Cubes**	0.08%	0.00%	0.34%	0.35%	<0.01	4.193	4.282	1.000	**0.038**	**0.035**	1.000	**0.040**	**0.040**
Lachnospiraceae unidentified Genus	Control	13.68%	2.53%	0.69%	1.77%	0.19	0.05	0.13	**<0.001**	**<0.001**	**<0.001**	**<0.001**	**<0.001**	**<0.001**
	Inulin	12.73%	2.02%	2.34%	0.72%	0.159	0.184	0.057	**<0.001**	**<0.001**	**<0.001**	**<0.001**	**<0.001**	**<0.001**
	Powder	16.41%	1.95%	2.00%	0.66%	0.119	0.122	0.040	**<0.001**	**<0.001**	**<0.001**	**<0.001**	**<0.001**	**<0.001**
	**Cubes**	14.75%	1.49%	1.30%	0.50%	0.101	0.088	0.034	**<0.001**	**<0.001**	**<0.001**	**<0.001**	**<0.001**	**<0.001**

Taxon	Product	relative abundance (%)				fold-Δ			*p-value*			*q-value*		
		**0h**	**6h**	**24h**	**48h**	**0-6 h**	**0-24 h**	**0-48 h**	**0-6 h**	**0-24 h**	**0-48 h**	**0-6 h**	**0-24 h**	**0-48 h**
Monoglobus	Control	0.51%	0.16%	0.00%	0.00%	0.32	0.00	0.00	**0.016**	**0.001**	**0.001**	**0.024**	**0.001**	**0.001**
	Inulin	0.44%	0.18%	0.00%	0.00%	0.404	<0.01	<0.01	**<0.001**	**<0.001**	**<0.001**	**<0.001**	**<0.001**	**<0.001**
	Powder	0.50%	0.16%	0.00%	0.49%	0.319	<0.01	0.974	**<0.001**	0.999	0.624	**<0.001**	1.000	0.716
	**Cubes**	0.53%	0.09%	0.00%	0.32%	0.167	<0.01	0.608	**0.012**	1.000	0.484	**0.016**	1.000	0.500
Muribaculaceae unidentified Genus	Control	6.21%	2.73%	1.91%	1.73%	0.44	0.31	0.28	**<0.001**	**<0.001**	**<0.001**	**<0.001**	**<0.001**	**<0.001**
	Inulin	7.11%	1.60%	1.23%	1.60%	0.225	0.173	0.225	**<0.001**	**<0.001**	**<0.001**	**<0.001**	**<0.001**	**<0.001**
	Powder	5.82%	2.34%	1.40%	1.20%	0.402	0.241	0.206	**<0.001**	**<0.001**	**<0.001**	**<0.001**	**<0.001**	**<0.001**
	**Cubes**	5.51%	1.64%	1.34%	1.59%	0.296	0.243	0.289	**<0.001**	**<0.001**	**<0.001**	**<0.001**	**<0.001**	**<0.001**
Parabacteroides	Control	0.37%	1.82%	2.78%	3.57%	4.90	7.48	9.59	**0.034**	**0.003**	**<0.001**	**0.048**	**0.004**	**<0.001**
	Inulin	0.44%	1.78%	4.69%	4.78%	4.013	10.568	10.788	**<0.001**	**<0.001**	**<0.001**	**<0.001**	**<0.001**	**<0.001**
	Powder	0.39%	1.82%	4.12%	4.32%	4.689	10.640	11.143	**<0.001**	**<0.001**	**<0.001**	**<0.001**	**<0.001**	**<0.001**
	**Cubes**	0.41%	1.59%	2.92%	4.10%	3.842	7.042	9.882	**<0.001**	**<0.001**	**<0.001**	**<0.001**	**<0.001**	**<0.001**
Paraprevotella	Control	1.05%	0.43%	0.17%	0.04%	0.41	0.16	0.04	0.212	**<0.001**	**<0.001**	0.255	**<0.001**	**<0.001**
	Inulin	0.67%	0.25%	0.12%	0.09%	0.375	0.173	0.138	0.114	**0.005**	**0.001**	0.136	**0.005**	**0.002**
	Powder	1.01%	0.70%	0.24%	0.27%	0.697	0.238	0.265	**<0.001**	**<0.001**	**<0.001**	**<0.001**	**<0.001**	**<0.001**
	**Cubes**	1.21%	0.26%	0.19%	0.20%	0.217	0.161	0.166	**<0.001**	**<0.001**	**<0.001**	**<0.001**	**<0.001**	**<0.001**
Parasutterella	Control	1.06%	2.46%	2.49%	3.43%	2.31	2.34	3.22	**<0.001**	**<0.001**	**<0.001**	**<0.001**	**<0.001**	**<0.001**
	Inulin	1.10%	1.60%	1.28%	1.37%	1.450	1.165	1.243	**<0.001**	**0.003**	**<0.001**	**<0.001**	**0.004**	**<0.001**
	Powder	1.12%	3.06%	2.90%	2.73%	2.727	2.584	2.432	**0.010**	**0.009**	**0.009**	**0.013**	**0.012**	**0.012**
	**Cubes**	1.06%	3.26%	2.54%	3.20%	3.089	2.407	3.025	**<0.001**	**<0.001**	**<0.001**	**<0.001**	**<0.001**	**<0.001**
Phascolarctobacterium	Control	1.51%	5.05%	5.03%	3.34%	3.35	3.33	2.21	**<0.001**	**<0.001**	**0.005**	**<0.001**	**<0.001**	**0.007**
	Inulin	1.52%	4.09%	11.74%	14.50%	2.680	7.701	9.509	**<0.001**	**<0.001**	**<0.001**	**<0.001**	**<0.001**	**<0.001**
	Powder	2.35%	5.66%	12.54%	15.02%	2.410	5.339	6.393	**<0.001**	**<0.001**	**<0.001**	**<0.001**	**<0.001**	**<0.001**
	**Cubes**	1.94%	3.56%	12.29%	11.44%	1.838	6.349	5.913	**0.001**	**<0.001**	**<0.001**	**0.001**	**<0.001**	**<0.001**

Taxon	Product	relative abundance (%)				fold-Δ			*p-value*			*q-value*		
		0h	6h	24h	48h	0-6 h	0-24 h	0-48 h	0-6 h	0-24 h	0-48 h	0-6 h	0-24 h	0-48 h
Roseburia	Control	1.89%	0.20%	0.00%	0.00%	0.11	0.00	0.00	**<0.001**	**<0.001**	**<0.001**	**<0.001**	**<0.001**	**<0.001**
	Inulin	1.71%	0.13%	0.00%	0.00%	0.078	<0.01	<0.01	**<0.001**	**<0.001**	**<0.001**	**<0.001**	**<0.001**	**<0.001**
	Powder	2.17%	0.14%	0.00%	0.19%	0.064	<0.01	0.086	**<0.001**	**<0.001**	**<0.001**	**<0.001**	**<0.001**	**<0.001**
	**Cubes**	2.15%	0.04%	0.00%	0.25%	0.020	<0.01	0.116	**<0.001**	**<0.001**	**<0.001**	**<0.001**	**<0.001**	**<0.001**
Ruminococcus	Control	7.60%	2.40%	1.04%	1.34%	0.32	0.14	0.18	**<0.001**	**<0.001**	**<0.001**	**<0.001**	**<0.001**	**<0.001**
	Inulin	7.38%	2.10%	1.96%	1.61%	0.285	0.265	0.218	**<0.001**	**<0.001**	**<0.001**	**<0.001**	**<0.001**	**<0.001**
	Powder	7.20%	3.90%	3.27%	3.64%	0.541	0.454	0.505	**<0.001**	**<0.001**	**<0.001**	**<0.001**	**<0.001**	**<0.001**
	**Cubes**	10.67%	1.86%	1.74%	1.86%	0.174	0.163	0.174	**0.010**	**0.009**	**0.010**	**0.014**	**0.010**	**0.011**
Streptococcus	Control	0.00%	0.35%	0.20%	0.16%	NA	NA	NA	1.000	1.000	1.000	1.000	1.000	1.000
	Inulin	0.00%	11.83%	9.19%	3.94%	NA	NA	NA	NA	NA	NA	NA	NA	NA
	Powder	0.00%	8.83%	6.17%	4.37%	NA	NA	NA	1.000	1.000	1.000	1.000	1.000	1.000
	**Cubes**	0.00%	14.88%	11.17%	5.38%	NA	NA	NA	**<0.001**	**<0.001**	**<0.001**	**<0.001**	**<0.001**	**<0.001**
Subdoligranulum	Control	9.11%	4.48%	1.29%	1.13%	0.49	0.14	0.12	**<0.001**	**<0.001**	**<0.001**	**<0.001**	**<0.001**	**<0.001**
	Inulin	9.01%	2.62%	0.96%	1.01%	0.291	0.107	0.113	**<0.001**	**<0.001**	**<0.001**	**<0.001**	**<0.001**	**<0.001**
	Powder	10.90%	2.62%	0.86%	0.89%	0.240	0.079	0.082	**0.005**	**0.002**	**0.002**	**0.007**	**0.002**	**0.002**
	**Cubes**	10.44%	2.15%	1.04%	1.06%	0.206	0.099	0.101	**<0.001**	**<0.001**	**<0.001**	**<0.001**	**<0.001**	**<0.001**
Oscillospiraceae UCG-002	Control	0.81%	0.12%	0.13%	0.64%	0.15	0.16	0.79	**0.012**	**0.011**	0.535	**0.018**	**0.014**	0.595
	Inulin	0.73%	0.07%	0.00%	0.00%	0.098	<0.01	<0.01	NA	NA	NA	NA	NA	NA
	Powder	0.85%	0.09%	0.00%	0.28%	0.101	<0.01	0.330	**0.001**	1.000	0.119	**0.002**	1.000	0.148
	**Cubes**	1.01%	0.00%	0.00%	0.00%	<0.01	<0.01	<0.01	NA	NA	NA	NA	NA	NA
Eubacterium eligens group	Control	1.01%	0.61%	0.15%	0.00%	0.61	0.15	0.00	**<0.001**	**<0.001**	**<0.001**	**<0.001**	**<0.001**	**<0.001**
	Inulin	1.07%	0.53%	0.00%	0.00%	0.492	<0.01	<0.01	**<0.001**	**<0.001**	**<0.001**	**<0.001**	**<0.001**	**<0.001**
	Powder	1.23%	0.35%	0.00%	1.11%	0.284	<0.01	0.904	**<0.001**	**<0.001**	0.401	**<0.001**	**<0.001**	0.478
	**Cubes**	1.36%	0.34%	0.00%	1.15%	0.248	<0.01	0.852	**<0.001**	**<0.001**	0.450	**<0.001**	**<0.001**	0.480

Taxon	Product	relative abundance (%)				fold-Δ			*p-value*			*q-value*		
		0h	6h	24h	48h	0-6 h	0-24 h	0-48 h	0-6 h	0-24 h	0-48 h	0-6 h	0-24 h	0-48 h
Eubacterium hallii group	Control	0.24%	0.09%	0.00%	0.14%	0.37	0.00	0.56	0.148	1.000	0.404	0.185	1.000	0.466
	Inulin	0.20%	0.04%	0.00%	0.86%	0.195	<0.01	4.359	0.124	**0.026**	**0.026**	0.141	**0.028**	**0.030**
	Powder	0.27%	0.05%	0.04%	0.48%	0.169	0.152	1.765	0.227	0.201	0.699	0.261	0.249	0.774
	**Cubes**	0.26%	0.00%	0.00%	0.56%	<0.01	<0.01	2.166	**<0.001**	**<0.001**	**<0.001**	**<0.001**	**<0.001**	**<0.001**
Ruminococcus torques group	Control	1.14%	0.79%	10.18%	15.12%	0.69	8.90	13.21	**0.001**	**<0.001**	**<0.001**	**0.002**	**<0.001**	**<0.001**
	Inulin	1.04%	0.90%	6.77%	7.27%	0.858	6.484	6.969	**0.008**	**<0.001**	**<0.001**	**0.011**	**<0.001**	**<0.001**
	Powder	1.31%	1.31%	7.45%	8.80%	1.002	5.695	6.723	0.927	**<0.001**	**<0.001**	1.000	**<0.001**	**<0.001**
	**Cubes**	1.30%	1.02%	8.43%	9.50%	0.782	6.467	7.288	**0.021**	**<0.001**	**<0.001**	**0.027**	**<0.001**	**<0.001**

fold-Δ: fold-change in mean relative abundances over baseline for each condition (control or inulin or dried chicory root powder or cube fermentation); NA: represents p- and q-values that could not be reliably estimated due to violation of the assumption underlying the statistical models for calculating statistical inference [15]

The time-dependent development in overall and within sample gut microbiota composition was reflected in changes of individual genera over time. At the genus level we observed numerous taxa in all conditions to decrease rapidly within 6 h and plateauing at 24 h of fermentation (Supplementary Table 1). The most abundant taxa (mean ± SE relative abundance across all conditions) that decreased were *Faecalibacterium* spp. (16.82 ± 0.71%), an unidentified genus of *Lachnospiraceae* (14.39 ± 0.79%), *Subdoligranulum* spp. (9.87 ± 0.48%) and *Christensenellaceae* R7 group (8.13 ± 0.28%), all decreasing to 1% or less relative abundance over the course of 48 h. For *Faecalibacterium* spp. these decreases were consistent across all conditions and levels did not differ between conditions (Supplementary Table 2). In contrast to the rapid decreases between 0 to 6 h of fermentation, taxa increasing over time underwent the largest fold-changes between 6 to 24 h of fermentation. The highest fold-changes (fold-change ± SE) were observed for *Bacteroides* spp. (24 h: 20.3 ± 4.5 fold), *Lachnoclostridium* spp. (24 h: 13.0 ± 0.7 fold), *Parabacteroides* spp. (48 h: 10.4 ± 0.4 fold) as well as *Akkermansia* spp. (24 h: 11.1 ± 6.0 fold) and bacteria of the *Ruminococcus*

torques group (48h: 8.55 ± 1.56 fold) - for the two latter increasing the most in the control incubations. A number of taxa peaked at 6 h but then decreased again, which included mainly *Streptococcus* spp., with the highest levels at 6 h observed for dried chicory root cubes and inulin, as well as *Parasutterella* spp. with the highest levels in dried chicory root cubes and powder (Supplementary Table 1). We also observed a number of genera to first decrease between 0 to 6 h but then increase again between 6 to 24 and 48 h. The abundant *Blautia* spp. decreased in all conditions at 6 h but increased back to half its baseline levels for inulin and dried chicory root powder and cubes at 48 h. Similarly, bacteria from the *Eubacterium hallii* group decreased in all conditions and were not detected at 6 or 24 h, but then increased again above baseline levels at 48 h in inulin (4.5-fold) and dried chicory root powder (1.8-fold) and cubes (2.2-fold).

Supplementary Table 2. Mean relative abundances of common genera (mean relative abundance of at least 1% and mean prevalence of 50% in the whole dataset) at each timepoint (baseline 0 h, 6 h, 24 h, and 48 h) in the negative control fermentation (Control; only inoculum) or fermentation of inulin (Inulin) vs dried chicory root powder (Powder) or cubes (Cubes) and the respective fold-difference (fold-Δ) between the conditions as well as respective p-values and fdr-corrected q-values.

	**relative abundance (%)**				**fold-Δ compared to Inulin**			***p-value* compared to Inulin**			***q-value* compared to Inulin**		
**Taxon**	**Control**	**Inulin**	**Powder**	**Cubes**	**Control**	**Powder**	**Cubes**	**Control**	**Powder**	**Cubes**	**Control**	**Powder**	**Cubes**
	**Time 0 h**												
Adlercreutzia	0.42%	0.34%	0.35%	0.34%	1.24	1.05	1.01	0.599	0.825	0.943	0.599	0.891	0.979
Akkermansia	1.12%	1.19%	0.93%	1.13%	0.94	0.78	0.95	0.909	0.510	0.873	0.909	0.751	0.943
Bacteroides	0.09%	0.38%	0.08%	0.13%	0.24	0.22	0.34	0.599	0.256	0.337	0.599	0.540	0.660
Blautia	8.24%	8.08%	8.50%	8.18%	1.02	1.05	1.01	0.909	0.471	0.857	0.909	0.749	0.943
Christensenellaceae R7 group	9.20%	8.26%	8.61%	8.24%	1.11	1.04	1.00	0.625	0.743	0.986	0.625	0.848	0.986
Dorea	0.80%	0.73%	0.75%	0.82%	1.09	1.03	1.12	0.599	0.741	0.178	0.599	0.848	0.497
Erysipelotrichaceae UCG 003	3.85%	3.46%	2.88%	3.19%	1.11	0.83	0.92	0.599	**0.045**	0.297	0.599	0.221	0.660
Faecalibacterium	17.31%	17.79%	18.66%	17.21%	0.97	1.05	0.97	0.909	0.560	0.795	0.909	0.756	0.943
Fusicatenibacter	2.47%	2.21%	2.51%	2.39%	1.12	1.14	1.08	0.599	0.139	0.342	0.599	0.382	0.660
Haemophilus	0.00%	0.09%	0.00%	0.09%	<0.01	<0.01	1.03	NA	NA	NA	NA	NA	NA
Lachnoclostridium	0.11%	0.00%	0.08%	0.08%	NA	NA	NA	<0.001	0.092	0.088	**<0.001**	0.310	0.395
Lachnospira	1.02%	0.96%	1.02%	1.09%	1.05	1.06	1.13	0.757	0.528	0.203	0.757	0.751	0.497
Lachnospiraceae ND3007 group	3.91%	4.54%	3.74%	4.00%	0.86	0.82	0.88	0.659	0.347	0.540	0.659	0.625	0.810
Lachnospiraceae unidentified Genus	15.09%	14.53%	16.41%	14.75%	1.04	1.13	1.01	0.659	0.026	0.762	0.659	0.221	0.943
Monoglobus	0.56%	0.50%	0.50%	0.53%	1.13	1.00	1.06	0.599	0.997	0.667	0.599	0.997	0.943
Muribaculaceae unidentified Genus	6.85%	8.20%	5.82%	5.51%	0.84	0.71	0.67	0.599	0.047	**0.024**	0.599	0.221	0.217
Parabacteroides	0.41%	0.51%	0.39%	0.41%	0.80	0.76	0.81	0.599	0.051	0.190	0.599	0.221	0.497
Paraprevotella	1.16%	0.78%	1.01%	1.21%	1.48	1.29	1.54	0.524	0.230	0.083	0.524	0.540	0.395
Parasutterella	1.17%	1.26%	1.12%	1.06%	0.93	0.89	0.84	0.599	0.057	**0.010**	0.599	0.221	0.217

Taxon	relative abundance (%)				fold-Δ compared to Inulin			*p-value* compared to Inulin			*q-value* compared to Inulin		
	Control	Inulin	Powder	Cubes	Control	Powder	Cubes	Control	Powder	Cubes	Control	Powder	Cubes
Phascolarctobacterium	1.67%	1.73%	2.35%	1.94%	0.96	1.36	1.12	0.911	0.289	0.717	0.911	0.557	0.943
Roseburia	2.09%	1.94%	2.17%	2.15%	1.07	1.11	1.11	0.599	0.056	0.071	0.599	0.221	0.395
Ruminococcus	8.37%	8.48%	7.20%	10.67%	0.99	0.85	1.26	0.909	**0.045**	0.373	0.909	0.221	0.665
Subdoligranulum	10.03%	10.23%	10.90%	10.44%	0.98	1.07	1.02	0.909	0.454	0.805	0.909	0.749	0.943
UCG 002	0.89%	0.83%	0.85%	1.01%	1.08	1.03	1.22	0.599	0.716	**0.022**	0.599	0.848	0.217
Eubacterium eligens group	1.11%	1.23%	1.23%	1.36%	0.90	1.00	1.10	0.707	0.980	0.503	0.707	0.997	0.798
Eubacterium hallii group	0.27%	0.22%	0.27%	0.26%	1.20	1.22	1.17	0.599	0.260	0.394	0.599	0.540	0.665
Eubacterium ventriosum group	0.48%	0.33%	0.35%	0.43%	1.45	1.05	1.28	0.365	0.754	0.125	0.365	0.848	0.482
Ruminococcus torques group	1.26%	1.19%	1.31%	1.30%	1.06	1.10	1.09	0.599	0.142	0.156	0.599	0.382	0.497
							**Time 6 h**						
Adlercreutzia	0.84%	0.63%	0.51%	0.40%	1.34	0.81	0.64	0.089	0.312	0.161	0.258	0.476	0.312
Akkermansia	3.82%	2.26%	1.83%	1.51%	1.70	0.81	0.67	0.086	0.402	0.217	0.258	0.545	0.370
Bacteroides	1.38%	2.23%	1.20%	1.61%	0.62	0.54	0.72	0.386	0.245	0.546	0.746	0.394	0.688
Blautia	2.83%	2.33%	2.57%	2.22%	1.21	1.11	0.96	**0.050**	**<0.001**	0.625	0.200	**0.001**	0.713
Christensenellaceae R7 group	1.73%	1.24%	1.59%	0.82%	1.40	1.29	0.66	0.301	0.453	0.380	0.624	0.571	0.500
Collinsella	1.40%	2.65%	0.00%	0.33%	0.53	<0.01	0.13	0.991	**0.005**	0.178	0.991	**0.017**	0.323
Dorea	0.30%	0.27%	0.46%	0.35%	1.14	1.72	1.30	0.696	**0.002**	0.064	0.916	**0.010**	0.211
Eggerthella	0.15%	0.03%	0.12%	0.00%	4.51	3.63	<0.01	0.055	0.068	0.330	0.200	0.165	0.500
Enterobacteriaceae unidentified Genus	1.19%	0.67%	0.28%	0.97%	1.78	0.41	1.45	0.420	0.109	0.664	0.760	0.244	0.713
Erysipelotrichaceae UCG-003	1.33%	0.61%	0.50%	0.44%	2.18	0.81	0.73	**<0.001**	0.168	**0.038**	**0.004**	0.304	0.211
Escherichia Shigella	50.81%	48.96%	51.40%	53.84%	1.04	1.05	1.10	0.790	0.564	0.348	0.916	0.654	0.500
Faecalibacterium	5.12%	4.08%	4.42%	3.30%	1.26	1.08	0.81	0.156	0.621	0.274	0.376	0.687	0.442
Fusicatenibacter	0.50%	0.49%	0.38%	0.35%	1.03	0.78	0.71	0.859	0.164	0.076	0.922	0.304	0.211
Haemophilus	0.35%	0.33%	0.37%	0.28%	1.05	1.11	0.86	0.777	0.413	0.104	0.916	0.545	0.243

Taxon	relative abundance (%)				fold-Δ compared to Inulin			*p-value* compared to Inulin			*q-value* compared to Inulin		
	Control	Inulin	Powder	Cubes	Control	Powder	Cubes	Control	Powder	Cubes	Control	Powder	Cubes
Lachnospira	0.39%	0.39%	0.29%	0.25%	1.01	0.75	0.66	0.971	0.182	0.080	0.991	0.310	0.211
Lachnospiraceae ND3007 group	1.35%	1.24%	1.10%	1.15%	1.09	0.89	0.92	0.758	0.689	0.782	0.916	0.713	0.810
Lachnospiraceae unidentified Genus	2.77%	2.15%	1.95%	1.49%	1.29	0.91	0.69	0.033	**0.044**	**0.001**	0.189	0.116	**0.005**
Monoglobus	0.18%	0.19%	0.16%	0.09%	0.94	0.84	0.46	0.848	**0.008**	**0.050**	0.922	**0.027**	0.211
Muribaculaceae unidentified Genus	3.01%	1.70%	2.34%	1.64%	1.77	1.38	0.96	0.045	**<0.001**	0.642	0.200	**0.002**	0.713
Parabacteroides	2.01%	1.89%	1.82%	1.59%	1.06	0.96	0.84	0.715	0.521	0.031	0.916	0.630	0.211
Paraprevotella	0.47%	0.27%	0.70%	0.26%	1.76	2.61	0.97	0.177	**<0.001**	0.861	0.395	**0.002**	0.861
Parasutterella	2.72%	1.70%	3.06%	3.26%	1.60	1.80	1.92	**0.001**	**<0.001**	**<0.001**	**0.004**	**0.001**	**0.001**
Phascolarctobacterium	5.44%	4.33%	5.66%	3.56%	1.26	1.31	0.82	0.625	0.391	0.372	0.916	0.545	0.500
Roseburia	0.22%	0.14%	0.14%	0.04%	1.56	0.98	0.30	0.130	0.898	0.059	0.344	0.898	0.211
Ruminococcus	2.66%	2.24%	3.90%	1.86%	1.19	1.74	0.83	0.696	**0.001**	0.066	0.916	**0.002**	0.211
Streptococcus	0.37%	12.56%	8.83%	14.88%	0.03	0.70	1.18	**<0.001**	**<0.001**	**<0.001**	**<0.001**	**0.002**	**0.004**
Subdoligranulum	4.90%	2.79%	2.62%	2.15%	1.76	0.94	0.77	**<0.001**	0.640	0.109	0.004	0.687	0.243
Oscillospiraceae UCG-002	0.13%	0.08%	0.09%	0.00%	1.72	1.11	<0.01	NA	NA	NA	NA	NA	NA
Eubacterium eligens group	0.67%	0.56%	0.35%	0.34%	1.18	0.62	0.60	0.567	0.162	0.152	0.914	0.304	0.312
Eubacterium hallii group	0.10%	0.04%	0.05%	0.00%	2.38	1.15	<0.01	NA	NA	NA	NA	NA	NA
Ruminococcus torques group	0.86%	0.96%	1.31%	1.02%	0.90	1.37	1.07	0.528	0.035	0.664	0.900	0.103	0.713
						**Time 24 h**							
Adlercreutzia	0.60%	0.50%	0.38%	0.52%	1.21	0.77	1.05	0.171	0.132	0.706	0.231	0.274	0.901
Akkermansia	30.94%	3.84%	5.61%	6.69%	8.06	1.46	1.74	**<0.00**1	0.442	0.143	**0.001**	0.550	0.317
Bacteroides	1.62%	2.74%	2.47%	3.19%	0.59	0.90	1.16	0.058	0.521	0.788	0.093	0.612	0.901
Blautia	0.70%	0.86%	4.18%	3.63%	0.81	4.88	4.24	0.232	**<0.001**	**<0.001**	0.299	**<0.001**	**0.001**
Christensenellaceae R7 group	0.70%	0.42%	0.37%	0.39%	1.67	0.89	0.94	**0.034**	0.677	0.834	0.068	0.703	0.901
Collinsella	2.88%	1.44%	4.05%	0.88%	2.00	2.82	0.61	0.939	0.628	0.319	0.939	0.703	0.575

	**relative abundance (%)**				**fold-Δ compared to Inulin**			***p-value* compared to Inulin**			***q-value* compared to Inulin**		
**Taxon**	**Control**	**Inulin**	**Powder**	**Cubes**	**Control**	**Powder**	**Cubes**	**Control**	**Powder**	**Cubes**	**Control**	**Powder**	**Cubes**
Dorea	3.15%	11.48%	10.72%	6.65%	0.27	0.93	0.58	**<0.001**	0.202	**0.002**	**<0.001**	0.389	**0.007**
Eggerthella	0.14%	0.13%	0.11%	0.13%	1.07	0.86	1.02	0.682	0.276	0.893	0.736	0.447	0.927
Enterobacteriaceae unidentified Genus	0.38%	0.30%	0.22%	0.54%	1.28	0.75	1.80	0.569	0.448	0.389	0.673	0.550	0.617
Erysipelotrichaceae UCG-003	0.35%	0.26%	0.23%	0.25%	1.35	0.86	0.96	0.148	0.245	0.745	0.210	0.441	0.901
Escherichia-Shigella	27.74%	32.46%	27.27%	30.62%	0.85	0.84	0.94	0.105	0.080	0.497	0.158	0.179	0.745
Faecalibacterium	0.63%	0.68%	0.56%	0.77%	0.93	0.82	1.12	0.714	0.384	0.550	0.742	0.544	0.781
Haemophilus	0.25%	0.45%	0.74%	0.60%	0.54	1.64	1.32	**<0.001**	**0.006**	**0.017**	**<0.001**	**0.021**	0.052
Lachnoclostridium	1.00%	0.96%	1.26%	1.18%	1.05	1.31	1.24	0.616	0.281	0.298	0.693	0.447	0.575
Lachnospira	0.10%	0.08%	0.00%	0.00%	1.28	<0.01	<0.01	NA	NA	NA	NA	NA	NA
Lachnospiraceae ND3007 group	0.49%	0.56%	0.29%	0.43%	0.87	0.52	0.76	0.573	0.066	0.371	0.673	0.179	0.617
Lachnospiraceae UCG-004	0.41%	0.52%	0.26%	0.34%	0.78	0.49	0.65	**0.003**	**<0.001**	**<0.001**	**0.008**	**<0.001**	**0.002**
Lachnospiraceae unidentified Genus	0.73%	2.45%	2.00%	1.30%	0.30	0.82	0.53	**<0.001**	**0.005**	**0.001**	**<0.001**	0.019	0.005
Muribaculaceae unidentified Genus	2.04%	1.29%	1.40%	1.34%	1.58	1.09	1.04	**<0.001**	0.403	0.720	**0.002**	0.544	0.901
Parabacteroides	2.96%	4.91%	4.12%	2.92%	0.60	0.84	0.59	**0.001**	0.076	**0.001**	**0.003**	0.179	**0.005**
Paraprevotella	0.18%	0.12%	0.24%	0.19%	1.44	1.96	1.60	0.055	**0.001**	**0.016**	0.092	**0.006**	0.052
Parasutterella	2.65%	1.34%	2.90%	2.54%	1.97	2.16	1.89	**<0.001**	**0.003**	**<0.001**	**<0.001**	**0.014**	**0.003**
Phascolarctobacterium	5.35%	12.30%	12.54%	12.29%	0.43	1.02	1.00	**0.014**	0.916	0.995	0.032	0.916	0.995
Ruminococcus	1.11%	2.05%	3.27%	1.74%	0.54	1.60	0.85	**0.001**	**0.001**	0.108	**0.002**	**0.006**	0.266
Streptococcus	0.21%	9.62%	6.17%	11.17%	0.02	0.64	1.16	**<0.001**	**<0.001**	**<0.001**	**<0.001**	**0.004**	0.002
Subdoligranulum	1.37%	1.01%	0.86%	1.04%	1.36	0.86	1.03	**0.035**	0.338	0.830	0.068	0.508	0.901
Eubacterium ventriosum group	0.21%	0.10%	0.25%	0.21%	2.16	2.55	2.14	**0.039**	**0.011**	**0.042**	0.070	**0.033**	0.114
Ruminococcus torques group	10.84%	7.08%	7.45%	8.43%	1.53	1.05	1.19	**0.002**	0.677	0.152	**0.006**	0.703	0.317

Taxon	relative abundance (%) Control	Inulin	Powder	Cubes	fold-Δ compared to Inulin Control	Powder	Cubes	*p-value* compared to Inulin Control	Powder	Cubes	*q-value* compared to Inulin Control	Powder	Cubes
	**Time 48 h**												
Adlercreutzia	0.29%	0.31%	0.44%	0.47%	0.93	1.39	1.49	0.894	0.470	0.383	0.894	0.616	0.547
Akkermansia	21.42%	4.89%	7.16%	7.72%	4.38	1.46	1.58	**<0.001**	0.031	0.314	**<0.001**	0.071	0.470
Bacteroides	0.76%	2.51%	2.62%	1.82%	0.30	1.04	0.72	**0.005**	0.815	0.165	**0.011**	0.844	0.309
Blautia	0.77%	4.16%	4.42%	4.35%	0.18	1.06	1.05	**<0.001**	0.606	0.774	**0.001**	0.699	0.862
Christensenellaceae R7 group	1.02%	0.46%	0.41%	0.49%	2.20	0.87	1.06	**0.001**	0.493	0.596	**0.002**	0.616	0.716
Collinsella	0.26%	0.07%	3.06%	0.12%	3.80	44.57	1.72	NA	NA	NA	NA	NA	NA
Dorea	3.44%	3.32%	1.54%	1.43%	1.04	0.47	0.43	0.811	**0.006**	**0.004**	0.839	**0.016**	**0.021**
Eggerthella	0.19%	0.13%	0.12%	0.16%	1.43	0.94	1.24	**<0.001**	0.415	**0.032**	**<0.001**	0.593	0.107
Enterobacteriaceae unidentified Genus	0.19%	0.53%	0.26%	0.35%	0.36	0.49	0.65	**0.037**	0.080	0.211	0.073	0.161	0.373
Erysipelotrichaceae UCG-003	0.22%	0.18%	0.19%	0.17%	1.23	1.08	0.94	0.436	0.720	0.799	0.524	0.800	0.862
Escherichia Shigella	35.15%	39.36%	32.84%	39.08%	0.89	0.83	0.99	0.250	0.091	0.935	0.326	0.170	0.935
Faecalibacterium	0.41%	0.59%	0.47%	0.61%	0.69	0.79	1.03	0.153	0.324	0.891	0.230	0.486	0.921
Haemophilus	0.09%	0.33%	0.48%	0.39%	0.26	1.44	1.18	**0.002**	**0.004**	0.107	**0.004**	**0.013**	0.214
Lachnoclostridium	0.97%	1.24%	0.92%	0.81%	0.78	0.74	0.65	0.250	0.181	0.091	0.326	0.320	0.210
Lachnospiraceae ND3007 group	0.32%	0.42%	0.32%	0.28%	0.76	0.76	0.66	0.569	0.574	0.427	0.628	0.688	0.577
Lachnospiraceae UCG 004	0.41%	0.61%	0.30%	0.35%	0.67	0.50	0.58	**0.001**	**<0.001**	**<0.001**	**0.002**	**<0.001**	**0.001**
Lachnospiraceae unidentified Genus	1.85%	0.78%	0.66%	0.50%	2.38	0.85	0.64	0.170	0.302	**0.011**	0.242	0.478	**0.042**
Monoglobus	0.00%	0.00%	0.49%	0.32%	NA	NA	NA	0.113	**<0.001**	**<0.001**	0.178	**<0.001**	**<0.001**
Muribaculaceae unidentified Genus	1.82%	1.73%	1.20%	1.59%	1.05	0.69	0.92	0.458	**0.00**2	0.274	0.529	**0.006**	0.432
Parabacteroides	3.74%	5.18%	4.32%	4.10%	0.72	0.83	0.79	**0.010**	0.081	**0.036**	**0.022**	0.161	0.108
Paraprevotella	0.04%	0.10%	0.27%	0.20%	0.40	2.68	2.01	0.369	**0.016**	0.105	0.461	**0.040**	0.214
Parasutterella	3.60%	1.48%	2.73%	3.20%	2.43	1.84	2.15	**<0.001**	**<0.001**	**<0.001**	**0.001**	**<0.001**	**<0.001**
Phascolarctobacterium	3.50%	15.72%	15.02%	11.44%	0.22	0.96	0.73	**<0.001**	0.776	0.061	**0.001**	0.832	0.154

Taxon	relative abundance (%)				fold-Δ compared to Inulin			*p-value* compared to Inulin			*q-value* compared to Inulin		
	**Control**	**Inulin**	**Powder**	**Cubes**	**Control**	**Powder**	**Cubes**	**Control**	**Powder**	**Cubes**	**Control**	**Powder**	**Cubes**
Roseburia	0.00%	0.00%	0.19%	0.25%	NA	NA	NA	0.105	**<0.001**	**<0.001**	0.175	**<0.001**	**<0.001**
Ruminococcus	1.41%	1.74%	3.64%	1.86%	0.81	2.10	1.07	0.101	**<0.001**	0.447	0.175	**<0.001**	0.577
Streptococcus	0.17%	4.27%	4.37%	5.38%	0.04	1.02	1.26	**<0.001**	0.846	**0.049**	<0.001	0.846	0.134
Subdoligranulum	1.19%	1.10%	0.89%	1.06%	1.08	0.81	0.96	0.586	0.242	0.805	0.628	0.403	0.862
Oscillospiraceae UCG-002	0.67%	0.00%	0.28%	0.00%	NA	NA	NA	**<0.001**	**<0.001**	0.461	**<0.001**	**<0.001**	0.577
Eubacterium eligens group	0.00%	0.00%	1.11%	1.15%	NA	NA	NA	0.104	**<0.001**	**<0.001**	0.175	**<0.001**	**<0.001**
Eubacterium hallii group	0.14%	0.93%	0.48%	0.56%	0.16	0.52	0.61	**<0.001**	**0.002**	**0.008**	**<0.001**	**0.007**	**0.032**
Ruminococcus torques group	15.85%	7.87%	8.80%	9.50%	2.02	1.12	1.21	**<0.001**	0.487	0.237	**0.001**	0.616	0.395
							**48 h mucus**						
Akkermansia	10.04%	0.50%	1.11%	1.12%	20.02	**2.21**	**2.23**	**<0.001**	0.124	**0.050**	**0.002**	0.234	0.106
Bifidobacterium	0.06%	2.32%	2.43%	2.32%	0.02	1.05	1.00	0.009	0.916	1.000	**0.022**	0.974	1.000
Blautia	0.46%	0.87%	1.30%	1.62%	0.53	1.49	1.86	0.113	0.101	0.011	0.160	0.234	**0.048**
Clostridium sensu stricto 1	5.57%	6.45%	0.50%	1.07%	0.86	0.08	0.17	0.368	**<0.001**	**<0.001**	0.447	**<0.001**	**0.001**
Dorea	0.64%	0.39%	0.67%	0.86%	1.65	1.73	2.22	0.009	0.116	0.038	**0.022**	0.234	0.091
Eisenbergiella	0.28%	0.29%	0.18%	0.24%	0.97	0.61	0.81	0.857	0.040	0.273	0.919	0.113	0.387
Escherichia.Shigella	4.63%	2.14%	2.68%	2.92%	2.17	1.25	1.37	**0.008**	0.470	0.305	0.022	0.665	0.399
Lachnoclostridium	0.16%	0.41%	0.00%	0.16%	0.38	0.00	0.40	**0.024**	**<0.001**	**0.030**	0.049	**<0.001**	0.086
Lachnospiraceae unidentified Genus	40.04%	2.97%	15.80%	9.06%	13.46	5.31	3.05	**<0.001**	**<0.001**	**0.002**	**<0.001**	**0.001**	**0.013**
Muribaculaceae unidentified Genus	0.28%	0.10%	0.06%	0.19%	2.91	0.59	1.96	0.026	0.575	0.208	**0.049**	0.752	0.322
Parabacteroides	0.12%	0.29%	0.17%	0.20%	0.40	0.57	0.71	0.153	0.294	0.466	0.200	0.454	0.566
Parasutterella	0.04%	0.06%	0.16%	0.23%	0.74	2.78	4.21	0.865	0.267	0.064	0.919	0.454	0.122
Phascolarctobacterium	3.86%	3.82%	3.53%	1.93%	1.01	0.93	0.50	0.973	0.827	0.142	0.973	0.937	0.242
Roseburia	12.58%	1.20%	24.77%	14.44%	10.47	20.61	12.02	**<0.001**	**0.011**	**<0.001**	**<0.001**	**0.049**	**0.001**

Taxon	relative abundance (%) Control	Inulin	Powder	Cubes	fold-Δ compared to Inulin Control	Powder	Cubes	*p-value* compared to Inulin Control	Powder	Cubes	*q-value* compared to Inulin Control	Powder	Cubes
Streptococcus	0.28%	4.05%	3.48%	4.76%	0.07	0.86	1.17	0.029	0.751	0.721	0.049	0.912	0.817
Eubacterium hallii group	0.00%	0.19%	0.19%	0.16%	0.00	0.98	0.84	0.106	0.978	0.781	0.160	0.978	0.830
Ruminococcus torques group	17.60%	67.81%	42.99%	53.12%	0.26	0.63	0.78	**<0.001**	0.015	0.023	**<0.001**	**0.050**	0.078
Akkermansia	10.04%	0.50%	1.11%	1.12%	20.02	2.21	2.23	**<0.001**	0.124	0.050	**0.002**	0.234	0.106

fold-Δ: fold-difference in mean relative abundances per timepoint of control or dried chicory root powder or cube fermentation compared to inulin fermentation; NA: represents p- and q-values that could not be reliably estimated due to violation of the assumption underlying the statistical models for calculating statistical inference [15]

Comparing differences in relative abundance of genera at each timepoint between inulin and dried chicory root cubes or powder or control (Supplementary Table 2), we observed that the number of taxa statistically significantly differing from inulin increased over time. At 6 h, 12 genera were found to differ in dried chicory root cubes or powder or control from inulin, while at 24 h these were 15 genera and at 48 h 22 genera. The majority of taxa identified to differ statistically significantly from inulin at each time point were contributed by dried chicory root powder, which had the following amounts of statistically significantly different taxa: at 6h nine out of 12 genera, at 24 h nine out of 15 genera and at 48h 12 out 22 genera. For dried chicory root cubes these were at 6 h three of the 12 genera, at 24 h seven out of the 15 genera and at 48 h eight out of the 22 taxa. The taxa with statistically significantly largest positive fold-differences (>1.2-fold)between dried chicory root powder and inulin were *Paraprevotella* spp., *Parasutterella* spp, and *Ruminococcus* spp. at 6 h, 24 and 48 h, *Blautia* spp. at 6 and *Haemophilus* spp. at 24 h and *Eubacterium ventrosium* group and *Colinsella* spp. at 24h, as well as *Dorea* spp. at 6 h and 48 h. Besides *Parasutterella* spp and *Streptococcus* spp for dried chicory root cubes at 6 h and 24 h, *Blautia* spp. and *Dorea* spp. underwent the largest fold-changes statistically significantly different from inulin at 24 h. Both dried chicory root powder and cubes had statistically significantly higher levels of *Monoglobus*

spp., bacteria from the *Eubacterium eligens* group and *Roseburia* spp at 48 h, which were virtually not present in inulin or control. In the respective mucus samples collected at 48 h of fermentation *Bifidobacterium* spp became apparent at low relative levels for all three fibers (Supplementary Figure 7) in contrast to no detected presence in the pellets at any timepoint of the fermentation. Besides higher levels in *Roseburia* spp and *Bifidobacterium* spp, dried chicory root powder and cubes fermentation also resulted in significantly lower levels of the potential pathogen containing genus *Clostridium sensu stricto 1* compared to the control and inulin. The mucin-covered beads were also dominated by bacteria from the known mucin-degrading *Ruminococcus torques* group [16] and we suspect that due to absence of other fiber substrate to feed on *Akkermansia* spp. and bacteria from the *Ruminococcus torques* group were released from the mucin-covered beads explaining their highest level in the control incubation pellets.

Supplementary Table 3. Number of genera found to significantly increase after 48 h of *in vitro* fermentation in the control (Control) or inulin (Inulin), dried chicory root powder (Powder) or cubes (Cubes).

Taxon	**Control**				**Inulin**				**Powder**				**Cubes**			
	**q-value**	**(%)**	**foldΔ**	**Δ%**	**q-value**	**(%)**	**foldΔ**	**Δ%**	**q-value**	**(%)**	**foldΔ**	**Δ%**	**q-value**	**(%)**	**foldΔ**	**Δ%**
1. Akkermansia	<0.001	20.40%	20.20	19.39%	<0.001	4.51%	4.31	3.47%	<0.001	6.73%	8.01	5.89%	<0.001	7.17%	7.02	6.15%
2. Bacteroides	0.001	0.73%	8.99	0.65%	0.002	2.32%	7.41	2.00%	<0.001	2.46%	31.95	2.38%	<0.001	1.69%	14.73	1.58%
3. Coprococcus	-	-	-	-	-	-	-	-	-	-	-	-	<0.001	0.57%	1.24	0.11%
4. Dorea	<0.001	3.28%	4.52	2.55%	<0.001	3.07%	4.75	2.43%	<0.001	1.45%	2.15	0.78%	0.007	1.34%	1.81	0.60%
5. Eggerthella	<0.001	0.18%	NA	0.18%	-	-	-	-	<0.001	0.12%	NA	0.12%	-	-	-	-
***6. Enterobacteriaceae* unidentified Genus**	-	-	-	-	<0.001	0.49%	NA	0.49%	-	-	-	-	<0.001	0.32%	NA	0.32%
7. Eubacterium hallii	-	-	-	-	0.030	0.86%	4.36	0.66%	-	-	-	-	<0.001	0.53%	2.24	0.29%
8. Haemophilus	<0.001	0.08%	NA	0.08%	0.014	0.31%	4.01	0.23%	-	-	-	-	0.009	0.37%	4.54	0.29%
9. Lachnoclostridium	<0.001	0.92%	8.89	0.82%	-	-	-	-	<0.001	0.86%	11.79	0.79%	<0.001	0.76%	9.96	0.68%
***10. Lachnospiraceae* UCG-004**	<0.001	0.39%	NA	0.39%	<0.001	0.56%	NA	0.56%	<0.001	0.28%	NA	0.28%	0.012	0.33%	4.36	0.25%
11. Parabacteroides	<0.001	3.57%	9.59	3.19%	<0.001	4.78%	10.79	4.34%	<0.001	4.06%	11.67	3.71%	<0.001	3.82%	10.20	3.45%
12. Parasutterella	<0.001	3.43%	3.22	2.37%	<0.001	1.37%	1.24	0.27%	0.012	2.57%	2.55	1.56%	<0.001	2.99%	3.13	2.04%
13. Peptostreptococcus	-	-	-	-	<0.001	0.38%	NA	0.38%	-	-	-	-	<0.001	0.61%	NA	0.61%
14. Phascolarcto-bacterium	0.006	3.34%	2.21	1.83%	<0.001	14.50%	9.51	12.97%	<0.001	14.12%	6.71	12.01%	<0.001	10.69%	6.11	8.94%
15. Ruminococcus torques	<0.001	15.12%	13.21	13.97%	<0.001	7.27%	6.97	6.23%	<0.001	8.28%	7.06	7.11%	<0.001	8.91%	7.57	7.73%
16. Streptococcus	-	-	-	-	-	-	-	-	-	-	-	-	0.041	5.03%	NA	5.03%
17. Veillonella	-	-	-	-	-	-	-	-	<0.001	0.56%	NA	0.56%	-	-	-	-

fold-Δ: fold-difference in mean relative abundances at 48 h over baseline per condition; Δ%: absolute change in mean relative abundance at 48 h over baseline

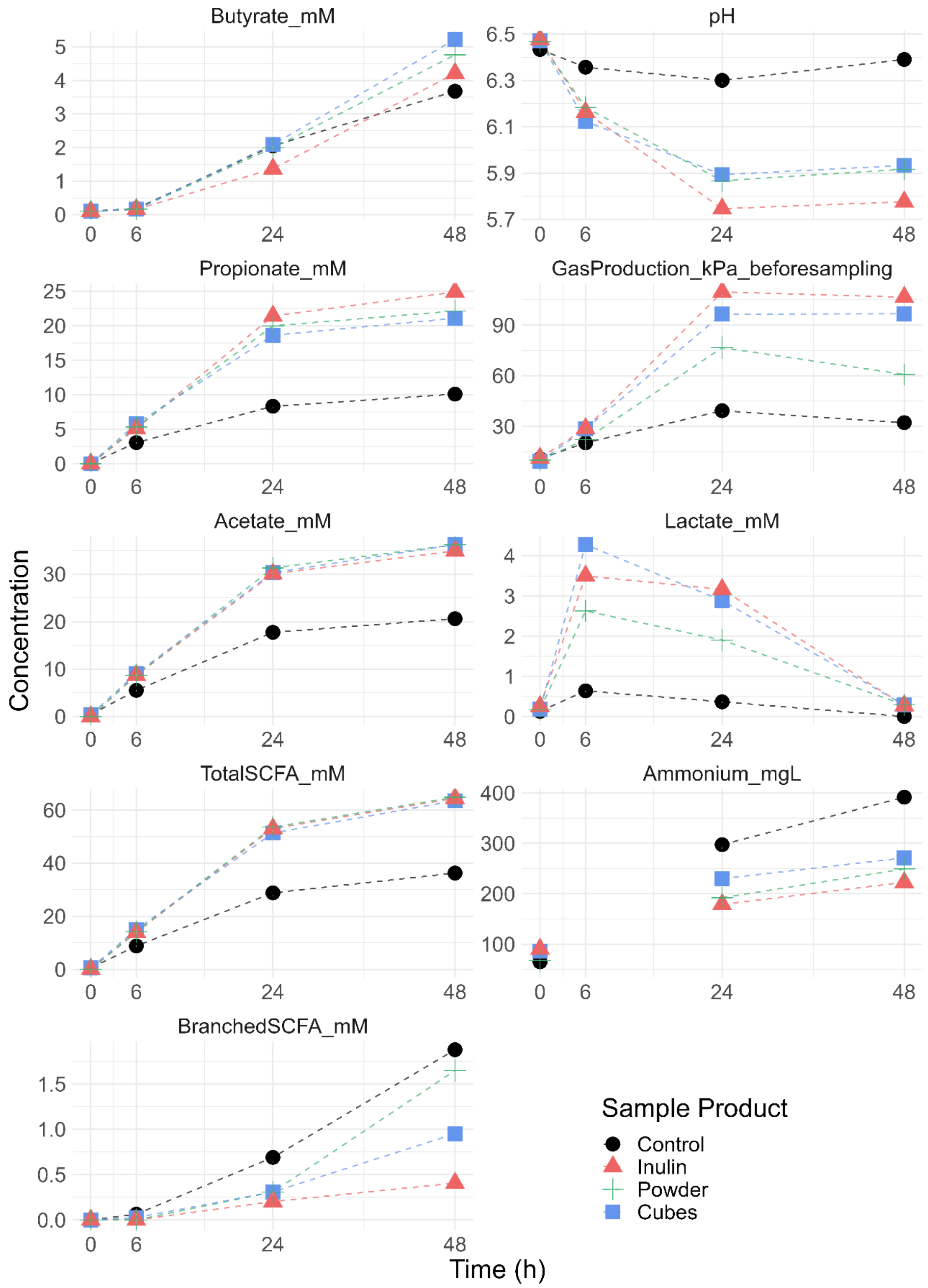

Supplementary Figure 8. Concentrations of pH, gas production levels and fermentation metabolites over time (T = 0h, 6 h, 24 h and 48 h) for control (Control; only inoculum) *in vitro* fermentation and i fermentation of inulin (Inulin) and dried chicory root powder (Powder) and cubes (Cubes).

Supplementary Table 4. Differences in pH, gas production and levels of fermentation metabolites at baseline (T = 0h), 6 h, 24 h and 48 h between control *in vitro* fermentation (Control; only inoculum) and *in vitro* fermentation of inulin (Inulin) and dried chicory root powder (Powder) and cubes (Cubes) and their changes over baseline. Statistical testing was done using by robust ANOVA followed by pairwise comparison using the corresponding Yuen's trimmed means test and fdr-correction (p_{adj}). Data are presented as mean ± SE.

	Time (h)	Control	Inulin	Powder	Cubes	robust ANOVA	pairwise comparisons *(adjusted p-value)* Control vs Inulin	Control vs Powder	Control vs Cubes	Inulin vs Powder	Inulin vs Cubes	Powder vs Cubes
**pH**	0	6.43 ± 0	6.48 ± 0	6.47 ± 0.01	6.47 ± 0.02	F(3, 4.13) = 21.87<br>p = 0.006	p_{adj} = 0.005	p_{adj} = 0.148	p_{adj} = 0.329	p_{adj} = 0.568	p_{adj} = 0.875	p_{adj} = 0.875
	6	6.36 ± 0	6.16 ± 0	6.18 ± 0.01	6.12 ± 0.01	F(3, 4.1) = 453.57<br>p = 0.001	p_{adj} < 0.001	p_{adj} = 0.006	p_{adj} = 0.006	p_{adj} = 0.233	p_{adj} = 0.125	p_{adj} = 0.053
	Δ0-6	-0.08 ± 0	-0.31 ± 0	-0.28 ± 0.01	-0.35 ± 0.03	F(3, 4.06) = 647.97<br>p = 0.001	p_{adj} < 0.001	p_{adj} = 0.01	p_{adj} = 0.02	p_{adj} = 0.203	p_{adj} = 0.362	p_{adj} = 0.203
	24	6.3 ± 0.01	5.75 ± 0.01	5.87 ± 0	5.89 ± 0	F(3, 4.3) = 1355.53<br>p = 0.001	p_{adj} < 0.001	p_{adj} < 0.001	p_{adj} < 0.001	p_{adj} = 0.001	p_{adj} < 0.001	p_{adj} = 0.005
	Δ0-24	-0.13 ± 0	-0.73 ± 0.01	-0.6 ± 0.01	-0.58 ± 0.02	F(3, 4.13) = 2658.83<br>p = 0.001	p_{adj} < 0.001	p_{adj} < 0.001	p_{adj} = 0.002	p_{adj} < 0.001	p_{adj} = 0.014	p_{adj} = 0.37
	Δ6-24	-0.06 ± 0	-0.42 ± 0.01	-0.32 ± 0.01	-0.23 ± 0.01	F(3, 3.82) = 444.84<br>p = 0.001	p_{adj} = 0.001	p_{adj} = 0.003	p_{adj} = 0.004	p_{adj} = 0.006	p_{adj} = 0.001	p_{adj} = 0.008
	48	6.39 ± 0.01	5.78 ± 0.01	5.92 ± 0.01	5.93 ± 0.01	F(3, 4.41) = 1454.29<br>p = 0.001	p_{adj} < 0.001	p_{adj} < 0.001	p_{adj} < 0.001	p_{adj} < 0.001	p_{adj} < 0.001	p_{adj} = 0.211
	Δ0-48	-0.04 ± 0	-0.70 ± 0.01	-0.55 ± 0	-0.54 ± 0.02	n.a.	n.a.	n.a.	n.a.	n.a.	n.a.	n.a.
	Δ24-48	0.09 ± 0	0.03 ± 0.01	0.05 ± 0.01	0.04 ± 0.01	n.a.	n.a.	n.a.	n.a.	n.a.	n.a.	n.a.

							**pairwise comparisons *(adjusted p-value)***					
	**Time (h)**	**Control**	**Inulin**	**Powder**	**Cubes**	**robust ANOVA**	**Control vs Inulin**	**Control vs Powder**	**Control vs Cubes**	**Inulin vs Powder**	**Inulin vs Cubes**	**Powder vs Cubes**
**Gas production (kPa)**	0	10.87 ± 0.8	11.33 ± 2.07	10.03 ± 0.56	9.3 ± 0.76	$F(3, 4.26) = 0.61$ $p = 0.643$	n.a.	n.a.	n.a.	n.a.	n.a.	n.a.
	6	20.27 ± 0.89	28.8 ± 1.22	22.23 ± 0.47	28.47 ± 0.94	$F(3, 4.15) = 16.95$ $p = 0.009$	$p_{adj} = 0.019$	$p_{adj} = 0.173$	$p_{adj} = 0.019$	$p_{adj} = 0.033$	$p_{adj} = 0.84$	$p_{adj} = 0.02$
	*6**	*15.07 ± 0.89*	*26.27 ± 1.07*	*17.8 ± 0.62*	*27.33 ± 0.97*	*n.a.*	n.a.	n.a.	n.a.	n.a.	n.a.	n.a.
	Δ0-6	9.4 ± 0.12	17.47 ± 0.88	12.2 ± 0.15	19.17 ± 0.19	$F(3, 4.21) = 514.41$ $p = 0.001$	$p_{adj} = 0.016$	$p_{adj} < 0.001$	$p_{adj} < 0.001$	$p_{adj} = 0.029$	$p_{adj} = 0.19$	$p_{adj} < 0.001$
	24	39.3 ± 1.01	109.6 ± 1.21	76.67 ± 1.31	96.53 ± 0.72	$F(3, 4.33) = 690.65$ $p = 0.001$	$p_{adj} < 0.001$	$p_{adj} < 0.001$	$p_{adj} < 0.001$	$p_{adj} < 0.001$	$p_{adj} = 0.002$	$p_{adj} = 0.001$
	*24**	*32.37 ± 0.7*	*98.4 ± 0.78*	*67.1 ± 1.26*	*86 ± 1.04*	*n.a.*	*n.a.*	*n.a.*	*n.a.*	*n.a.*	*n.a.*	*n.a.*
	Δ6-24	24.23 ± 0.14	83.33 ± 0.14	58.87 ± 1.15	69.2 ± 0.26	$F(3, 4.19) = 21660.24$ $p = 0.001$	$p_{adj} < 0.001$	$p_{adj} = 0.001$	$p_{adj} < 0.001$	$p_{adj} = 0.002$	$p_{adj} < 0.001$	$p_{adj} = 0.009$
	Δ0-24	*33.63 ± 0.22*	*100.8 ± 0.79*	*71.07 ± 1.27*	*88.37 ± 0.13*	*$F(3, 3.89) = 11631.29$ $p = 0.001$*	$p_{adj} < 0.001$	$p_{adj} = 0.001$	$p_{adj} < 0.001$	$p_{adj} < 0.001$	$p_{adj} = 0.004$	$p_{adj} = 0.005$
	48	32.23 ± 0.79	106.53 ± 0.58	60.77 ± 1.19	96.67 ± 1.16	$F(3, 4.27) = 1591.21$ $p = 0.001$	$p_{adj} < 0.001$	$p_{adj} < 0.001$	$p_{adj} < 0.001$	$p_{adj} < 0.001$	$p_{adj} = 0.005$	$p_{adj} < 0.001$
	Δ24-48	-0.13 ± 0.18	8.13 ± 0.52	-6.33 ± 0.38	10.67 ± 0.12	$F(3, 4.05) = 937.9$ $p = 0.001$	$p_{adj} = 0.002$	$p_{adj} = 0.001$	$p_{adj} < 0.001$	$p_{adj} < 0.001$	$p_{adj} = 0.034$	$p_{adj} < 0.001$
	Δ0-48	*33.5 ± 0.36*	*108.93 ± 1*	*64.73 ± 1.13*	*99.03 ± 0.2*	*$F(3, 3.89) = 6457.69$ $p = 0.001$*	$p_{adj} < 0.001$	$p_{adj} = 0.001$	$p_{adj} < 0.001$	$p_{adj} < 0.001$	$p_{adj} = 0.008$	$p_{adj} = 0.001$

	Time (h)	Control	Inulin	Powder	Cubes	robust ANOVA	pairwise comparisons *(adjusted p-value)* Control vs Inulin	Control vs Powder	Control vs Cubes	Inulin vs Powder	Inulin vs Cubes	Powder vs Cubes
**Butyrate (mM)**	0	0.1 ± 0	0.1 ± 0	0.1 ± 0	0.1 ± 0	$F(3, 4.08) = 0.61$ $p = 0.648$	n.a.	n.a.	n.a.	n.a.	n.a.	n.a.
	6	0.18 ± 0.02	0.16 ± 0	0.16 ± 0	0.16 ± 0	$F(3, 4.02) = 0.78$ $p = 0.566$	n.a.	n.a.	n.a.	n.a.	n.a.	n.a.
	Δ0-6	0.08 ± 0.02	0.06 ± 0	0.06 ± 0	0.06 ± 0	$F(3, 4.24) = 0.58$ $p = 0.662$	n.a.	n.a.	n.a.	n.a.	n.a.	n.a.
	24	2.05 ± 0.01	1.37 ± 0.01	1.99 ± 0.04	2.09 ± 0.05	$F(3, 3.9) = 491.9$ $p = 0.001$	$p_{adj} < 0.001$	$p_{adj} = 0.346$	$p_{adj} = 0.52$	$p_{adj} = 0.008$	$p_{adj} = 0.008$	$p_{adj} = 0.299$
	Δ6-24	1.87 ± 0.03	1.21 ± 0.01	1.83 ± 0.05	1.93 ± 0.05	$F(3, 3.66) = 169.13$ $p = 0.001$	$p_{adj} = 0.007$	$p_{adj} = 0.523$	$p_{adj} = 0.435$	$p_{adj} = 0.008$	$p_{adj} = 0.008$	$p_{adj} = 0.31$
	Δ0-24	1.95 ± 0.01	1.26 ± 0.01	1.88 ± 0.04	1.99 ± 0.05	$F(3, 3.88) = 640.4$ $p = 0.001$	$p_{adj} < 0.001$	$p_{adj} = 0.353$	$p_{adj} = 0.493$	$p_{adj} = 0.009$	$p_{adj} = 0.009$	$p_{adj} = 0.287$
	48	3.67 ± 0.18	4.21 ± 0.11	4.76 ± 0.14	5.22 ± 0.21	$F(3, 4.32) = 10.45$ $p = 0.02$	$p_{adj} = 0.094$	$p_{adj} = 0.031$	$p_{adj} = 0.031$	$p_{adj} = 0.057$	$p_{adj} = 0.049$	$p_{adj} = 0.155$
	Δ24-48	1.62 ± 0.17	2.84 ± 0.1	2.77 ± 0.12	3.13 ± 0.16	$F(3, 4.35) = 12.17$ $p = 0.015$	$p_{adj} = 0.016$	$p_{adj} = 0.016$	$p_{adj} = 0.016$	$p_{adj} = 0.67$	$p_{adj} = 0.265$	$p_{adj} = 0.233$
	Δ0-48	3.57 ± 0.18	4.11 ± 0.11	4.66 ± 0.14	5.12 ± 0.21	$F(3, 4.32) = 10.58$ $p = 0.02$	$p_{adj} = 0.09$	$p_{adj} = 0.03$	$p_{adj} = 0.03$	$p_{adj} = 0.058$	$p_{adj} = 0.048$	$p_{adj} = 0.153$

	Time (h)	Control	Inulin	Powder	Cubes	robust ANOVA	pairwise comparisons *(adjusted p-value)* Control vs Inulin	Control vs Powder	Control vs Cubes	Inulin vs Powder	Inulin vs Cubes	Powder vs Cubes
**Propionate (mM)**	0	0.05 ± 0.05	0 ± 0	0 ± 0	0 ± 0	n.a.	n.a.	n.a.	n.a.	n.a.	n.a.	n.a.
	6	3.06 ± 0.01	5.09 ± 0.07	5.31 ± 0.06	5.79 ± 0.09	$F(3, 3.52) = 635.94$<br>$p = 0.001$	$p_{adj} = 0.002$	$p_{adj} = 0.002$	$p_{adj} = 0.002$	$p_{adj} = 0.089$	$p_{adj} = 0.007$	$p_{adj} = 0.02$
	Δ0-6	3.01 ± 0.04	5.09 ± 0.07	5.31 ± 0.06	5.79 ± 0.09	$F(3, 4.19) = 430.7$<br>$p = 0.001$	$p_{adj} < 0.001$	$p_{adj} < 0.001$	$p_{adj} < 0.001$	$p_{adj} = 0.089$	$p_{adj} = 0.007$	$p_{adj} = 0.02$
	24	8.33 ± 0.09	21.41 ± 0.16	19.97 ± 0.23	18.61 ± 0.48	$F(3, 4.03) = 1617.34$<br>$p = 0.001$	$p_{adj} < 0.001$	$p_{adj} < 0.001$	$p_{adj} = 0.003$	$p_{adj} = 0.015$	$p_{adj} = 0.025$	$p_{adj} = 0.09$
	Δ6-24	5.27 ± 0.1	16.32 ± 0.16	14.66 ± 0.29	12.82 ± 0.44	$F(3, 4.05) = 979.06$<br>$p = 0.001$	$p_{adj} < 0.001$	$p_{adj} = 0.001$	$p_{adj} = 0.005$	$p_{adj} = 0.017$	$p_{adj} = 0.013$	$p_{adj} = 0.032$
	Δ0-24	8.28 ± 0.14	21.41 ± 0.16	19.97 ± 0.23	18.61 ± 0.48	$F(3, 4.25) = 1155.61$<br>$p = 0.001$	$p_{adj} < 0.001$	$p_{adj} < 0.001$	$p_{adj} = 0.002$	$p_{adj} = 0.015$	$p_{adj} = 0.025$	$p_{adj} = 0.09$
	48	10.09 ± 0.51	24.89 ± 0.95	22.14 ± 0.63	21.08 ± 1.06	$F(3, 4.3) = 84.63$<br>$p = 0.001$	$p_{adj} = 0.002$	$p_{adj} = 0.001$	$p_{adj} = 0.006$	$p_{adj} = 0.099$	$p_{adj} = 0.084$	$p_{adj} = 0.451$
	Δ24-48	1.76 ± 0.42	3.48 ± 0.91	2.16 ± 0.64	2.47 ± 0.58	$F(3, 4.32) = 0.83$<br>$p = 0.542$	n.a.	n.a.	n.a.	n.a.	n.a.	n.a.
	Δ0-48	10.04 ± 0.55	24.89 ± 0.95	22.14 ± 0.63	21.08 ± 1.06	$F(3, 4.32) = 79.45$<br>$p = 0.001$	$p_{adj} = 0.002$	$p_{adj} = 0.001$	$p_{adj} = 0.005$	$p_{adj} = 0.099$	$p_{adj} = 0.084$	$p_{adj} = 0.451$

	Time (h)	Control	Inulin	Powder	Cubes	robust ANOVA	pairwise comparisons *(adjusted p-value)* Control vs Inulin	Control vs Powder	Control vs Cubes	Inulin vs Powder	Inulin vs Cubes	Powder vs Cubes
**Acetate (mM)**	0	0.41 ± 0.21	0 ± 0	0 ± 0	0.43 ± 0.21	n.a.	n.a.	n.a.	n.a.	n.a.	n.a.	n.a.
	6	5.52 ± 0.04	8.71 ± 0.09	8.7 ± 0.05	9.08 ± 0.07	$F(3, 4.27) = 971$ $p = 0.001$	$p_{adj} < 0.001$	$p_{adj} < 0.001$	$p_{adj} < 0.001$	$p_{adj} = 0.944$	$p_{adj} = 0.049$	$p_{adj} = 0.027$
	Δ0-6	5.11 ± 0.22	8.71 ± 0.09	8.7 ± 0.05	8.65 ± 0.15	$F(3, 3.92) = 66.29$ $p = 0.001$	$p_{adj} = 0.003$	$p_{adj} = 0.005$	$p_{adj} = 0.002$	$p_{adj} = 0.944$	$p_{adj} = 0.918$	$p_{adj} = 0.918$
	24	17.75 ± 0.48	30.07 ± 0.21	31.32 ± 0.5	30.32 ± 0.9	$F(3, 3.95) = 146.27$ $p = 0.001$	$p_{adj} = 0.001$	$p_{adj} < 0.001$	$p_{adj} = 0.002$	$p_{adj} = 0.176$	$p_{adj} = 0.804$	$p_{adj} = 0.484$
	Δ6-24	12.22 ± 0.44	21.35 ± 0.17	22.61 ± 0.48	21.25 ± 0.86	$F(3, 3.87) = 96.72$ $p = 0.001$	$p_{adj} = 0.002$	$p_{adj} = 0.001$	$p_{adj} = 0.005$	$p_{adj} = 0.163$	$p_{adj} = 0.912$	$p_{adj} = 0.305$
	Δ0-24	17.33 ± 0.55	30.07 ± 0.21	31.32 ± 0.5	29.9 ± 0.72	$F(3, 3.95) = 123.96$ $p = 0.001$	$p_{adj} = 0.001$	$p_{adj} < 0.001$	$p_{adj} = 0.001$	$p_{adj} = 0.176$	$p_{adj} = 0.841$	$p_{adj} = 0.229$
	48	20.62 ± 0.17	34.9 ± 1.56	36.2 ± 0.97	36.24 ± 1.77	$F(3, 3.45) = 96.3$ $p = 0.001$	$p_{adj} = 0.024$	$p_{adj} = 0.018$	$p_{adj} = 0.024$	$p_{adj} = 0.72$	$p_{adj} = 0.72$	$p_{adj} = 0.985$
	Δ24-48	2.88 ± 0.58	4.83 ± 1.49	4.88 ± 0.96	5.91 ± 1.04	$F(3, 4.22) = 2.1$ $p = 0.239$	n.a.	n.a.	n.a.	n.a.	n.a.	n.a.
	Δ0-48	20.21 ± 0.08	34.9 ± 1.56	36.2 ± 0.97	35.81 ± 1.66	$F(3, 3.36) = 106.35$ $p = 0.001$	$p_{adj} = 0.022$	$p_{adj} = 0.021$	$p_{adj} = 0.022$	$p_{adj} = 0.787$	$p_{adj} = 0.849$	$p_{adj} = 0.853$

	Time (h)	Control	Inulin	Powder	Cubes	robust ANOVA	pairwise comparisons *(adjusted p-value)*					
							Control vs Inulin	Control vs Powder	Control vs Cubes	Inulin vs Powder	Inulin vs Cubes	Powder vs Cubes
**Total SCFA (mM)**	0	0.57 ± 0.23	0.1 ± 0	0.1 ± 0	0.53 ± 0.21	$F(3, 3.65) = 2.39$ $p = 0.222$	n.a.	n.a.	n.a.	n.a.	n.a.	n.a.
	6	8.83 ± 0.02	13.96 ± 0.16	14.18 ± 0.08	15.06 ± 0.19	$F(3, 3.51) = 1408.13$ $p = 0.001$	$p_{adj} = 0.002$	$p_{adj} = 0.001$	$p_{adj} = 0.002$	$p_{adj} = 0.33$	$p_{adj} = 0.018$	$p_{adj} = 0.033$
	Δ0-6	8.26 ± 0.22	13.86 ± 0.17	14.08 ± 0.08	14.53 ± 0.07	$F(3, 4.21) = 181.47$ $p = 0.001$	$p_{adj} < 0.001$	$p_{adj} = 0.001$	$p_{adj} = 0.001$	$p_{adj} = 0.337$	$p_{adj} = 0.048$	$p_{adj} = 0.024$
	24	28.81 ± 0.62	53.05 ± 0.39	53.58 ± 0.79	51.34 ± 1.41	$F(3, 4.14) = 300.87$ $p = 0.001$	$p_{adj} < 0.001$	$p_{adj} < 0.001$	$p_{adj} = 0.002$	$p_{adj} = 0.588$	$p_{adj} = 0.418$	$p_{adj} = 0.381$
	Δ6-24	19.99 ± 0.6	39.08 ± 0.35	39.4 ± 0.83	36.28 ± 1.31	$F(3, 4.08) = 197.72$ $p = 0.001$	$p_{adj} < 0.001$	$p_{adj} < 0.001$	$p_{adj} = 0.004$	$p_{adj} = 0.747$	$p_{adj} = 0.191$	$p_{adj} = 0.191$
	Δ0-24	28.25 ± 0.72	52.95 ± 0.39	53.48 ± 0.79	50.81 ± 1.24	$F(3, 4.11) = 244.14$ $p = 0.001$	$p_{adj} < 0.001$	$p_{adj} < 0.001$	$p_{adj} = 0.001$	$p_{adj} = 0.589$	$p_{adj} = 0.266$	$p_{adj} = 0.235$
	48	36.26 ± 1.41	64.4 ± 2.63	64.84 ± 1.68	63.49 ± 3.02	$F(3, 4.29) = 55.99$ $p = 0.001$	$p_{adj} = 0.007$	$p_{adj} = 0.001$	$p_{adj} = 0.009$	$p_{adj} = 0.896$	$p_{adj} = 0.896$	$p_{adj} = 0.896$
	Δ24-48	7.45 ± 0.95	11.35 ± 2.53	11.26 ± 1.68	12.15 ± 1.76	$F(3, 4.18) = 2.13$ $p = 0.235$	n.a.	n.a.	n.a.	n.a.	n.a.	n.a.
	Δ0-48	35.7 ± 1.36	64.3 ± 2.63	64.74 ± 1.68	62.96 ± 2.9	$F(3, 4.28) = 59.91$ $p = 0.001$	$p_{adj} = 0.007$	$p_{adj} = 0.001$	$p_{adj} = 0.008$	$p_{adj} = 0.896$	$p_{adj} = 0.896$	$p_{adj} = 0.896$

	Time (h)	Control	Inulin	Powder	Cubes	robust ANOVA	pairwise comparisons *(adjusted p-value)* Control vs Inulin	Control vs Powder	Control vs Cubes	Inulin vs Powder	Inulin vs Cubes	Powder vs Cubes
**Branched SCFA (mM)**	0	0 ± 0	0 ± 0	0 ± 0	0 ± 0	n.a.	n.a.	n.a.	n.a.	n.a.	n.a.	n.a.
	6	0.06 ± 0	0 ± 0	0 ± 0	0.03 ± 0.03	n.a.	n.a.	n.a.	n.a.	n.a.	n.a.	n.a.
	Δ0-6	0.06 ± 0	0 ± 0	0 ± 0	0.03 ± 0.03	n.a.	n.a.	n.a.	n.a.	n.a.	n.a.	n.a.
	24	0.69 ± 0.03	0.2 ± 0.03	0.31 ± 0.01	0.31 ± 0.02	$F(3, 3.85) = 36.93$ $p = 0.003$	$p_{adj} = 0.002$	$p_{adj} = 0.009$	$p_{adj} = 0.004$	$p_{adj} = 0.069$	$p_{adj} = 0.069$	$p_{adj} = 0.997$
	Δ6-24	0.63 ± 0.03	0.2 ± 0.03	0.31 ± 0.01	0.28 ± 0.01	$F(3, 4) = 34.64$ $p = 0.003$	$p_{adj} = 0.003$	$p_{adj} = 0.012$	$p_{adj} = 0.012$	$p_{adj} = 0.081$	$p_{adj} = 0.106$	$p_{adj} = 0.081$
	Δ0-24	0.69 ± 0.03	0.2 ± 0.03	0.31 ± 0.01	0.31 ± 0.02	$F(3, 3.85) = 36.93$ $p = 0.003$	$p_{adj} = 0.002$	$p_{adj} = 0.009$	$p_{adj} = 0.004$	$p_{adj} = 0.069$	$p_{adj} = 0.069$	$p_{adj} = 0.997$
	48	1.88 ± 0.71	0.41 ± 0.01	1.65 ± 0.06	0.95 ± 0.09	$F(3, 3.42) = 116.46$ $p = 0.001$	$p_{adj} = 0.263$	$p_{adj} = 0.779$	$p_{adj} = 0.387$	$p_{adj} = 0.01$	$p_{adj} = 0.055$	$p_{adj} = 0.018$
	Δ24-48	1.19 ± 0.68	0.2 ± 0.03	1.34 ± 0.06	0.64 ± 0.11	$F(3, 3.83) = 67$ $p = 0.001$	$p_{adj} = 0.429$	$p_{adj} = 0.844$	$p_{adj} = 0.611$	$p_{adj} = 0.004$	$p_{adj} = 0.101$	$p_{adj} = 0.032$
	Δ0-48	1.88 ± 0.71	0.41 ± 0.01	1.65 ± 0.06	0.95 ± 0.09	$F(3, 3.42) = 116.46$ $p = 0.001$	$p_{adj} = 0.263$	$p_{adj} = 0.779$	$p_{adj} = 0.387$	$p_{adj} = 0.01$	$p_{adj} = 0.055$	$p_{adj} = 0.018$

	Time (h)	Control	Inulin	Powder	Cubes	robust ANOVA	pairwise comparisons *(adjusted p-value)* Control vs Inulin	Control vs Powder	Control vs Cubes	Inulin vs Powder	Inulin vs Cubes	Powder vs Cubes
**Lactate (mM)**	0	0.13 ± 0.02	0.26 ± 0.01	0.17 ± 0	0.18 ± 0	$F(3, 3.67) = 18.18$ $p = 0.012$	$p_{adj} = 0.032$	$p_{adj} = 0.194$	$p_{adj} = 0.164$	$p_{adj} = 0.025$	$p_{adj} = 0.032$	$p_{adj} = 0.164$
	6	0.64 ± 0.01	3.5 ± 0.1	2.63 ± 0.04	4.28 ± 0.09	$F(3, 3.49) = 996.82$ $p = 0.001$	$p_{adj} = 0.002$	$p_{adj} = 0.001$	$p_{adj} = 0.001$	$p_{adj} = 0.007$	$p_{adj} = 0.006$	$p_{adj} = 0.001$
	Δ0-6	0.51 ± 0.03	3.24 ± 0.1	2.46 ± 0.05	4.1 ± 0.09	$F(3, 4.07) = 650.6$ $p = 0.001$	$p_{adj} = 0.001$	$p_{adj} < 0.001$	$p_{adj} = 0.001$	$p_{adj} = 0.008$	$p_{adj} = 0.004$	$p_{adj} = 0.001$
	24	0.37 ± 0.01	3.16 ± 0.12	1.91 ± 0.06	2.89 ± 0.1	$F(3, 3.45) = 401.28$ $p = 0.001$	$p_{adj} = 0.004$	$p_{adj} = 0.004$	$p_{adj} = 0.004$	$p_{adj} = 0.004$	$p_{adj} = 0.164$	$p_{adj} = 0.004$
	Δ6-24	-0.27 ± 0.01	-0.34 ± 0.18	-0.72 ± 0.02	-1.39 ± 0.18	$F(3, 3.56) = 164.22$ $p = 0.001$	$p_{adj} = 0.758$	$p_{adj} = 0.003$	$p_{adj} = 0.049$	$p_{adj} = 0.186$	$p_{adj} = 0.041$	$p_{adj} = 0.096$
	Δ0-24	0.24 ± 0.03	2.91 ± 0.12	1.73 ± 0.06	2.7 ± 0.1	$F(3, 3.85) = 320.26$ $p = 0.001$	$p_{adj} = 0.002$	$p_{adj} = 0.002$	$p_{adj} = 0.002$	$p_{adj} = 0.004$	$p_{adj} = 0.258$	$p_{adj} = 0.004$
	48	0 ± 0	0.28 ± 0.02	0.29 ± 0.01	0.29 ± 0	$F(3, 3.55) = 1473.72$ $p = 0.001$	$p_{adj} = 0.007$	$p_{adj} = 0.001$	$p_{adj} < 0.001$	$p_{adj} = 0.587$	$p_{adj} = 0.587$	$p_{adj} = 0.709$
	Δ24-48	-0.36 ± 0.01	-2.89 ± 0.12	-1.61 ± 0.05	-2.6 ± 0.1	$F(3, 3.44) = 335.32$ $p = 0.001$	$p_{adj} = 0.004$	$p_{adj} = 0.004$	$p_{adj} = 0.004$	$p_{adj} = 0.004$	$p_{adj} = 0.137$	$p_{adj} = 0.004$
	Δ0-48	-0.13 ± 0.02	0.02 ± 0.01	0.12 ± 0.01	0.11 ± 0	$F(3, 3.88) = 39.13$ $p = 0.003$	$p_{adj} = 0.011$	$p_{adj} = 0.011$	$p_{adj} = 0.011$	$p_{adj} = 0.011$	$p_{adj} = 0.019$	$p_{adj} = 0.23$

	Time (h)	Control	Inulin	Powder	Cubes	robust ANOVA	pairwise comparisons *(adjusted p-value)* Control vs Inulin	Control vs Powder	Control vs Cubes	Inulin vs Powder	Inulin vs Cubes	Powder vs Cubes
**Ammonium (mg/L)**	0	64.99 ± 8.37	90.3 ± 2.52	67.61 ± 1.89	86.38 ± 1.26	$F(3, 4.12) = 21.37$ $p = 0.006$	$p_{adj} = 0.167$	$p_{adj} = 0.787$	$p_{adj} = 0.183$	$p_{adj} = 0.008$	$p_{adj} = 0.311$	$p_{adj} = 0.008$
	24	297.32 ± 3.64	179.01 ± 8.19	192.08 ± 6.88	229.99 ± 4.9	$F(3, 4.27) = 83.8$ $p = 0.001$	$p_{adj} = 0.003$	$p_{adj} = 0.002$	$p_{adj} = 0.002$	$p_{adj} = 0.291$	$p_{adj} = 0.015$	$p_{adj} = 0.017$
	Δ0-24	232.32 ± 10.07	88.71 ± 8.95	124.47 ± 8.04	143.61 ± 5.94	$F(3, 4.36) = 30.81$ $p = 0.003$	$p_{adj} = 0.003$	$p_{adj} = 0.004$	$p_{adj} = 0.007$	$p_{adj} = 0.05$	$p_{adj} = 0.015$	$p_{adj} = 0.134$
	48	391.53 ± 5.29	222.61 ± 6.31	249.22 ± 12.75	271.17 ± 6.6	$F(3, 4.34) = 123.22$ $p = 0.001$	$p_{adj} < 0.001$	$p_{adj} = 0.006$	$p_{adj} = 0.001$	$p_{adj} = 0.193$	$p_{adj} = 0.009$	$p_{adj} = 0.224$
	Δ0-48	326.54 ± 10.16	132.32 ± 7.96	181.62 ± 12.62	184.79 ± 7.38	$F(3, 4.38) = 59.42$ $p = 0.001$	$p_{adj} = 0.001$	$p_{adj} = 0.002$	$p_{adj} = 0.002$	$p_{adj} = 0.046$	$p_{adj} = 0.013$	$p_{adj} = 0.841$
	Δ24-48	94.22 ± 8.79	43.6 ± 3.48	57.15 ± 8.67	41.18 ± 8.42	$F(3, 4.02) = 7.63$ $p = 0.04$	$p_{adj} = 0.054$	$p_{adj} = 0.08$	$p_{adj} = 0.054$	$p_{adj} = 0.308$	$p_{adj} = 0.809$	$p_{adj} = 0.308$

***Ex vivo* gut permeability assessment: changes in gut microbiota composition, *in vitro* fermentation metabolites and gut integrity and permeability**

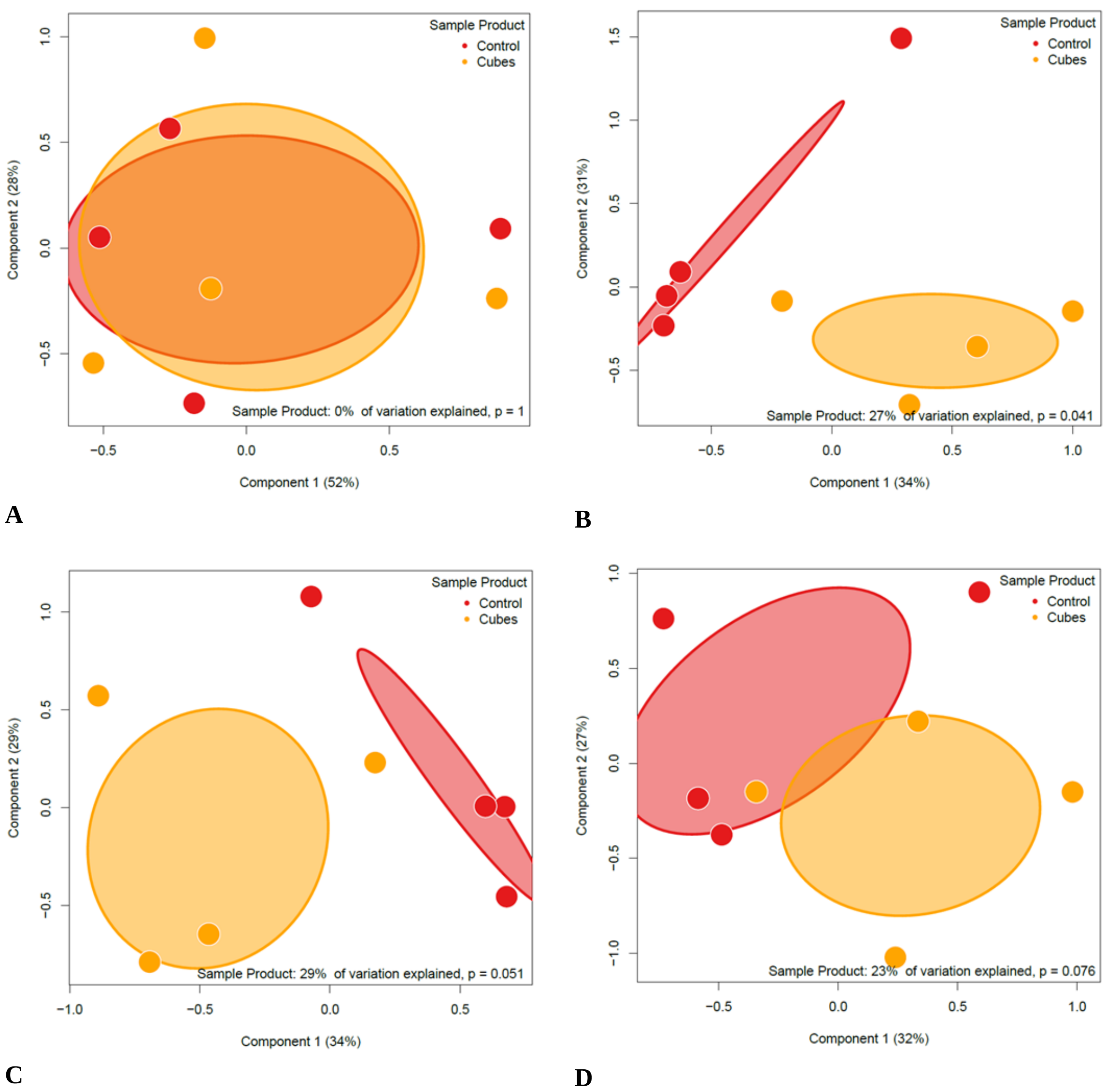

Supplementary Figure 9. Analysis of gut microbiota composition. A-C: Principal coordinate analysis based on Bray-Curtis dissimilarity to assess differences in overall gut microbiota composition (β-diversity representing between sample differences) at each timepoint (0 h, 6 h, 24 h and 48 h) between control fermentation (Control; only inoculum) or *in vitro* fermentation of dried chicory root cubes (Cubes). A: baseline (t = 0 h); B: after 6 h of in vitro fermentation; C: after 24 h of in vitro fermentation; and D: after 48 h of in vitro fermentation.

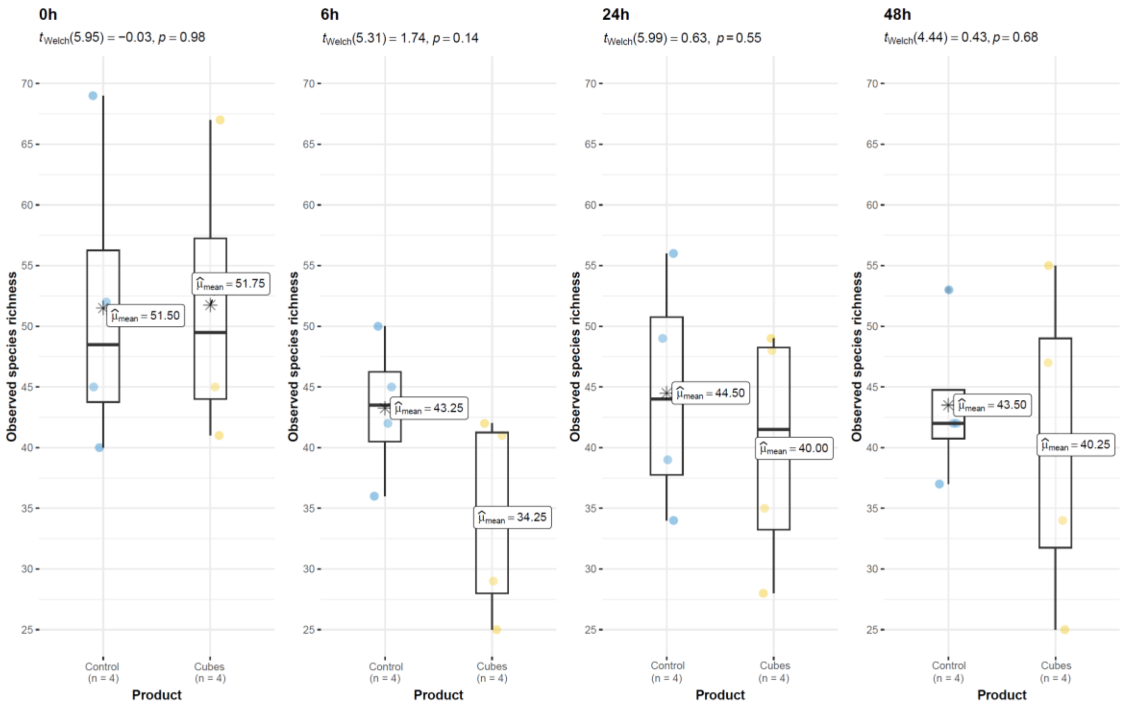

Supplementary Figure 10. Alpha-diversity assessed by observed gut bacteria richness (mean richness within each sample) at baseline (t = 0 h), 6 h, 24 h and 48 h *in vitro* fermentation of the control (Control; only inoculum) and dried chicory root cubes (Cubes). Statistical details for unpaired t-test with unequal variances and corresponding p-value are given above the plot.

Besides statistically significantly different changes between control and dried chicory root cube fermentations in *Bifidobacterium* spp., we also observed differences for changes in *Butyricicoccus* spp levels at 24 h (p = 0.074, q = 0.356) and 48h (p = 0.034, q = 0.181) and *Coprococcus* spp. at 6 h (p = 0.003, q = 0.076), 24 h (p = 0.021, q = 0.356) and 48 h (p = 0.048, q = 0.216). We observed a number of other genera changing over time and (Supplementary Table 5) and differing between timepoints (Supplementary Table 6) for control versus dried chicory root cube fermentation. *Lachnospira* spp. decreased at 6 h in both groups but re-increased for dried chicory root cubes at 24 and 48 h. Also, changes in an unidentified genus of *Oscillospiraceae* differed, which decreased in both groups, but more pronounced in for dried chicory root cubes. Taxa that decrease in both conditions were (ordered by decreasing mean relative levels) *Blautia* spp, an unidentified *Lachnospiraceae*, *Faecalibacterium* spp., *Anaerostipes* spp, and *Lachnospiraceae* ND3007 group, while *Sutterella* spp. levels increased in both

conditions. Both these decreases and increases occurred rapidly within 6 h and plateaued at 24 h. Several other genera decreased only in one of the conditions. For dried chicory root cubes these were decreases in an unidentified *Clostridia UCG-014* genus, *Dorea* spp, *Coriobacteriales* Incertae Sedis, *Romboutsia* spp., *Ruminococcus gauvreauii* group (Supplementary Table X). For the control condition decreasing genera were *Roseburia* spp. and *Subdoligranulum* spp. and increasing genera *Lachnospiraceae UCG-004, Parabacteroides* spp. and *Senegalimassilia* spp. Comparing mean relative levels at each timepoint, we observed at 24 h in dried chicory root cubes compared to control fermentations statistically significantly higher *Butyricoccus* spp. and lower *Coproccoccus* spp., lower *Lachnospiraceae UCG-004* and lower *Erysipelotrichaceae_UCG.003.*At 48 h we observed in dried chicory root cubes compared to control fermentations statistically significantly higher *Bifidobacterium* spp. and higher *Lachnospira* spp.*,* lower *Coprococcus* spp.*,* lower unidentified genus of *Oscillospiraceae* and lower *Eubacterium hallii* group.

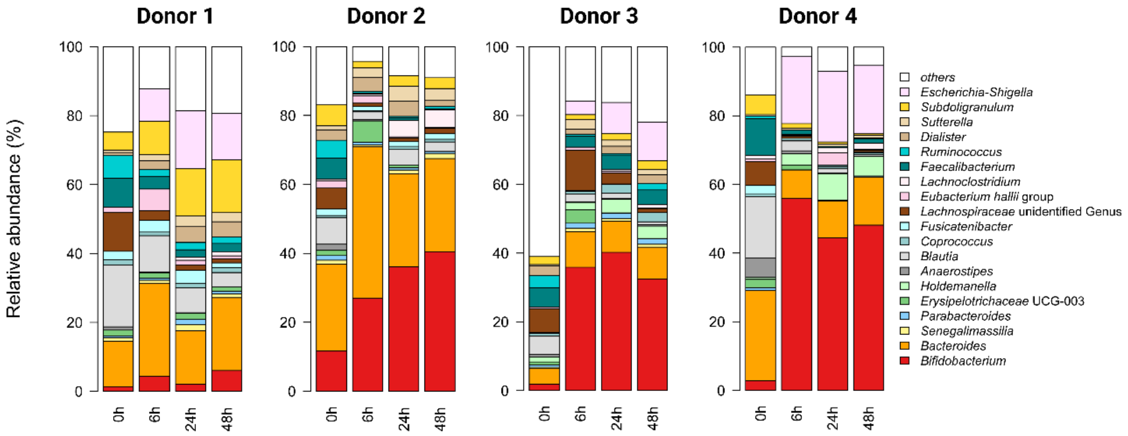

A

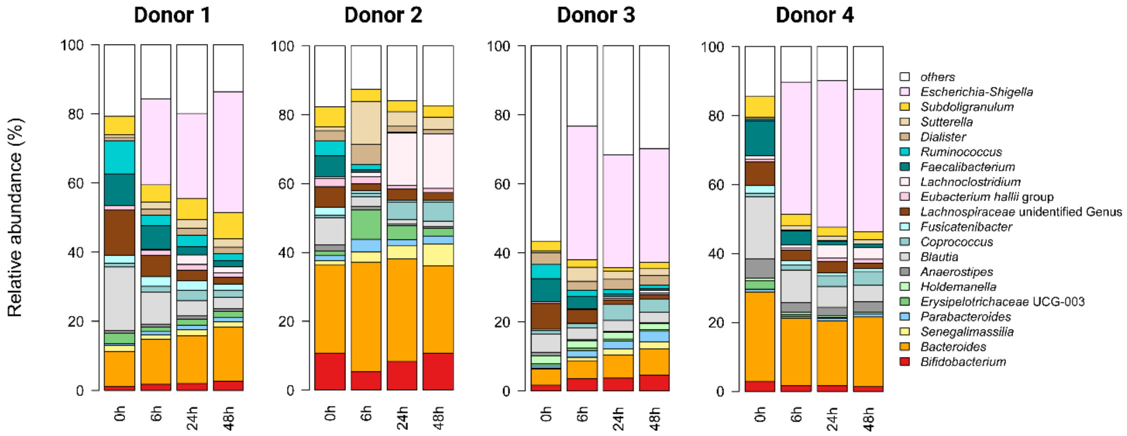

B

Supplementary Figure 11. Analysis of individual gut microbiota composition. Common genera present with a mean relative abundance of at least 1% and mean prevalence of 50% in the whole dataset at each timepoint in each of the *in vitro f*ermentation. A: genera present in each donor during *in vitro* fermentation of dried chicory root cubes. B: genera present in each donor during in the control fermentation.

Supplementary Table 5. Changes in mean relative abundances of common genera (mean relative abundance of at least 1% and mean prevalence of 50% in the whole dataset) during the fermentation of dried chicory root cubes (CUBES) or negative control (CONTROL; only inoculum) from baseline (t = 0 h) to 6 h, 24 h and 48 h (used for Ussing experiment). Respective fold-changes (fold-Δ) over time within each group and the p-values and fdr-corrected q-values are given per taxon for each fermentation.

Taxon	Product	0h	6h	24h	48h	0-6h	0-24h	0-48h	0-6h	0-24h	0-48h	0-6h	0-24h	0-48h
		**Relative abundance (%)**				**Fold-Δ over time**			**p-value**			**q-value**		
Alistipes	CUBES	1.02%	0.30%	0.73%	0.94%	0.30	0.71	0.92	0.070	0.616	0.901	0.166	0.849	0.987
	CONTROL	1.06%	1.10%	1.06%	1.37%	1.04	1.00	1.29	0.299	0.299	0.487	0.722	0.559	0.640
Anaerostipes	CUBES	2.39%	0.45%	0.15%	0.10%	0.19	0.06	0.04	0.196	**0.031**	**0.013**	0.313	0.112	**0.049**
	CONTROL	2.52%	1.30%	1.08%	1.21%	0.52	0.43	0.48	**<0.001**	**<0.001**	**<0.001**	**0.005**	**<0.001**	**<0.001**
Bacteroides	CUBES	18.69%	22.97%	16.58%	19.01%	1.23	0.89	1.02	0.591	0.515	0.979	0.739	0.736	1.000
	CONTROL	17.76%	18.15%	18.66%	18.63%	1.02	1.05	1.05	0.958	0.508	0.265	1.000	0.743	0.486
Bifidobacterium	CUBES	4.90%	32.45%	33.55%	34.44%	6.62	6.84	7.03	**0.019**	0.092	**0.033**	0.051	0.283	0.094
	CONTROL	4.46%	3.34%	4.38%	5.41%	0.75	0.98	1.21	0.297	0.716	0.805	0.722	0.860	0.917
Bilophila	CUBES	0.10%	0.03%	0.09%	0.10%	0.29	0.86	1.03	0.310	0.992	0.355	0.427	1.000	0.546
	CONTROL	0.15%	0.16%	0.35%	0.44%	1.08	2.26	2.86	0.715	0.279	0.138	0.890	0.559	0.334
Blautia	CUBES	13.59%	4.62%	3.86%	2.26%	0.34	0.28	0.17	**<0.001**	**<0.001**	**<0.001**	**<0.001**	**<0.001**	**<0.001**
	CONTROL	13.49%	6.53%	4.03%	3.40%	0.48	0.30	0.25	**<0.001**	**<0.001**	**<0.001**	**<0.001**	**<0.001**	**<0.001**

Taxon	Product	0h	6h	24h	48h	0-6h	0-24h	0-48h	0-6h	0-24h	0-48h	0-6h	0-24h	0-48h
		**Relative abundance (%)**				**Fold-Δ over time**			**p-value**			**q-value**		
Butyricicoccus	CUBES	0.22%	0.03%	0.26%	0.17%	0.15	1.18	0.77	0.114	0.888	0.829	0.217	0.946	0.987
	CONTROL	0.17%	0.10%	0.09%	0.00%	0.56	0.53	0.00	0.786	0.763	1.000	0.920	0.860	1.000
Lachnospiraceae CAG.56	CUBES	0.79%	0.33%	0.27%	0.11%	0.42	0.34	0.14	0.590	0.197	0.093	0.739	0.414	0.233
	CONTROL	0.80%	0.41%	0.05%	0.00%	0.51	0.07	0.00	0.761	0.224	0.996	0.918	0.509	1.000
Clostridia UCG-014 unidentified Genus	CUBES	0.98%	0.09%	0.13%	0.21%	0.09	0.14	0.21	**0.005**	**0.008**	**0.014**	0.018	0.034	0.049
	CONTROL	1.21%	0.45%	0.53%	0.50%	0.37	0.44	0.41	0.582	0.240	0.179	0.851	0.518	0.367
Collinsella	CUBES	0.80%	0.79%	0.86%	0.47%	0.99	1.07	0.59	0.918	0.892	0.664	0.993	0.946	0.885
	CONTROL	0.07%	0.52%	0.25%	0.53%	7.31	3.53	7.48	0.429	0.616	0.424	0.752	0.815	0.621
Coprococcus	CUBES	1.15%	0.66%	1.47%	1.48%	0.58	1.28	1.29	<0.001	0.381	0.633	**0.001**	0.588	0.882
	CONTROL	**1.13%**	**1.42%**	**4.36%**	**4.32%**	**1.26**	**3.85**	**3.82**	0.272	**<0.001**	**<0.001**	**0.722**	**<0.001**	**<0.001**
Dialister	CUBES	2.22%	2.13%	2.94%	2.45%	0.96	1.33	1.11	0.976	0.827	0.938	1.000	0.946	0.987
	CONTROL	2.33%	2.76%	1.96%	1.69%	1.19	0.84	0.73	0.397	0.955	0.632	0.752	1.000	0.762
Dorea	CUBES	1.29%	0.75%	1.13%	0.52%	0.59	0.88	0.41	**0.006**	**0.284**	**<0.001**	**0.023**	0.473	**<0.001**
	CONTROL	1.32%	1.16%	1.85%	0.71%	0.88	1.40	0.54	0.846	0.616	0.361	0.937	0.815	0.600
Erysipelotrichaceae	CUBES	1.82%	3.41%	0.69%	0.32%	1.87	0.38	0.18	0.654	0.489	0.217	0.793	0.725	0.394

Taxon	Product	0h	6h	24h	48h	0-6h	0-24h	0-48h	0-6h	0-24h	0-48h	0-6h	0-24h	0-48h
		Relative abundance (%)				Fold-Δ over time			p-value			q-value		
UCG-003	CONTROL	2.02%	2.87%	1.86%	1.22%	1.42	0.92	0.60	0.999	0.675	0.384	1.000	0.858	0.600
Escherichia-Shigella	CUBES	0.00%	8.59%	12.64%	12.21%	Inf	Inf	Inf	1.000	1.000	1.000	1.000	1.000	1.000
	CONTROL	0.00%	27.89%	27.53%	29.90%	Inf	Inf	Inf	1.000	1.000	1.000	1.000	1.000	1.000
Faecalibacterium	CUBES	8.97%	2.20%	2.03%	2.34%	0.25	0.23	0.26	**<0.001**	**<0.001**	<0.001	<0.001	<0.001	<0.001
	CONTROL	0.00	76.10	117.00	117.00	0.00	76.10	0.10	0.383	**0.032**	**0.017**	0.752	0.110	0.059
Fusicatenibacter	CUBES	1.84%	1.35%	1.50%	0.86%	0.73	0.82	0.47	0.236	0.269	0.060	0.363	0.468	0.159
	CONTROL	1.93%	1.31%	1.08%	0.83%	0.68	0.56	0.43	0.481	0.314	0.219	0.752	0.560	0.428
Coriobacteriales Incertae Sedis	CUBES	0.45%	0.10%	0.10%	0.15%	0.22	0.22	0.32	**<0.001**	**<0.001**	**<0.001**	**<0.001**	**<0.001**	**<0.001**
	CONTROL	0.49%	0.22%	0.19%	0.29%	0.45	0.39	0.58	0.717	0.690	0.148	0.890	0.858	0.337
Lachnoclostridium	CUBES	0.40%	0.19%	2.05%	2.35%	0.48	5.19	5.93	0.591	0.229	0.193	0.739	0.457	0.386
	CONTROL	0.43%	0.67%	6.01%	5.99%	1.57	14.10	14.07	0.623	**0.025**	**0.029**	0.851	0.095	0.084
Lachnospira	CUBES	0.52%	0.04%	0.39%	0.92%	0.07	0.75	1.75	**0.010**	0.775	0.583	0.033	0.940	0.863
	CONTROL	0.49%	0.18%	0.14%	0.14%	0.37	0.29	0.29	0.488	0.387	0.395	0.752	0.618	0.600
Lachnospiraceae unidentified Genus	CUBES	9.31%	4.35%	1.57%	1.15%	0.47	0.17	0.12	**0.002**	**<0.001**	**<0.001**	**0.011**	**<0.001**	**<0.001**
	CONTROL	9.64%	4.04%	2.98%	1.83%	0.42	0.31	0.19	**<0.001**	**<0.001**	**<0.001**	**0.002**	**<0.001**	**<0.001**

Taxon	Product	0h	6h	24h	48h	0-6h	0-24h	0-48h	0-6h	0-24h	0-48h	0-6h	0-24h	0-48h
		**Relative abundance (%)**				**Fold-Δ over time**			**p-value**			**q-value**		
Lachnospiraceae FCS020 group	CUBES	0.30%	0.00%	0.00%	0.00%	0.00	0.00	0.00	NA	NA	NA	NA	NA	NA
	CONTROL	0.13%	0.04%	0.00%	0.00%	0.29	0.00	0.00	NA	NA	NA	NA	NA	NA
Lachnospiraceae ND3007 group	CUBES	2.03%	0.36%	0.40%	0.42%	0.18	0.20	0.21	**0.012**	**0.017**	**0.023**	**0.037**	0.068	0.072
	CONTROL	1.62%	0.89%	0.72%	0.61%	0.55	0.44	0.37	**0.002**	**<0.001**	**<0.001**	**0.013**	**<0.001**	**<0.001**
Lachnospiraceae NK4A136 group	CUBES	1.49%	0.29%	0.42%	0.76%	0.19	0.28	0.51	0.256	0.382	0.640	0.373	0.588	0.882
	CONTROL	1.38%	0.64%	0.45%	0.43%	0.46	0.33	0.31	0.367	0.200	0.381	0.752	0.483	0.600
Lachnospiraceae UCG-004	CUBES	0.18%	0.00%	0.56%	0.50%	0.00	3.19	2.85	1.000	0.256	0.305	1.000	0.468	0.515
	CONTROL	0.18%	0.16%	1.47%	1.23%	0.88	7.99	6.68	0.833	**0.001**	**0.003**	0.937	**0.006**	**0.015**
Monoglobus	CUBES	0.36%	0.03%	0.25%	0.45%	0.09	0.70	1.26	0.159	0.834	0.889	0.277	0.946	0.987
	CONTROL	0.32%	0.14%	0.08%	0.09%	0.44	0.25	0.27	0.488	0.179	0.168	0.752	0.483	0.362
Oscillospiraceae unidentified Genus	CUBES	0.29%	0.03%	0.18%	0.22%	0.09	0.63	0.77	0.095	0.743	0.855	0.190	0.940	0.987
	CONTROL	0.42%	0.17%	0.74%	0.62%	0.41	1.74	1.47	0.172	0.392	0.555	0.641	0.618	0.690
Parabacteroides	CUBES	0.97%	0.78%	1.07%	0.90%	0.80	1.10	0.92	0.760	0.899	0.916	0.869	0.946	0.987
	CONTROL	0.97%	1.93%	1.69%	2.13%	1.99	1.74	2.19	**0.022**	**0.025**	**0.002**	0.131	0.095	**0.013**
Romboutsia	CUBES	0.91%	0.18%	0.24%	0.29%	0.20	0.26	0.31	**<0.001**	**<0.001**	**<0.001**	**0.001**	**0.001**	**0.001**

Taxon	Product	0h	6h	24h	48h	0-6h	0-24h	0-48h	0-6h	0-24h	0-48h	0-6h	0-24h	0-48h
		**Relative abundance (%)**				**Fold-Δ over time**			**p-value**			**q-value**		
	CONTROL	1.03%	0.78%	0.82%	0.89%	0.76	0.80	0.86	0.408	0.756	0.731	0.752	0.860	0.857
Roseburia	CUBES	1.81%	0.13%	0.14%	0.38%	0.07	0.08	0.21	0.088	0.102	0.314	0.190	0.291	0.515
	CONTROL	1.80%	0.15%	0.00%	0.04%	0.08	0.00	0.02	0.093	1.000	**0.012**	0.424	1.000	**0.049**
Ruminococcus	CUBES	4.82%	0.81%	0.81%	1.18%	0.17	0.17	0.24	0.130	0.128	0.217	0.236	0.327	0.394
	CONTROL	5.38%	1.86%	1.33%	0.82%	0.35	0.25	0.15	0.286	0.087	**0.027**	0.722	0.275	0.084
Senegalimassilia	CUBES	0.69%	0.62%	0.97%	0.94%	0.90	1.40	1.36	0.836	0.131	0.131	0.929	0.327	0.290
	CONTROL	0.87%	1.41%	2.08%	2.77%	1.62	2.39	3.18	0.225	**0.017**	**0.004**	0.722	0.078	**0.016**
Streptococcus	CUBES	1.03%	0.29%	0.29%	0.31%	0.28	0.28	0.30	0.173	0.170	0.192	0.289	0.386	0.386
	CONTROL	1.10%	0.75%	0.67%	0.65%	0.68	0.61	0.59	0.389	0.161	0.272	0.752	0.472	0.486
Subdoligranulum	CUBES	5.40%	3.67%	5.10%	5.68%	0.68	0.94	1.05	**0.019**	0.079	0.128	0.051	0.262	0.290
	CONTROL	5.52%	3.66%	3.42%	4.06%	0.66	0.62	0.74	**0.011**	**0.001**	**0.017**	0.075	**0.004**	0.059
Sutterella	CUBES	0.76%	2.07%	2.62%	2.23%	2.72	3.45	2.93	**<0.001**	**<0.001**	**<0.001**	**<0.001**	**<0.001**	**<0.001**
	CONTROL	0.89%	5.20%	2.88%	2.54%	5.82	3.22	2.84	**<0.001**	**<0.001**	**<0.001**	**<0.001**	**<0.001**	**<0.001**
Oscillospiraceae UCG-002	CUBES	1.89%	0.30%	0.43%	0.72%	0.16	0.23	0.38	0.092	0.174	0.322	0.190	0.386	0.515
	CONTROL	2.17%	0.83%	1.31%	1.60%	0.38	0.60	0.74	0.167	0.200	0.499	0.641	0.483	0.640

Taxon	Product	0h	6h	24h	48h	0-6h	0-24h	0-48h	0-6h	0-24h	0-48h	0-6h	0-24h	0-48h
		**Relative abundance (%)**				**Fold-Δ over time**			**p-value**			**q-value**		
Erysipelotrichaceae UCG-003	CUBES	0.15%	0.12%	0.15%	0.12%	0.12	0.34	0.27	**0.001**	**0.003**	**0.002**	**0.006**	**0.017**	**0.010**
	CONTROL	0.27%	0.11%	0.27%	0.11%	0.35	0.53	0.21	0.644	0.776	0.489	0.852	0.860	0.640
Eubacterium coprostanoligenes group	CUBES	0.00%	0.00%	0.00%	0.00%	0.00	0.00	0.00	NA	NA	NA	NA	NA	NA
	CONTROL	0.04%	0.00%	0.04%	0.00%	0.09	0.03	0.00	NA	NA	NA	NA	NA	NA
Eubacterium eligens group	CUBES	0.17%	0.56%	0.17%	0.56%	0.17	0.33	1.06	0.063	0.265	0.923	0.157	0.468	0.987
	CONTROL	0.22%	0.10%	0.22%	0.10%	0.45	0.47	0.21	0.074	0.436	0.051	0.378	0.662	0.139
Eubacterium hallii group	CUBES	1.31%	2.55%	1.58%	0.46%	1.95	1.21	0.35	0.261	0.741	**0.020**	0.373	0.940	0.066
	CONTROL	1.36%	1.18%	1.17%	1.12%	0.86	0.86	0.82	0.253	0.616	0.481	0.722	0.815	0.640
Eubacterium ventriosum group	CUBES	0.00%	0.00%	0.00%	0.00%	0.37	0.00	0.00	NA	NA	NA	NA	NA	NA
	CONTROL	0.09%	0.00%	0.09%	0.00%	0.90	0.43	0.00	0.907	0.330	0.960	0.978	0.564	1.000
Ruminococcus gauvreauii group	CUBES	0.08%	0.08%	0.08%	0.08%	0.16	0.13	0.15	**0.001**	**0.001**	**0.001**	**0.004**	**0.003**	**0.003**
	CONTROL	0.26%	0.12%	0.26%	0.12%	0.53	0.44	0.21	0.495	0.300	0.089	0.752	0.559	0.229
Ruminococcus torques group	CUBES	1.52%	1.22%	1.52%	1.22%	1.86	1.59	1.27	0.676	0.756	0.872	0.795	0.940	0.987
	CONTROL	0.72%	0.76%	0.72%	0.76%	0.55	0.75	0.79	0.605	0.799	0.835	0.851	0.862	0.925

fold-Δ: fold-change over baseline in mean relative abundances for each condition (dried chicory root cubes fermentation versus control); NA: represents p- and q-values that could not be reliably estimated due to violation of the assumption underlying the statistical models for calculating statistical inference [15]

Supplementary Table 6. Mean relative abundances of common genera (mean relative abundance of at least 1% and mean prevalence of 50% in the whole dataset) at each timepoint (baseline 0 h, 6 h, 24 h, and 48 h) in the negative control fermentation (Control; only inoculum) or fermentation of dried chicory root cubes (Cubes) and the respective fold-difference (fold-Δ) between both conditions as well as respective p-values and fdr-corrected q-values.

	0h					6h					24h					48h				
taxon	Control (%)	Cubes (%)	Fold-Δ	p-value	q-value	Control (%)	Cubes (%)	Fold-Δ	p-value	q-value	Control (%)	Cubes (%)	Fold-Δ	p-value	q-value	Control (%)	Cubes (%)	Fold-Δ	p-value	q-value
Alistipes	1.06%	1.02%	0.96	0.783	1.000	1.10%	0.30%	0.28	0.241	0.557	1.06%	0.73%	0.69	0.529	0.731	1.37%	0.94%	0.69	0.952	0.957
Anaerostipes	2.52%	2.49%	0.99	0.998	1.000	1.30%	0.45%	0.35	0.136	0.557	1.08%	0.15%	0.14	0.093	0.452	1.21%	0.10%	0.08	NA	NA
Bacteroides	17.76%	18.69%	1.05	0.885	1.000	18.15%	22.97%	1.27	0.647	0.778	18.66%	16.58%	0.89	0.703	0.886	18.63%	19.01%	1.02	0.946	0.957
Bifidobacterium	4.46%	4.90%	1.099	0.851	1.000	3.34%	32.45%	9.71	0.018	0.216	4.38%	33.55%	7.66	0.081	0.452	5.41%	34.44%	6.36	0.029	0.152
Bilophila	0.15%	0.10%	0.66	NA	NA	0.16%	0.03%	0.17	NA	NA	0.35%	0.09%	0.25	NA	NA	0.44%	0.10%	0.24	0.623	0.810
Blautia	13.49%	13.59%	1.01	0.982	1.000	6.53%	4.62%	0.71	0.380	0.608	4.03%	3.86%	0.96	0.931	0.964	3.40%	2.26%	0.67	0.294	0.637
Butyricicoccus	0.17%	0.22%	1.28	0.568	1.000	0.10%	0.03%	0.30	NA	NA	0.09%	0.26%	2.89	0.039	0.280	0.00%	0.17%	Inf	NA	NA
Lachnospiraceae CAG.56	0.79%	0.80%	0.997	0.971	1.000	0.41%	0.33%	0.82	NA	NA	0.05%	0.27%	4.94	NA	NA	0.00%	0.11%	Inf	NA	NA
Clostridia UCG-014 unidentified Genus	1.23%	1.04%	0.85	0.947	1.000	0.45%	0.09%	0.19	NA	NA	0.53%	0.13%	0.26	NA	NA	0.50%	0.21%	0.42	NA	NA
Collinsella	0.07%	0.80%	11.26	NA	NA	0.52%	0.79%	1.52	NA	NA	0.25%	0.86%	3.43	NA	NA	0.53%	0.47%	0.88	NA	NA
Coprococcus	1.13%	1.15%	1.02	0.958	1.000	1.42%	0.66%	0.47	0.015	0.216	4.36%	1.47%	0.34	<0.001	0.007	4.32%	1.48%	0.34	0.005	0.060
Dialister	2.33%	2.22%	0.95	0.950	1.000	2.76%	2.13%	0.77	0.705	0.806	1.96%	2.94%	1.50	0.495	0.717	1.69%	2.45%	1.46	0.541	0.810
Dorea	1.32%	1.29%	0.97	0.723	1.000	1.16%	0.75%	0.65	0.359	0.608	1.85%	1.13%	0.61	0.338	0.637	0.71%	0.52%	0.73	0.579	0.810
Erysipelotrichaceae UCG-003	2.02%	1.82%	0.898	0.698	1.000	2.89%	3.41%	1.18	0.438	0.658	1.86%	0.69%	0.37	<0.001	<0.001	1.22%	0.32%	0.26	NA	NA
Escherichia-Shigella	0.00%	0.00%	NA	NA	NA	27.89%	8.59%	0.31	0.135	0.557	27.53%	12.64%	0.46	0.230	0.607	29.90%	12.21%	0.41	0.160	0.461
Faecalibacterium	9.18%	8.97%	0.98	0.884	1.000	3.93%	2.20%	0.56	0.302	0.557	1.15%	2.03%	1.77	0.373	0.637	0.90%	2.34%	2.59	0.223	0.579

taxon	0h					6h					24h					48h				
	Control (%)	Cubes (%)	Fold-Δ	p-value	q-value	Control (%)	Cubes (%)	Fold-Δ	p-value	q-value	Control (%)	Cubes (%)	Fold-Δ	p-value	q-value	Control (%)	Cubes (%)	Fold-Δ	p-value	q-value
Fusicatenibacter	1.93%	1.84%	0.96	0.901	1.000	1.31%	1.35%	1.03	0.886	0.928	1.08%	1.50%	1.39	0.481	0.717	0.83%	0.86%	1.04	0.957	0.957
Coriobacteriales Incertae Sedis	0.50%	0.49%	0.99	NA	NA	0.22%	0.10%	0.46	NA	NA	0.19%	0.10%	0.53	NA	NA	0.29%	0.15%	0.51	NA	NA
Lachnoclostridium	0.43%	0.40%	0.93	NA	NA	0.67%	0.19%	0.28	NA	NA	6.01%	2.05%	0.34	0.362	0.637	5.99%	2.35%	0.39	0.458	0.794
Lachnospira	0.49%	0.52%	1.08	0.798	1.000	0.18%	0.04%	0.21	NA	NA	0.14%	0.39%	2.82	0.125	0.456	0.14%	0.92%	6.46	0.007	0.060
Lachnospiraceae unidentified Genus	9.64%	9.31%	0.97	0.874	1.000	4.04%	4.35%	1.08	0.483	0.682	2.98%	1.57%	0.53	0.154	0.497	1.83%	1.15%	0.63	0.064	0.278
Lachnospiraceae ND3007 group	1.62%	2.05%	1.26	0.637	1.000	0.89%	0.36%	0.41	0.124	0.557	0.72%	0.40%	0.55	0.312	0.637	0.61%	0.42%	0.70	0.602	0.810
Lachnospiraceae NK4A136 group	1.44%	1.50%	1.04	0.995	1.000	0.64%	0.29%	0.45	0.648	0.778	0.45%	0.42%	0.94	NA	NA	0.43%	0.76%	1.78	0.556	0.810
Lachnospiraceae UCG-004	0.18%	0.18%	0.96	0.950	1.000	0.16%	0.00%	0.00	NA	NA	1.47%	0.56%	0.38	0.005	0.046	1.23%	0.50%	0.41	0.265	0.625
Monoglobus	0.32%	0.36%	1.12	0.828	1.000	0.14%	0.03%	0.24	NA	NA	0.08%	0.25%	3.16	NA	NA	0.09%	0.45%	5.17	NA	NA
Oscillospiraceae unidentified Genus	0.42%	0.29%	0.69	0.232	1.000	0.17%	0.03%	0.15	NA	NA	0.74%	0.18%	0.25	0.182	0.529	0.62%	0.22%	0.36	0.022	0.144
Parabacteroides	0.97%	0.97%	1.00	0.992	1.000	1.93%	0.78%	0.40	0.283	0.557	1.69%	1.07%	0.63	0.310	0.637	2.13%	0.90%	0.42	0.119	0.443
Romboutsia	1.03%	0.94%	0.92	0.975	1.000	0.78%	0.18%	0.23	NA	NA	0.82%	0.24%	0.29	0.126	0.456	0.89%	0.29%	0.32	0.141	0.459
Roseburia	1.85%	1.84%	0.996	0.981	1.000	0.15%	0.13%	0.88	NA	NA	0.00%	0.14%	Inf	NA	NA	0.04%	0.38%	8.92	NA	NA
Ruminococcus	5.38%	4.82%	0.90	0.824	1.000	1.86%	0.81%	0.43	0.214	0.557	1.33%	0.81%	0.61	0.461	0.717	0.82%	1.18%	1.44	NA	NA
Senegalimassilia	0.87%	0.69%	0.80	0.737	1.000	1.41%	0.62%	0.44	0.277	0.557	2.08%	0.97%	0.47	0.290	0.637	2.77%	0.94%	0.34	0.817	0.923
Streptococcus	1.10%	1.03%	0.94	0.884	1.000	0.75%	0.29%	0.38	0.276	0.557	0.67%	0.29%	0.43	0.853	0.916	0.65%	0.31%	0.48	0.322	0.643
Subdoligranulum	5.52%	5.40%	0.98	0.831	1.000	3.66%	3.67%	1.00	0.996	0.996	3.42%	5.10%	1.49	0.994	0.994	4.06%	5.68%	1.40	0.789	0.923

taxon	0h					6h					24h					48h				
	Control (%)	Cubes (%)	Fold-Δ	p-value	q-value	Control (%)	Cubes (%)	Fold-Δ	p-value	q-value	Control (%)	Cubes (%)	Fold-Δ	p-value	q-value	Control (%)	Cubes (%)	Fold-Δ	p-value	q-value
Sutterella	0.89%	0.76%	0.85	0.507	1.000	5.36%	2.07%	0.39	0.266	0.557	2.88%	2.62%	0.91	0.797	0.916	2.54%	2.23%	0.88	0.692	0.857
Oscillospiraceae UCG-002	2.17%	2.00%	0.92	0.967	1.000	0.83%	0.30%	0.36	0.254	0.557	1.31%	0.43%	0.33	0.850	0.916	1.60%	0.72%	0.45	0.367	0.682
Erysipelotrichaceae UCG-003	0.52%	0.45%	0.87	1.000	1.000	0.18%	0.05%	0.30	NA	NA	NA	NA	NA	NA	NA	0.11%	0.12%	1.10	NA	NA
Eubacterium eligens group	0.46%	0.53%	1.15	0.956	1.000	0.20%	0.09%	0.45	NA	NA	0.22%	0.17%	0.80	NA	NA	0.10%	0.56%	5.75	NA	NA
Eubacterium hallii group	1.36%	1.31%	0.96	0.898	1.000	1.18%	2.49%	2.12	0.534	0.712	1.17%	1.58%	1.35	0.640	0.843	1.12%	0.46%	0.41	0.004	0.060
Eubacterium ventriosum group	0.20%	0.23%	1.13	0.854	1.000	0.18%	0.08%	0.46	NA	NA	0.09%	0.00%	NA	NA	NA	0.00%	0.00%	NA	NA	NA
Ruminococcus gauvreauii group	0.59%	0.56%	0.96	0.944	1.000	0.31%	0.09%	0.29	NA	NA	0.26%	0.08%	0.29	NA	NA	0.12%	0.08%	0.67	NA	NA
Ruminococcus torques group	0.97%	0.96%	0.99	0.976	1.000	0.54%	1.78%	3.32	0.890	0.928	0.72%	1.52%	2.10	0.839	0.916	0.76%	1.22%	1.60	NA	NA

fold-Δ: fold-difference in mean relative abundances per timepoint between conditions (dried chicory root cubes fermentation versus control); NA: represents p- and q-values that could not be reliably estimated due to violation of the assumption underlying the statistical models for calculating statistical inference [15]

Supplementary Table 7. pH, gas production and fermentation metabolites measured for each donor and expressed as mean ± SE at baseline (t = 0 h), 6 h, 24 h and 48 h of *in vitro* fermentation of dried chicory root cubes and negative control (only inoculum) to produce fermentation supernatant used in the *ex vivo* Ussing chamber experiment. Fermentation supernatants at t = 48 h were used for the Ussing experiment.

		**Control**				**Dried chicory root cubes**			
		**0h**	**6h**	**24h**	**48h**	**0h**	**6h**	**24h**	**48h**
pH	Donor 1	6.39	6.35	6.36	6.4	6.39	5.82	5.75	5.86
	Donor 2	6.39	6.38	6.35	6.41	6.39	5.73	5.68	5.78
	Donor 3	6.44	6.38	6.44	6.45	6.42	5.97	5.9	5.85
	Donor 4	6.35	6.25	6.28	6.33	6.38	5.62	5.87	5.92
	Mean ± SE	6.39 ± (0.02)	6.34 ± (0.03)	6.36 ± (0.03)	6.4 ± (0.02)	6.4 ± (0.01)	5.79 ± (0.07)	5.8 ± (0.05)	5.85 ± (0.03)
Gas production (mL)	Donor 1	0.00	27.60	36.40	36.40	0.00	76.10	117.00	117.00
	Donor 2	0.00	13.10	36.40	36.40	0.00	36.50	81.10	81.10
	Donor 3	0.00	11.60	27.30	27.30	0.00	34.90	90.00	90.00
	Donor 4	0.00	27.30	27.30	27.30	0.00	106.90	117.00	117.00
	Mean ± SE	0 ± (0)	19.9 ± (4.37)	31.85 ± (2.63)	31.85 ± (2.63)	0 ± (0)	63.6 ± (17.29)	101.28 ± (9.26)	101.28 ± (9.26)
Butyrate (mM)	Donor 1	0.00	2.28	3.33	3.74	0.00	6.68	11.21	11.43
	Donor 2	0.00	2.14	3.40	3.84	1.78	3.20	9.29	9.89
	Donor 3	2.07	2.54	4.10	5.26	0.00	3.35	12.71	12.05
	Donor 4	0.00	2.38	3.47	4.44	1.91	2.30	14.51	16.86
	Mean ± SE	0.52 ± (0.52)	2.33 ± (0.08)	3.57 ± (0.18)	4.32 ± (0.35)	0.92 ± (0.53)	3.88 ± (0.96)	11.93 ± (1.11)	12.56 ± (1.5)
Propionate (mM)	Donor 1	0.00	0.00	3.76	4.02	0.00	10.65	13.60	13.56
	Donor 2	0.00	1.65	3.64	4.01	0.00	9.08	15.45	16.98
	Donor 3	0.00	3.26	3.85	3.55	0.00	4.36	8.13	8.28
	Donor 4	0.00	0.00	3.60	4.99	0.00	0.00	5.96	9.64
	Mean ± SE	0 ± (0)	1.23 ± (0.78)	3.71 ± (0.06)	4.14 ± (0.3)	0 ± (0)	6.02 ± (2.41)	10.79 ± (2.24)	12.12 ± (1.97)

		Control				Dried chicory root cubes			
		**0h**	**6h**	**24h**	**48h**	**0h**	**6h**	**24h**	**48h**
Acetate (mM)	Donor 1	0.00	11.77	17.82	17.85	0.00	26.21	37.35	36.81
	Donor 2	0.00	5.20	17.23	17.60	0.00	21.10	42.08	40.85
	Donor 3	0.00	9.40	17.66	15.75	0.00	15.82	34.30	33.86
	Donor 4	0.00	10.15	17.12	21.31	0.00	24.55	34.77	42.90
	Mean ± SE	0 ± (0)	9.13 ± (1.4)	17.46 ± (0.17)	18.13 ± (1.16)	0 ± (0)	21.92 ± (2.29)	37.12 ± (1.78)	38.6 ± (2.03)
Lactate (mM)	Donor 1	0.00	0.00	0.00	0.00	0.00	3.39	0.00	0.00
	Donor 2	0.00	0.00	0.00	0.00	0.00	4.34	0.00	0.00
	Donor 3	0.00	0.00	0.00	0.00	0.00	4.74	0.00	0.00
	Donor 4	0.00	0.00	0.00	0.00	0.00	10.85	0.00	0.00
	Mean ± SE	0 ± (0)	0 ± (0)	0 ± (0)	0 ± (0)	0 ± (0)	5.83 ± (1.7)	0 ± (0)	0 ± (0)
Iso-butyrate (mM)	Donor 1	0.00	0.00	0.00	0.00	0.00	2.94	2.81	1.98
	Donor 2	0.00	0.00	0.00	0.00	0.00	0.00	2.98	2.23
	Donor 3	0.00	0.00	0.00	0.00	0.00	0.00	0.00	0.00
	Donor 4	0.00	0.00	0.00	0.00	0.00	1.67	2.46	2.71
	Mean ± SE	0 ± (0)	0 ± (0)	0 ± (0)	0 ± (0)	0 ± (0)	1.15 ± (0.71)	2.06 ± (0.7)	1.73 ± (0.6)
Formate (mM)	Donor 1	0.00	0.00	0.00	0.00	0.00	0.00	0.00	0.00
	Donor 2	0.00	0.00	0.00	0.00	0.00	2.28	0.00	0.00
	Donor 3	0.00	0.00	0.00	0.00	0.00	2.25	0.00	0.00
	Donor 4	0.00	0.00	0.00	0.00	0.00	6.54	0.00	0.00
	Mean ± SE	0 ± (0)	0 ± (0)	0 ± (0)	0 ± (0)	0 ± (0)	2.77 ± (1.37)	0 ± (0)	0 ± (0)

Note: Gas production was measured as long as changes in volume (mL) were detected. Values at respective timepoints are therefor maximum levels reached either at this or a previous timepoint (if stagnation in gas production occurred).

Supplementary Table 8. Electrophysiology and gut permeability measures of the Ussing chamber experiment. Electrophysiological and permeability measurements assessed at t = 0 min, 60 min and 90 min assessed during the Ussing experiment in either control or stimulated biopsies using either the stressor (sodium deoxycholate), the fermentation supernatant or their combination. The electrophysiological parameter TER represents the overall gut integrity and is expressed as absolute value or percentage of its baseline. Concentration of FITC at the serosal side of the biopsy is used as a measure of paracellular permeability (between cells) and presented as concentration (nM) at each respective baseline, absolute change (nM) over baseline or relative change (concentration at t = 60 or 90 min divided by each biopsies' respective baseline value).

Measurement	Stimulation	t = 0 min	t = 60 min	t = 90 min
TER (Ω*cm^2)	Control	15.03 ± 2	13.41 ± 1	12.94 ± 2.24
	Stressor	17.68 ± 2.19	14.02 ± 0.78	12.09 ± 0.81
	Fermentation supernatant	16.14 ± 1.60	12.98 ± 1.20	12.43 ± 1.11
	Fermentation supernatant + Stressor	14.07 ± 1.32	10.75 ± 1.62	9.96 ± 1.55
	p-value (between-conditions)	p = 0.64	p = 0.42	p = 0.35
TER absolute Δ (Ω*cm^2)	Control	*n.a*	-1.54 ± 1.09	-2.09 ± 0.64
	Stressor	*n.a*	- 5.54 ± 1.61	- 5.58 ± 1.79
	Fermentation supernatant	*n.a*	-3.15 ± 0.60	-3.71 ± 0.85
	Fermentation supernatant + Stressor	*n.a*	-3.32 ± 0.50	-4.11 ± 0.55
	p-value (between-conditions)	*n.a*	p = 0.05	p = 0.17
TER%	Control	100 ± 0	89.16 ± 6.53	85.02 ± 4.05
	Stressor	100 ± 0	72.18 ± 5.69	70.72 ± 6.77
	Fermentation supernatant	100 ± 0	81.68 ± 2.74	78.47 ± 4.26
	Fermentation supernatant + Stressor	100 ± 0	75.16 ± 5.67	69.72 ± 5.63
	p-value (between-conditions)	*n.a.*	p = 0.07	p = 0.10
FITC (nM)	Control	35.25 ± 6.35	69.35 ± 4.63	72.83 ± 10.99
	Stressor	35.17 ± 6.4	60.66 ± 6.89	84.17 ± 8.31
	Fermentation supernatant	35.09 ± 6.21	64.24 ± 9.61	92.87 ± 6.1
	Fermentation supernatant + Stressor	35.46 ± 6.38	62.96 ± 8.84	86.9 ± 9.59
	p-value (between-conditions)	p = 0.88	p = 0.68	p = 0.12
FITC absolute Δ (nM)	Control	*n.a*	34.1 ± 10.15	32.01 ± 14.69
	Stressor	*n.a*	25.49 ± 7.39	49 ± 9.88
	Fermentation supernatant	*n.a*	29.15 ± 5.53	51.77 ± 6.76
	Fermentation supernatant + Stressor	*n.a*	27.5 ± 6.7	52.79 ± 9.56

Measurement	Stimulation	t = 0 min	t = 60 min	t = 90 min
	p-value (between-conditions)	*n.a*	*p* = 0.70	*p* = 0.13
FITC fold-Δ	Control	*n.a*	2.31 ± 0.66	1.9 ± 0.49
	Stressor	*n.a*	1.88 ± 0.31	2.67 ± 0.53
	Fermentation supernatant	*n.a*	1.91 ± 0.19	2.28 ± 0.21
	Fermentation supernatant + Stressor	*n.a*	1.89 ± 0.26	2.68 ± 0.45
	p-value (between-conditions)	*n.a*	*p* = 0.50	*p* = 0.44

TER: Transepithelial resistance; TER%: percentage change in TER over each biopsies respective baseline; FITC: Fluorescein isothiocyanate–dextran; absolute Δ: total change in nM over each biopsies respective baseline; fold-Δ: relative change over each biopsies respective baseline (respective level expressed as factor of its baseline)

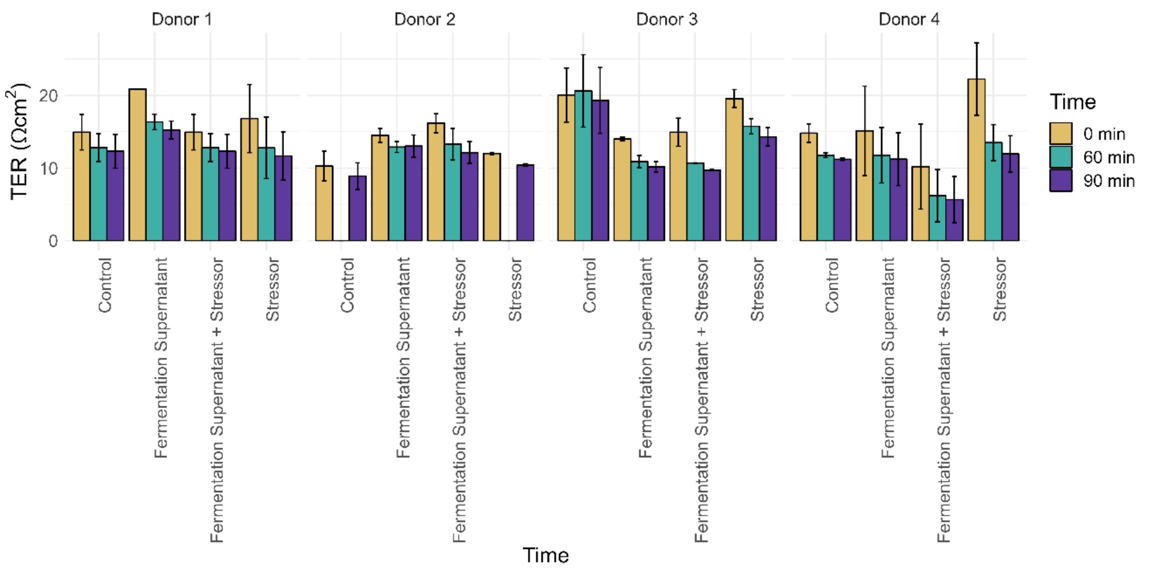

A

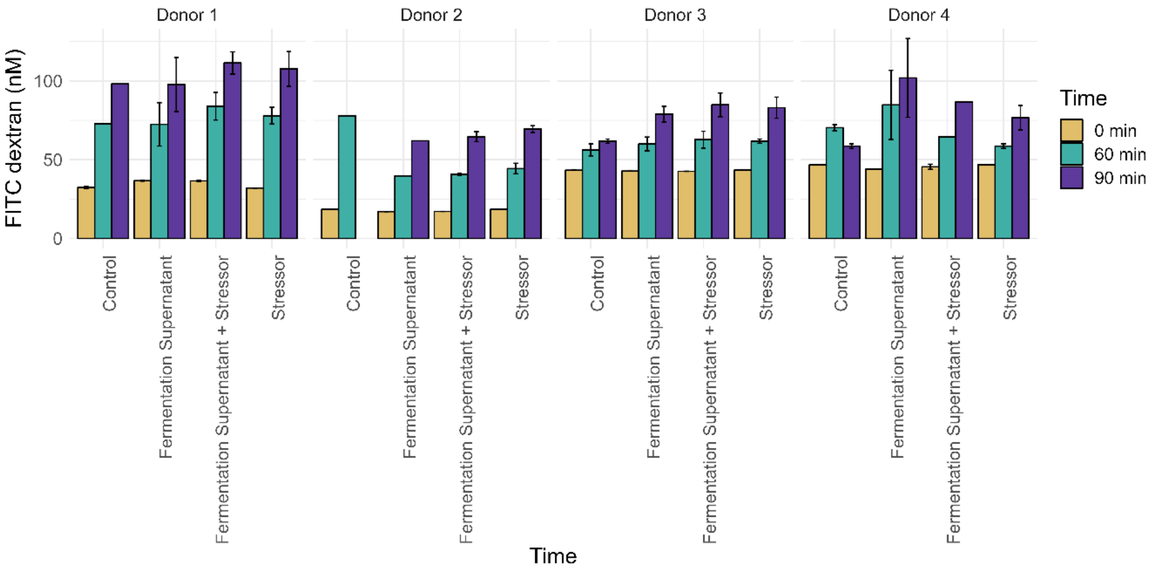

B

Supplementary Figure 12. Individual changes over time in electrophysiology and gut permeability measures of the Ussing chamber experiment. A: Overall gut integrity measured by transepithelial resistance (TER) at t = 0 min, 60 min and 90 min in each donors‘ biopsies. B: Paracellular permeability measured by FITC-dextran (Fluorescein isothiocyanate–dextran) at the serosal side at t = 0 min, 60 min and 90 min in each donors‘ biopsies.

References

1. Minekus M, Alminger M, Alvito P, Ballance S, Bohn T, Bourlieu C, et al. A standardised static in vitro digestion method suitable for food – an international consensus. *Food Funct* 2014;5:1113–24. [PMID: 24803111 DOI: 10.1039/c3fo60702j]

2. Brodkorb A, Egger L, Alminger M, Alvito P, Assunção R, Ballance S, et al. INFOGEST static in vitro simulation of gastrointestinal food digestion. *Nat Protoc* 2019;14:991–1014. [PMID: 30886367 DOI: 10.1038/s41596-018-0119-1]

3. Thibault JF. Automated-method for the determination of pectic substances. *Leb und -Technologie* 1979;12:247–251.

4. Ramasamy US, Gruppen H, Schols HA. Structural and Water-Holding Characteristics of Untreated and Ensiled Chicory Root Pulp. *J Agric Food Chem* 2013;61:6077–85. [PMID: 23725019 DOI: 10.1021/jf401621h]

5. Logtenberg MJ, Akkerman R, An R, Hermes GDA, de Haan BJ, Faas MM, et al. Fermentation of Chicory Fructo-Oligosaccharides and Native Inulin by Infant Fecal Microbiota Attenuates Pro-Inflammatory Responses in Immature Dendritic Cells in an Infant-Age-Dependent and Fructan-Specific Way. *Mol Nutr Food Res* 2020;64:2000068. [PMID: 32420676 DOI: 10.1002/mnfr.202000068]

6. Van Den Abbeele P, Belzer C, Goossens M, Kleerebezem M, De Vos WM, Thas O, et al. Butyrate-producing Clostridium cluster XIVa species specifically colonize mucins in an in vitro gut model. *ISME J* 2012;7:949–61. [PMID: 23235287 DOI: 10.1038/ismej.2012.158]

7. De Weirdt R, Possemiers S, Vermeulen G, Moerdijk-Poortvliet TCW, Boschker HTS, Verstraete W, et al. Human faecal microbiota display variable patterns of glycerol metabolism. *FEMS Microbiol Ecol* 2010;74:601–11. [PMID: 20946352 DOI: 10.1111/J.1574-6941.2010.00974.X]

8. Van de Wiele T, Boon N, Possemiers S, Jacobs H, Verstraete W. Prebiotic effects of chicory inulin in the simulator of the human intestinal microbial ecosystem. *FEMS Microbiol Ecol* 2004;51:143–53. [PMID: 16329863 DOI: 10.1016/j.femsec.2004.07.014]

9. Tabat MW, Marques TM, Markgren M, Löfvendahl L, Brummer RJ, Wall R. Acute Effects of Butyrate on Induced Hyperpermeability and Tight Junction Protein Expression in Human Colonic Tissues. *Biomolecules* 2020;10:766. [PMID: 32422994 DOI: 10.3390/biom10050766]

10. Parada AE, Needham DM, Fuhrman JA. Every base matters: assessing small subunit rRNA primers for marine microbiomes with mock communities, time series and global field samples. *Environ Microbiol* 2016;18:1403–14. [PMID: 26271760 DOI: 10.1111/1462-2920.13023]

11. Apprill A, Mcnally S, Parsons R, Weber L. Minor revision to V4 region SSU rRNA 806R gene primer greatly increases detection of SAR11 bacterioplankton. *Aquat Microb Ecol* 2015;75:129–37. [DOI: 10.3354/ame01753]

12. Poncheewin W, Hermes GDA, van Dam JCJ, Koehorst JJ, Smidt H, Schaap PJ. NG-Tax 2.0: A Semantic Framework for High-Throughput Amplicon Analysis. *Front Genet* 2020;10:1366.

[PMID: 32117417 DOI: 10.3389/fgene.2019.01366]

13. Quast C, Pruesse E, Yilmaz P, Gerken J, Schweer T, Yarza P, et al. The SILVA ribosomal RNA gene database project: improved data processing and web-based tools. *Nucleic Acids Res* 2013;41:D590–6. [PMID: 23193283 DOI: 10.1093/nar/gks1219]

14. Mensink MA, Frijlink HW, van der Voort Maarschalk K, Hinrichs WLJ. Inulin, a flexible oligosaccharide I: Review of its physicochemical characteristics. *Carbohydr Polym* 2015;130:405–19. [DOI: https://doi.org/10.1016/j.carbpol.2015.05.026]

15. Korpela K. mare: Microbiota Analysis in R Easily. R package version 1.0. 2016. Available from: https://github.com/katrikorpela/mare. [Last accessed 17 Jun 2021]

16. Ouwerkerk JP, De Vos WM, Belzer C. Glycobiome: Bacteria and mucus at the epithelial interface. *Best Pract Res Clin Gastroenterol* 2013;27:25–38. [PMID: 23768550 DOI: 10.1016/j.bpg.2013.03.001]
